# A “Genome-to-Lead” Approach for Insecticide Discovery: Pharmacological Characterization and Screening of *Aedes aegypti* D_1_-like Dopamine Receptors

**DOI:** 10.1371/journal.pntd.0001478

**Published:** 2012-01-24

**Authors:** Jason M. Meyer, Karin F. K. Ejendal, Larisa V. Avramova, Elisabeth E. Garland-Kuntz, Gloria I. Giraldo-Calderón, Tarsis F. Brust, Val J. Watts, Catherine A. Hill

**Affiliations:** 1 Department of Entomology, Purdue University, West Lafayette, Indiana, United States of America; 2 Department of Medicinal Chemistry and Molecular Pharmacology, Purdue University, West Lafayette, Indiana, United States of America; 3 Integrated Screening Technologies, Bindley Bioscience Center, Discovery Park, Purdue University, West Lafayette, Indiana, United States of America; Johns Hopkins Bloomberg School of Public Health, United States of America

## Abstract

**Background:**

Many neglected tropical infectious diseases affecting humans are transmitted by arthropods such as mosquitoes and ticks. New mode-of-action chemistries are urgently sought to enhance vector management practices in countries where arthropod-borne diseases are endemic, especially where vector populations have acquired widespread resistance to insecticides.

**Methodology/Principal Findings:**

We describe a “genome-to-lead” approach for insecticide discovery that incorporates the first reported chemical screen of a G protein-coupled receptor (GPCR) mined from a mosquito genome. A combination of molecular and pharmacological studies was used to functionally characterize two dopamine receptors (*Aa*DOP1 and *Aa*DOP2) from the yellow fever mosquito, *Aedes aegypti*. Sequence analyses indicated that these receptors are orthologous to arthropod D_1_-like (Gα_s_-coupled) receptors, but share less than 55% amino acid identity in conserved domains with mammalian dopamine receptors. Heterologous expression of *Aa*DOP1 and *Aa*DOP2 in HEK293 cells revealed dose-dependent responses to dopamine (EC_50_: *Aa*DOP1 = 3.1±1.1 nM; *Aa*DOP2 = 240±16 nM). Interestingly, only *Aa*DOP1 exhibited sensitivity to epinephrine (EC_50_ = 5.8±1.5 nM) and norepinephrine (EC_50_ = 760±180 nM), while neither receptor was activated by other biogenic amines tested. Differential responses were observed between these receptors regarding their sensitivity to dopamine agonists and antagonists, level of maximal stimulation, and constitutive activity. Subsequently, a chemical library screen was implemented to discover lead chemistries active at *Aa*DOP2. Fifty-one compounds were identified as “hits,” and follow-up validation assays confirmed the antagonistic effect of selected compounds at *Aa*DOP2. *In vitro* comparison studies between *Aa*DOP2 and the human D_1_ dopamine receptor (hD_1_) revealed markedly different pharmacological profiles and identified amitriptyline and doxepin as *Aa*DOP2-selective compounds. In subsequent *Ae. aegypti* larval bioassays, significant mortality was observed for amitriptyline (93%) and doxepin (72%), confirming these chemistries as “leads” for insecticide discovery.

**Conclusions/Significance:**

This research provides a “proof-of-concept” for a novel approach toward insecticide discovery, in which genome sequence data are utilized for functional characterization and chemical compound screening of GPCRs. We provide a pipeline useful for future prioritization, pharmacological characterization, and expanded chemical screening of additional GPCRs in disease-vector arthropods. The differential molecular and pharmacological properties of the mosquito dopamine receptors highlight the potential for the identification of target-specific chemistries for vector-borne disease management, and we report the first study to identify dopamine receptor antagonists with *in vivo* toxicity toward mosquitoes.

## Author Summary

Mosquitoes and other arthropods transmit important disease-causing agents affecting human health worldwide. There is an urgent need to discover new chemistries to control these pests in order to reduce or eliminate arthropod-borne diseases. We describe an approach to identify and evaluate potential insecticide targets using publicly available genome (DNA) sequence information for arthropod disease vectors. We demonstrate the utility of this approach by first determining the molecular and pharmacological properties of two different dopamine (neurotransmitter) receptors of the yellow fever- and dengue-transmitting mosquito, *Aedes aegypti*. Next, we tested 1,280 different chemistries for their ability to interact with one of these dopamine receptors in a chemical screen, and 51 “hit” compounds were identified. Finally, we show that two of these chemistries, amitriptyline and doxepin, are selective for the mosquito over the human dopamine receptor and that both chemistries caused significant mortality in mosquito larvae 24 hours after exposure, identifying them as possible “leads” for insecticide development. Our methodology is adaptable for chemical screening of related targets in mosquitoes and other arthropod vectors of disease. This research demonstrates the potential of target-specific approaches that could complement traditional phenotypic screening, and ultimately may accelerate discovery of new mode-of-action insecticides for vector control.

## Introduction

Mosquitoes (Class Insecta; Order Diptera; Family Culicidae) vector multiple neglected tropical diseases (NTDs) affecting human health, including malaria, yellow-fever, dengue and filariasis. Historically, insecticides employed against arthropod disease vectors have reduced the impact of NTDs, but unfortunately, continued disease control is threatened by the widespread development of vector populations that are resistant to insecticidal chemistries [1]. This issue is further complicated by the fact that there has not been a new public health insecticide produced for vector-borne disease control for over 30 years [2]. Recently, philanthropic investment has focused attention toward the development of new drugs to control NTDs in the human population [3]. It is widely recognized that an arsenal of new vector control solutions are required in order to meet this and other public health goals regarding NTDs. Thus, the research community should aggressively pursue the discovery of new mode-of-action chemistries for mosquito control through both traditional phenotypic screening and target-based approaches.

Novel insecticide targets potentially exist among the arthropod G protein-coupled receptors (GPCRs). These proteins comprise a large family of membrane-bound molecules that mediate critical biological processes such as neurotransmission, vision, and hormonal regulation, among others [4], [5]. GPCRs are extensively targeted for drug development in humans - approximately 40% of prescription pharmaceuticals interact with these receptors [6] - and more recently, Gamo et al. [7] reported multiple GPCR-interacting chemistries as promising anti-malarial leads. Also, the mode-of-action of amitraz, a chemistry registered for tick and insect control, is presumed to have partial agonistic activity at an octopamine sensitive GPCR [8]. More than 100 different GPCRs have been identified in the genomes of multiple insect species, including malaria- and yellow fever-transmitting mosquitoes [9], [10]. These studies have provided a basis for the functional characterization of GPCRs and their prioritization as potential subjects for insecticide development.

The biogenic amine-binding GPCRs (rhodopsin-like) are integral components of the central and peripheral nervous systems of eukaryotes and include receptors that bind the neurotransmitters dopamine, histamine, octopamine, serotonin, tyramine, and acetylcholine [11]. The dopamine receptors are classified as either D_1_- or D_2_-like [12] based on their differential functional roles. Ligand binding to the D_1_-like dopamine receptors causes Gα_s_-mediated stimulation of adenylyl cyclase (AC) production of cAMP. A reciprocal effect is observed following agonist activation of D_2_-like dopamine receptors, whereby cAMP production by AC is inhibited via Gα_i/o_ proteins. Dopamine and its receptors are essential for complex behavioral mechanisms in arthropods such as locomotion [13], [14], [15], arousal [16], and olfactory learning [17], [18].

The importance of dopaminergic-related functions has stimulated research to understand these processes in mosquitoes. Dopamine and serotonin have been tied to salivary gland functioning of vectors [19], [20] and may have an impact on pathogen acquisition and transmission during blood feeding. Andersen et al. [21] reported that increased levels of dopamine were detected in *Aedes aegypti* following a blood meal that were implicated in ovarian or egg development, and in newly-emerged adults, presumably as part of the sclerotization process. Much attention has been given to the role of dopamine in the melanization pathway of mosquitoes and other insects, as well as the effect of dopamine on development, pigmentation, reproduction, immune responses to parasites, wound healing, and *Wolbachia* infection [22], [23], [24], [25], [26], [27]. In the mosquito *Culex pipiens*, dose-dependent increases in cAMP were detected following treatment with dopamine and octopamine in homogenized head tissues, suggesting the presence of Gα_s_-coupled receptors that are responsive to these biogenic amines [28]. Putative D_1_-like and D_2_-like dopamine receptors have been identified in the genomes of the mosquitoes *Ae. aegypti* [9] and *Anopheles gambiae* [10], but research investigating their pharmacological properties is lacking. These genomic sequences provide a logical starting point to functionally characterize the receptors, which is needed to improve our comprehension of dopaminergic processes in mosquitoes. Moreover, due to their presumed significance in mosquito neurobiology, these dopamine receptors are attractive candidates to explore as new targets for chemical control.

**Figure 1:**
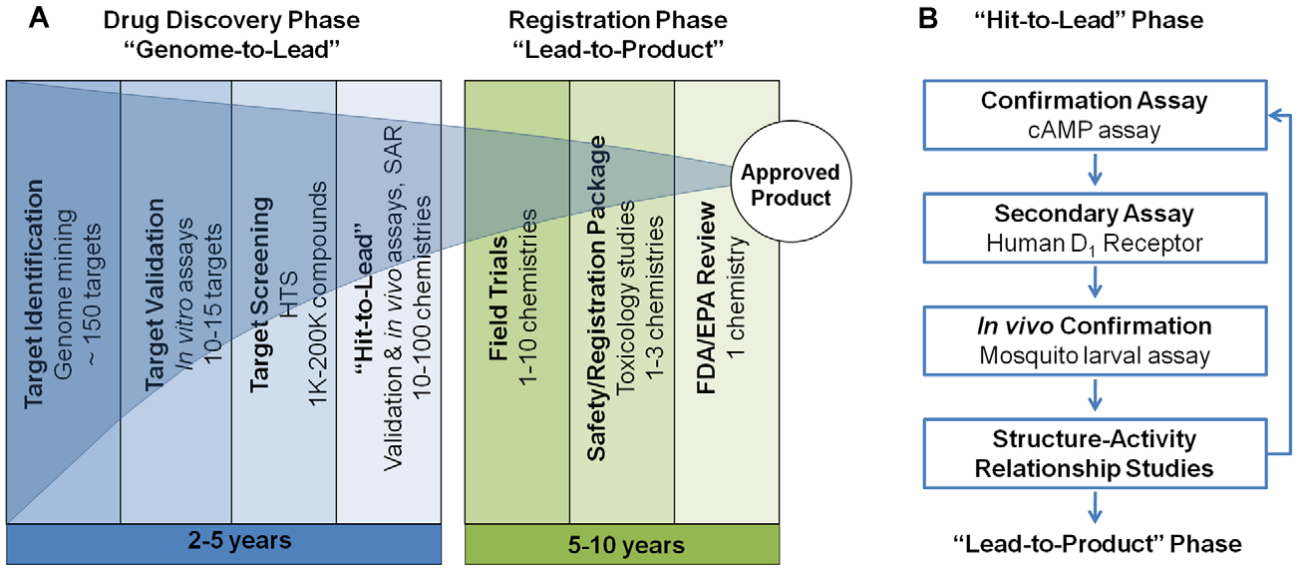
Drug discovery and development pipeline for new insecticidal chemistries. **A:** The illustration shows critical steps involved with the “genome-to-lead” (described in this manuscript) and “lead-to-product” phases. Abbreviations: (EPA) Environmental Protection Agency; (FDA) Food and Drug Administration; (SAR) structure-activity relationship study. The intended administration route of a particular chemistry dictates the federal agency that will receive the registration package; **B:** Expanded details of the “hit-to-lead” phase including those pursued in this study.

We present the results of a “proof-of-concept” study involving a “genome-to-lead” approach for developing new mode-of-action insecticides for arthropod disease vectors (Figure 1A). Our research strategy involves (i) exploitation of an arthropod genome sequence for novel target identification, (ii) molecular, biochemical and pharmacological target validation, (iii) chemical library screening, and (iv) confirmation of hits and identification of candidate “leads” using secondary *in vitro* assays and mosquito *in vivo* assays. Toward these objectives, two dopamine receptors (*Aa*DOP1 and *Aa*DOP2) were identified in the genome of the yellow-fever mosquito, *Ae. aegypti*, and characterized using molecular and pharmacological methods. Subsequently, we conducted a chemical library screen in which multiple lead antagonistic chemistries of the *Aa*DOP2 receptor were identified. Finally, we employed a “hit-to-lead” approach (Figure 1B), wherein screen “hits” were confirmed in secondary *in vitro* assays and two “lead” chemistries were identified using *in vivo* assays that confirmed their toxicity to mosquito larvae. These results serve as an entry point for expanded chemical library screening of mosquito dopamine receptors and subsequent structure-activity relationship- and further “hit-to-lead”-studies to discover candidate compounds that will enter the registration phase of product development (Figure 1A). Our pipeline will expedite the exploration of GPCRs as potential targets for chemical control in mosquitoes and other important arthropod disease vectors for which sufficient genome sequence data is available.

## Materials and Methods

### Molecular analyses

The gene sequences for the putative dopamine receptors AaegGPRdop1 (AAEL003920) and AaegGPRdop2 (AAEL005834) (referred to hereafter as *Aadop1* and *Aadop2*, respectively) in *Ae. aegypti* [10] were downloaded from VectorBase (http://www.vectorbase.org/index.php) [29]. Sequences of the D_1_-like dopamine receptors in *Drosophila melanogaster* were used to identify and compare conserved structural features [30], [31].

Gene expression analyses for each receptor were conducted using RNA extracted from the eggs, larvae, pupae, and adult male and female mosquitoes from the Liverpool strain of *Ae. aegypti* [10]. Total RNA was isolated using TRIzol Reagent (Invitrogen, Carlsbad, CA) and then treated with RNase-Free DNase (QIAGEN, Valencia, CA). The SuperScript One-Step RT-PCR kit (Invitrogen, Carlsbad, CA) was used to amplify receptor mRNA from approximately 150 ng total RNA per reaction using the primers and experimental conditions provided in Table S1. RT-PCR amplification products were electrophoresed and compared by size to the DNA HyperLadder I (Bioline USA Inc., Randolph, MA). Products were cut from the gel and isolated with the Qiagen Gel Extraction Kit (Qiagen Valencia, CA). The cloning procedure was performed using the TOPO TA cloning kit (Invitrogen, Carlsbad, CA), according to the manufacturer's instructions. DNA sequencing was conducted at the Purdue University Genomics Core Facility. The resultant DNA sequences were used to predict full-length coding regions that were manually annotated using Artemis software (version 9) [32].

A neighbor-joining sequence analysis was performed using the deduced amino acid sequences representing the mosquito dopamine receptor proteins (referred to hereafter as *A*aDOP1 and *Aa*DOP2), additional representative biogenic amine receptors from the insects *D. melanogaster* and *A. mellifera*, and the human D_1_- and D_2_-like dopamine receptors. ClustalW 1.83 [33] was used for sequence alignments prior to tree construction in PAUP 4.0b4a [34]. The bootstrap method (100 replicates) was used to provide branch support. Alignments of amino acid sequences for determination of conserved motifs were conducted using Multalin software [35]. Conserved amino acid residues and additional protein features were predicted as described by Meyer et al. [36].

### Heterologous expression

Functional characterization of *Aa*DOP1 and *Aa*DOP2 was conducted by heterologous expression in HEK293 cells (ATCC, Manassas, VA) [36]. Expression constructs were produced by synthesis (GenScript, Piscataway, NJ) and included the partial Kozak transcriptional recognition sequence “CACC” added directly upstream of the transcription initiation codon for each gene. Constructs were cloned into pUC57 and then subcloned into the expression vector pcDNA3.1+ (Invitrogen, Carlsbad, CA) by GenScript (Piscataway, NJ). Stable cell lines co-expressing either *Aa*DOP1 or *Aa*DOP2 with a CRELuc reporter construct were developed to permit pharmacological studies in a 384-well format [36], [37]. Briefly, cells already stably expressing the CRELuc reporter construct were transfected in a 10 cm dish with 15 µl Lipofectamine2000 and 3 µg of pcDNA3.1+/*Aadop1* or pcDNA3.1+/*Aadop2*. Clones were maintained as described for the wild-type HEK293 cells [36] with the addition of 2 µg/ml puromycin and 300 µg/ml Geneticin (Sigma-Aldrich, St. Louis, MO).

### Pharmacological characterization

For initial functional analysis, the receptors were transiently expressed in HEK293 cells [36] and analyzed using a competitive binding assay to measure levels of cAMP accumulation [37]. Dose-response curves were generated using cells stably expressing the receptors [36], [37]. The compounds used for pharmacological characterization included dopamine hydrochloride, histamine dihydrochloride, 5-hydroxytryptamine hydrochloride (serotonin), (±)-octopamine hydrochloride, tyramine hydrochloride (Sigma-Aldrich, St. Louis, MO), (−)-epinephrine bitartrate, and L (−)-norepinephrine bitartrate (Research Biochemical International, Natick, MA). The synthetic dopamine receptor ligands tested included SKF38393 and SKF81297 (Tocris, Ellisville, MO), SCH23390 (Tocris, Ellisville, MO), and dihydrexidine (DHX) (a gift from D. Nichols, Purdue University). Data was collected from a minimum of three independent replicate experiments with each sample measured in triplicate. Statistical analysis of data was conducted with GraphPad Prism 5 software (GraphPad Software Inc., San Diego, CA).

### Screening of *Aa*DOP2 against the LOPAC_1280_ library

To identify novel *Aa*DOP2 receptor antagonists, the Library of Pharmacologically Active Compounds (LOPAC_1280_) was screened at the Integrated Screening Technologies Laboratory, Discovery Park, Purdue University, using HEK-CRELuc-*Aa*dop2 cells. These cells were cultured as described above, expanded, and cryo-preserved, to produce a uniform cell population. Briefly, cells (∼2.5×10^7^) were harvested by non-enzymatic dissociation [0.5 mM EDTA in Ca^2+^Mg^2+^free-phosphate buffered saline (CMF-PBS)] resuspended in cell culture media, and pelleted by centrifugation for 5 min at 100× G. The pellet was resuspended in freezing media (Opti-MEM supplemented with 10% DMSO and 20% FBS) to a concentration of 5×10^6^/ml, frozen step-wise, and held in liquid N_2_ until use. Cells were rapidly thawed, diluted in Opti-MEM, and 20 µl containing 25,000 cells were plated per well in 384-well plates (Nunc, Fisher Scientific, Pittsburgh, PA) using a BiomekFX liquid handling station (Beckman-Coulter, Brea, CA). The plates were incubated overnight in a humidified incubator at 37°C and 5% CO_2_.

Prior to screen initiation, a “checkerboard” analysis was conducted that included a minimum (300 nM dopamine in combination with 10 µM SCH23390) and maximum (300 nM dopamine) stimulatory condition. The data obtained were analyzed to calculate the Z-factor [38] using a modified equation that accounts for the number of replicates (NIH website: http://assay.nih.gov/assay/index.php/Section2:Plate_Uniformity_and_Signal_Variability_Assessment).

All compounds were diluted to appropriate concentrations and suspended in assay buffer (Opti-MEM supplemented with 0.02% ascorbic acid) using a BiomekFX 96-tip head. All LOPAC_1280_ compounds were screened in quadruplicate at a concentration of 10 µM, including duplicate samples on two separate assay plates in different quadrants to control for plate and automation effects. Each plate contained a dopamine response curve (14 nM–30 µM) and antagonist control wells (10 µM SCH23390 in combination with 300 nM dopamine). Following compound addition, dopamine was added to each test well at a final concentration of 300 nM, and cells were incubated for 2 hr at 37°C in a humidified incubator. The plates were then equilibrated at 25°C prior to the addition of Steadylite plus luminescence reagent (PerkinElmer, Shelton, CT). Plates were incubated on a shaker at 300 rpm for 5 min, and the luminescence signal was measured using a DTX880 multimode reader (Beckman Coulter, Brea, CA) with a 1 sec integration time.

Raw screen data were processed as follows: the average background luminescence (cells in the absence of dopamine or LOPAC_1280_ compound) was subtracted from the raw data. Values for the positive receptor activation control (300 nM dopamine) were averaged within each assay plate and used to establish a 100% dopamine receptor stimulation level. Similarly, the average response to SCH23390 was calculated within each assay plate to establish a baseline inhibition for antagonist chemistries. The average percent compound effect was calculated for each LOPAC chemistry in comparison to the SCH23390 antagonist control. The minimum criterion for selection of an antagonist “hit” was established as the percent inhibition equivalent to that determined for SCH23390+3 standard deviations.

### “Hit-to-lead” studies

#### Confirmation and secondary *in vitro* assays

Subsequent validation assays using both the AaDOP2 and the human D_1_ dopamine receptor (hD_1_) [39] were conducted for select identified “hit” chemistries using a competitive binding cAMP accumulation assay. In addition to SCH23390, these included amitriptyline hydrochloride, doxepin hydrochloride, niclosamide, clozapine, (+)-butaclamol hydrochloride, cis-(Z)-flupenthixol dihydrochloride, resveratrol, mianserin hydrochloride (Sigma, St. Louis, MO), piceatannol and methiothepin maleate (Tocris, Ellisville, MO). The drugs were suspended from dimethyl sulfoxide (DMSO) stocks in Hanks Balanced Salt Solution (HBSS) (HyClone, Logan, UT) with with 0.1% fatty acid free bovine serum albumin (BSA) and 20 mM 4-(2-hydroxyethyl)-1-piperazineethanesulfonic acid (HEPES), and serial dilutions were prepared using a Precision 2000 automated pipetting system (BioTek, Winooski, VT). The cAMP accumulation assay was carried out as previously described [36], [37] with minor modifications to permit processing of a larger number of samples in a semi-automated fashion. Briefly, AaDOP2- or hD1-expressing cells were harvested using Hank's based non-enzymatic cell dissociation reagent (Invitrogen, Carlsbad, CA), resuspended in Dulbecco's modified eagle medium (DMEM) (Invitrogen, Carlsbad, CA), centrifuged 5 min at 100× G, and resuspended in HBSS supplemented with 0.1% BSA and 20 mM HEPES. Cells were seeded (50,000 cells in 40 µl) in clear 96-well plates and incubated at 37°C with 5% CO_2_ for 1 hr. The cAMP accumulation assay was carried out in HBSS supplemented with final concentrations of 0.1% BSA, 20 mM HEPES, 0.5 mM 3-isobutyl-1-methylxanthine (IBMX), and 0.02% ascorbic acid in a final volume of 50 µl. The selected compounds were added to the wells in duplicate, followed by addition of dopamine (final concentration 3 µM for AaDOP2 and 100 nM for hD_1_). Plates were incubated at room temperature for 1 hr, and the assay was terminated by addition of 25 µl of 9% ice-cold trichloroacetic acid (TCA). Cell lysates were incubated on ice for at least 1 hr prior to quantifying cAMP accumulation as previously described [36], [37].

### *In vivo Ae. aegypti* bioassays

Single dose-point and dose response *in vivo* mosquito bioassays were used to assess the toxicity of selected *Aa*DOP2 receptor antagonists identified in the chemical screen. Larvae of *Ae. aegypti* (Liverpool strain) were reared under standard laboratory conditions on a 12 hr day/night cycle at 75% RH and 28°C, and bioassays were conducted at room temperature (22–24°C). Larvae were transferred from standard rearing trays into six-well tissue culture plates (Corning, Inc. Corning, NY) using a small plastic pipette. Ten L4-stage larvae were included per well, each containing five ml of de-ionized water and the assigned drug concentration. Controls were conducted similarly but lacked a drug treatment. Bioassays employed a double-blind experimental design, and percent mortality was scored 24 hr following administration of drugs. Single dose-point assays were conducted using 400 µM drug and included three biological replicates each consisting of 50–100 larvae. Dose-response assays were conducted using five doses (400, 200, 100, 50, and 25 µM) of the compounds suspended in water, with water alone as a control. Five technical replicates, each including 10 larvae, were performed per dose, and the assay was repeated three times. Statistical analyses included one sample t-tests (single-point assays) and determination of the LC_50_ and LC_90_ values (dose-response assays) conducted with GraphPad Prism 5 software (GraphPad Software Inc., San Diego, CA).

## Results

### Molecular analyses

**Figure 2:**
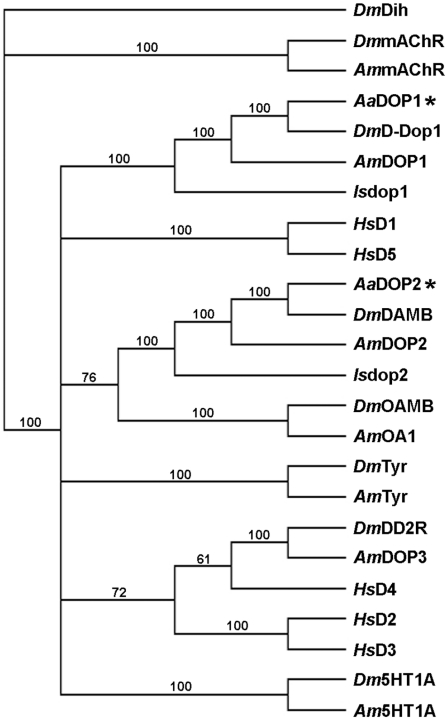
Neighbor-joining sequence analysis of *Aedes aegypti Aa*DOP1 and AaDOP2 and representative biogenic amine receptors. The deduced amino acid sequences for the mosquito dopamine receptors *Aa*DOP1 and AaDOP2 and additional receptors for dopamine, muscarinic acetylcholine, octopamine, serotonin, and tyramine from *Drosophila melanogaster* and *Apis mellifera*, as well as the human D_1_-like and D_2_-like dopamine receptors were aligned for use in the analysis. Bootstrap values (100 replicates) are indicated with numbers at supported branches. The outgroup is a *D. melanogaster* diuretic hormone receptor, a Class B GPCR. Abbreviations: *Aa* = *Ae. aegypti*; *Is* = *I. scapularis*; *Dm* = *D. melanogaster*; *Am* = *A. mellifera*; *Hs* = *H. sapiens*. Sequences: *Is*dop1, D_1_-like dopamine receptor (ISCW001496); *Is*dop2, D_1_-like dopamine receptor (ISCW008775); *Dm*D-Dop1, D_1_-like dopamine receptor (P41596); *Dm*DAMB, D_1_-like dopamine receptor (DopR99B/DAMB: AAC47161), *Dm*DD2R, D_2_-like dopamine receptor (DD2R-606: AAN15955); *Dm*Dih, diuretic hormone 44 receptor 1 (NP_610960.1); *Dm*mAChR, muscarinic acetylcholine receptor (AAA28676); *Dm*OAMB, octopamine receptor in mushroom bodies, isoform A (NP_732541); DM5HT1A, serotonin receptor 1A, isoform A (NP_476802); *Dm*Tyr, tyramine receptor (CG7431: NP_650652); *Am*DOP1, D_1_-like dopamine receptor (dopamine receptor, D1, NP_001011595); *Am*DOP2, D_1_-like dopamine receptor (dopamine receptor 2, NP_001011567), *Am*DOP3, D_2_-like dopamine receptor (*Am*DOP3, NP_001014983); *Am*mAChR, muscarinic acetylcholine receptor (XP_395760); *Am*OA1, octopamine receptor (oar, NP_001011565); *Am*5HT1A, serotonin receptor (5ht-1, NP_001164579); *Am*Tyr, tyramine receptor (XP_394231); *Hs*D1, D_1_-like dopamine receptor (D(1A), NP_000785); *Hs*D2,D_2_-like dopamine receptor (D(2), NP_000786); *Hs*D3, D_2_-like dopamine receptor (D(3), NP_000787); *Hs*D4, D_2_-like dopamine receptor (D(4), NP_000788); *Hs*D5, D_1_-like dopamine receptor (D(1B)/D5, NP_000789).

mRNA transcripts for *Aadop1* and *Aadop2* were detected by RT-PCR in eggs, larvae, pupae, and adult male and female *Ae. aegypti* (Figure S1). DNA sequencing of RT-PCR products confirmed the splice junctions at each intron/exon boundary for both receptor genes. Using a combination of evidence from our RT-PCR data, the genome sequence, and related sequences in *D. melanogaster*, we predicted the gene structure and complete coding regions of *Aadop1* (Genbank accession: JN043502) and *Aadop2* (Genbank accession: JN043503) (Figure S2). A neighbor-joining sequence analysis was conducted to assess the relationships of *Aa*DOP1 and *Aa*DOP2 with other representative biogenic amine receptors (Figure 2). *Aa*DOP1 was included in a clade (bootstrap = 100) containing the presumably orthologous D_1_-like dopamine receptors D-Dop1 of *D. melanogaster* [30], [40], DOP1 of *A. mellifera* [41], and *Is*dop1 of *I. scapularis* [36], [42]. *Aa*DOP2 clustered with two presumably orthologous insect D_1_-like dopamine receptors (INDRs) [43], DopR99B (DAMB) of *D. melanogaster* [31], [44] and DOP2 of *A. mellifera* [41], as well as *Is*dop2 of *I. scapularis* [36]. The INDR-like and *Is*dop2 sequences were also joined together in a larger clade (bootstrap = 76) containing the octopamine receptors OAMB of *D. melanogaster* [45] and OCT1 [46] of *A. mellifera*, consistent with Mustard et al. [41]. The human D_1_-like dopamine receptors formed a separate clade (bootstrap = 100) distinct from the arthropod dopamine receptors.

**Figure 3:**
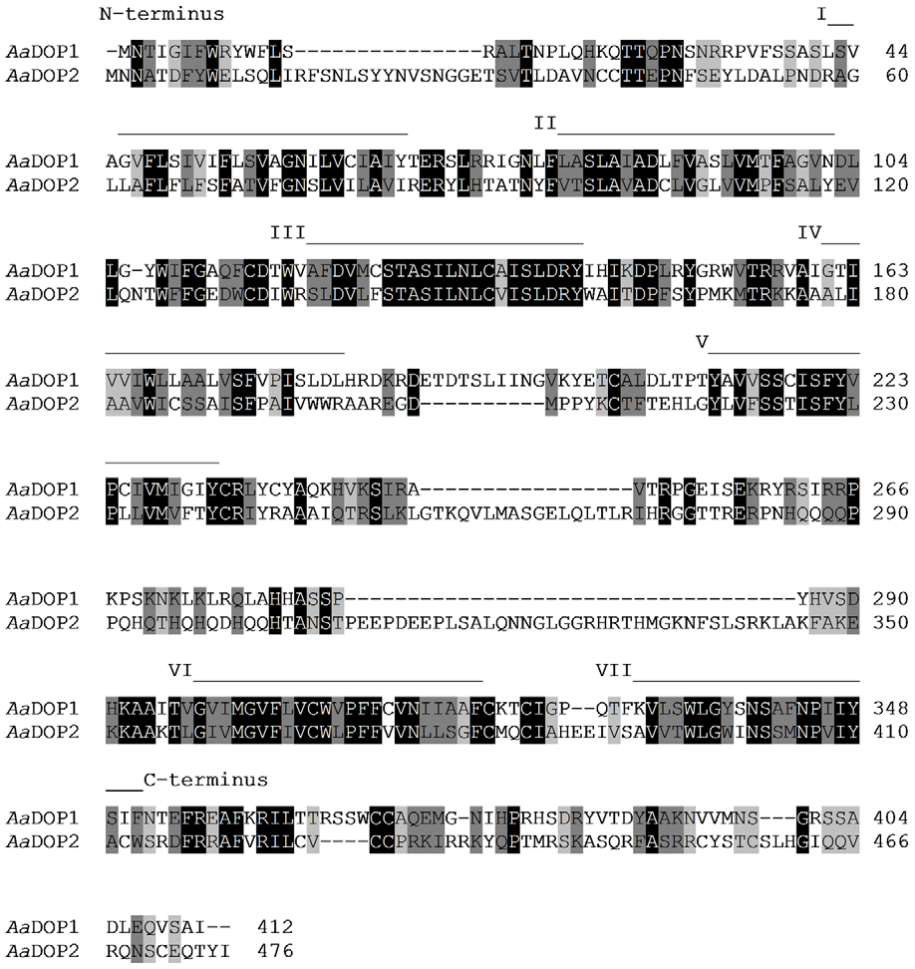
Alignment of the complete *Aedes aegypti Aa*DOP1 and *Aa*DOP2 amino acid sequences. Highlighted areas designate residues with shared biochemical characteristics, as designated by the ClustalW [33] output, where black shading = identical residues; dark shading = strongly similar residues; light shading = weakly similar residues. Also noted are the residues composing the N- and C-termini and the transmembrane (TM) domains I–VII.

The deduced amino acid sequences of *Aa*DOP1 and *Aa*DOP2 were analyzed to identify conserved structural features typically associated with biogenic amine-binding GPCRs (Table S2), as well as unique regions that could be potentially exploited for development of mosquito-specific chemistries. Conserved features included sites predicted for ligand binding, protein stability, and G protein-coupling, and residues with potential for post-translational modification were identified. Alignments of the full-length *Aa*DOP1 and *Aa*DOP2 amino acid sequences (Figure 3) indicated that these sequences were divergent in the presumed N- and C-termini and the intracellular and extracellular loops, and the TM domains were moderately conserved (47% amino acid identity). A substantial difference was observed in the composition and relative size of the third intracellular loop that was much larger in *Aa*DOP2 (115 amino acids) than in *Aa*DOP1 (62 amino acids). Only a modest level of similarity was observed between the mosquito and human D_1_-like dopamine receptors, which shared between 47–54% amino acid identities among the TM domains, which typically represent the most conserved regions of GPCRs (Table S3). Moreover, comparison of the predicted TM domains from multiple invertebrate and vertebrate D_1_-like dopamine receptors showed that only 34% (58/172) of the amino acids were shared among all species included in the alignment (Figure S3). The highest level of sequence similarity to the TM domains of *Aa*DOP1 and *Aa*DOP2 was found in their predicted *D. melanogaster* orthologs, D-Dop1 (88% identity) (Table S3) and DopR99B (97% identity), respectively.

### Heterologous expression and pharmacological characterization

To study the function of the putative dopamine receptors *Aa*DOP1 and *Aa*DOP2, each receptor was expressed in HEK293 cells. Production of the mosquito receptor transcripts in transiently-transfected cells was first verified using RT-PCR (Figure S4). Increases of intracellular cAMP were detected in cells transiently expressing either *Aa*DOP1 [2.7±0.6 fold (n = 3)] or *Aa*DOP2 [48±14 fold (n = 3)] in response to a single dose of dopamine (10 µM) (Figure S5). No significant increase in cAMP was observed in the mock transfected cells (empty pcDNA3.1+ vector). For cells transiently expressing *Aa*DOP1, relatively high levels of constitutive activity were observed (17.6±2.4 fold greater than in mock transfected cells) as compared to *Aa*DOP2 (1.83±0.93 fold greater than in mock transfected cells).

**Figure 4:**
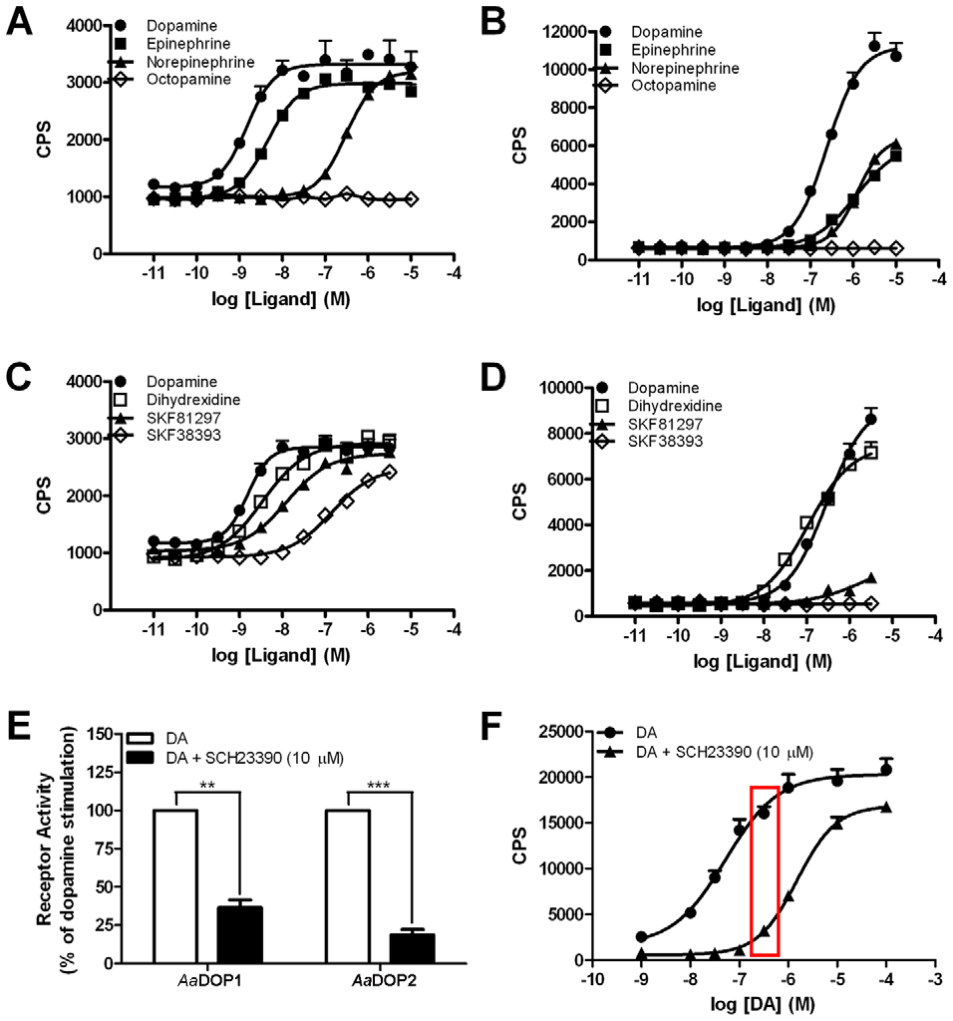
Pharmacological characterization of the *Aedes aegypti Aa*DOP1 and *Aa*DOP2 receptors. The mosquito receptors were stably expressed in HEK 293-CRELuc cells for dose-response assays and determination of EC_50_ values (shown in Table 1). **A, C**: *Aa*DOP1, **B, D**: *Aa*DOP2. Representative curves for **A, B**: biogenic amines; **C, D**: synthetic dopamine receptor agonists; **E**: Inhibitory effect of 10 µM SCH23390 in the presence of 1 µM dopamine (n = 4) shown for both mosquito dopamine receptors. ** *p*<0.01; *** *p*<0.001; **F**: Dose-response curve of dopamine for *Aa*DOP2 in the absence or presence of 10 µM SCH23390 used to identify an appropriate “signal window” for chemical library screening. The concentration of dopamine selected for screening (300 nM) is indicated with a box. CPS = counts per second; M = molarity.

Subsequently, dose-response curves for seven different biogenic amines were generated using HEK-CRELuc cells stably expressing either *Aa*DOP1 or *Aa*DOP2 (Figure 4; Table 1). Again, dopamine stimulated both receptors, with EC_50_ values determined at 3.1±1.1 nM and 240±16.0 nM for *Aa*DOP1 and *Aa*DOP2, respectively (Figure 4A–B; Table 1). In addition, we observed activation of the *Aa*DOP1 receptor by epinephrine (EC_50_ = 5.8±1.5 nM) and norepinephrine (EC_50_ = 760±180 nM) (Table 1). Conversely, no significant stimulation was observed for the *Aa*DOP2 receptor by epinephrine or norepinephrine (Table 1). Neither receptor was stimulated by histamine, octopamine, serotonin, or tyramine (EC_50_≥10 uM). The effects of known synthetic dopamine receptor agonists were also investigated (Figure 4C–D; Table 1). Considerable stimulation was observed for *Aa*DOP1 with the agonists listed in their rank order of potency: DHX>SKF81297>SKF38393. In contrast, of the synthetic agonists tested here, only treatment with DHX resulted in significant dose-dependent activation of *Aa*DOP2. The addition of the D_1_ dopamine receptor antagonist SCH23390 (10 µM) robustly inhibited the dopamine-mediated stimulation of both *Aa*DOP1 and *Aa*DOP2 (Figure 4E).

**Table 1:** Responses of *Aa*DOP1 and *Aa*DOP2 to biogenic amines and synthetic dopamine receptor agonists.

Compound	EC_50_ values	
	*Aa*DOP1	*Aa*DOP2
Dopamine	3.1±1.1 nM	240±16 nM
Epinephrine	5.8±1.5 nM	≥10 µM
Norepinephrine	760±180 nM	≥10 µM
Histamine	≥10 µM	≥10 µM
Octopamine	≥10 µM	≥10 µM
Serotonin	≥10 µM	≥10 µM
Tyramine	≥10 µM	≥10 µM
Dihydrexidine	6.9±1.5 nM	290±54 nM
SKF 81297	24±7.0 nM	≥10 µM
SKF 38393	310±46 nM	≥10 µM

HEK293 cells stably expressing both a CRELuc reporter construct and either of the receptors were stimulated with potential agonists. Dose-response curves were plotted and the EC_50_ values were calculated. Compounds with EC_50_ values ≥10 µM are considered to lack intrinsic activity at *Aa*DOP2.

### Screening of *Aa*dop2 against the LOPAC_1280_ library

We selected the *Aa*DOP2 receptor for an antagonist screen of the LOPAC_1280_ library because of its low constitutive activity and strong dopamine response compared to background (approximately 10-fold) (Figure 4B,D). Using dose-response studies, it was determined that 300 nM dopamine alone and in combination with 10 µM SCH23390 created a suitable “signal window” for identification of *Aa*DOP2 antagonists (Figure 4F). A “checkerboard analysis” using these conditions and assuming four replicates in the screen generated a Z-factor of 0.5±0.1 (n = 3), indicating that the assay was suitable for antagonist screening.

The criterion for “hit” detection was established relative to the control antagonist (SCH23390 response +3 standard deviations), such that only those compounds that inhibited the dopamine response by at least 81% were considered (Table 2). Based on this, our screen identified 51 potential antagonists of the *Aa*DOP2 receptor (complete screen results provided in Table S4). These compounds were partitioned into seven different classes based on their known biochemical interactions with mammalian molecular targets that included dopamine receptor antagonists (20), serotonin (6), histamine (2), and acetylcholine receptor ligands (1), biogenic amine uptake inhibitors (9), protein kinase modulators (6), and miscellaneous chemistries such as cell cycle regulators and apoptosis inhibitors (7).

**Table 2:** Summary of antagonistic hits identified from the *Aa*DOP2 screen against the LOPAC_1280_ library.

*Aa*DOP2 hit class	Chemistry	% of the SCH23390 effect†	Mode of action
Dopamine receptor antagonists (20)	R(+)-SCH-23390 hydrochloride* ‡	83	D_1_ DAR antagonist
	(±)-Butaclamol hydrochloride	81	D_2_ DAR selective antagonist
	(+)-Butaclamol hydrochloride‡	87	DAR antagonist
	Chlorprothixene hydrochloride	94	D_2_ DAR antagonist
	Clozapine‡	81	D_4_ DAR selective antagonist
	Fluphenazine dihydrochloride	82	DAR antagonist
	cis-(Z)-Flupenthixol dihydrochloride‡	88	DAR antagonist
	JL-18	98	D_4_ DAR selective antagonist
	LE 300	99	D_1_ DAR antagonist
	Loxapine succinate	97	N.D.
	(±)-Octoclothepin maleate	97	D_2_DAR/5-HT receptor antagonist
	Perphenazine	95	D_2_ DAR antagonist, σ receptor agonist
	Prochlorperazine dimaleate	83	DAR antagonist
	Promazine hydrochloride	88	D_2_ DAR antagonist
	Propionylpromazine hydrochloride	85	D_2_ DAR antagonist
	Risperidone	83	D_2_ DAR/5-HT receptor antagonist
	Triflupromazine hydrochloride	88	D_2_ DAR antagonist
	Trifluoperazine dihydrochloride	81	DAR/calmodulin antagonist
	Thiothixene hydrochloride	86	DAR antagonist
	Thioridazine hydrochloride	86	DAR/Ca^2+^ channel antagonist
Serotonin receptor ligands (6)	Amperozide hydrochloride	83	5-HT & DAR antagonist
	LY-310,762 hydrochloride	81	5-HT_1D_ selective antagonist
	Mianserin hydrochloride‡	95	5-HT receptor antagonist
	Methiothepin mesylate‡	99	5-HT_1_ selective antagonist
	Pirenperone	90	5-HT_2_ selective antagonist
	Ritanserin	83	5-HT_2_ selective antagonist
Histamine receptor ligands (2)	Ketotifen fumarate	96	H1 antagonist
	Promethazine hydrochloride	95	H1 antagonist
mAChR ligands (1)	Benztropine mesylate	89	mAChR antagonist
Biogenic amine uptake inhibitors (9)	Amitriptyline hydrochloride‡	90	N.D.
	Amoxapine	90	NOR uptake inhibitor
	4′-Chloro-3-alpha-(diphenylmethoxy) tropane hydrochloride	85	DA uptake inhibitor
	Doxepin hydrochloride‡	90	N.D.
	Imipramine hydrochloride	96	5-HT & NOR uptake inhibitor
	Maprotiline hydrochloride	82	NOR uptake inhibitor
	Nortriptyline hydrochloride	96	N.D.
	Protriptyline hydrochloride	82	NOR uptake inhibitor
	Trimipramine maleate	87	5-HT & NOR uptake inhibitor
Protein kinase modulators (6)	Diacylglycerol kinase inhibitor I	90	Diacylglycerol kinase inhibitor
	Kenpaullone	83	Phosphatase inhibitor
	NSC 95397	83	Syk, Lck inhibitor
	Piceatannol‡	98	CDK inhibitor
	Phorbol 12-myristate 13-acetate	88	Activates protein kinase C
	Purvalanol A	93	CDK1, CDK2, CDK5 inhibitor
Miscellaneous; e.g., cell cycle regulators/apoptosis modulators (7)	beta-Lapachone	86	Induces apoptosis
	(S)-(+)-Camptothecin	93	DNA topoisomerase I inhibitor
	Emetine dihydrochloride hydrate	86	Apoptosis inducer; RNA-protein translation inhibitor
	Idarubicin	83	Disrupts topoisomerase II
	Mitoxantrone	83	DNA synthesis inhibitor
	Niclosamide‡	95	Uncouples oxidative phosphorylation
	Resveratrol‡	89	Inhibits lipo- & cyclo-oxygenase activity
**Total**	**51 (4% hit rate)**		

**†:** Percent inhibition of receptor response in the presence of test compound relative to the SCH23390 control;

*, SCH23390 “antagonist control”;

**‡:** , compound analyzed in cAMP confirmation assay; CDK, cyclin dependent kinase; DAR, dopamine receptor; H, histamine receptor; Lck, lymphocyte-specific protein tyrosine kinase; NOR, norepinephrine; mAChR, muscarinic acetylcholine receptor; Syk, spleen tyrosine kinase; σ, sigma receptor; 5-HT, 5-hydroxytryptamine (serotonin). N.D. = not determined.

**Figure 5:**
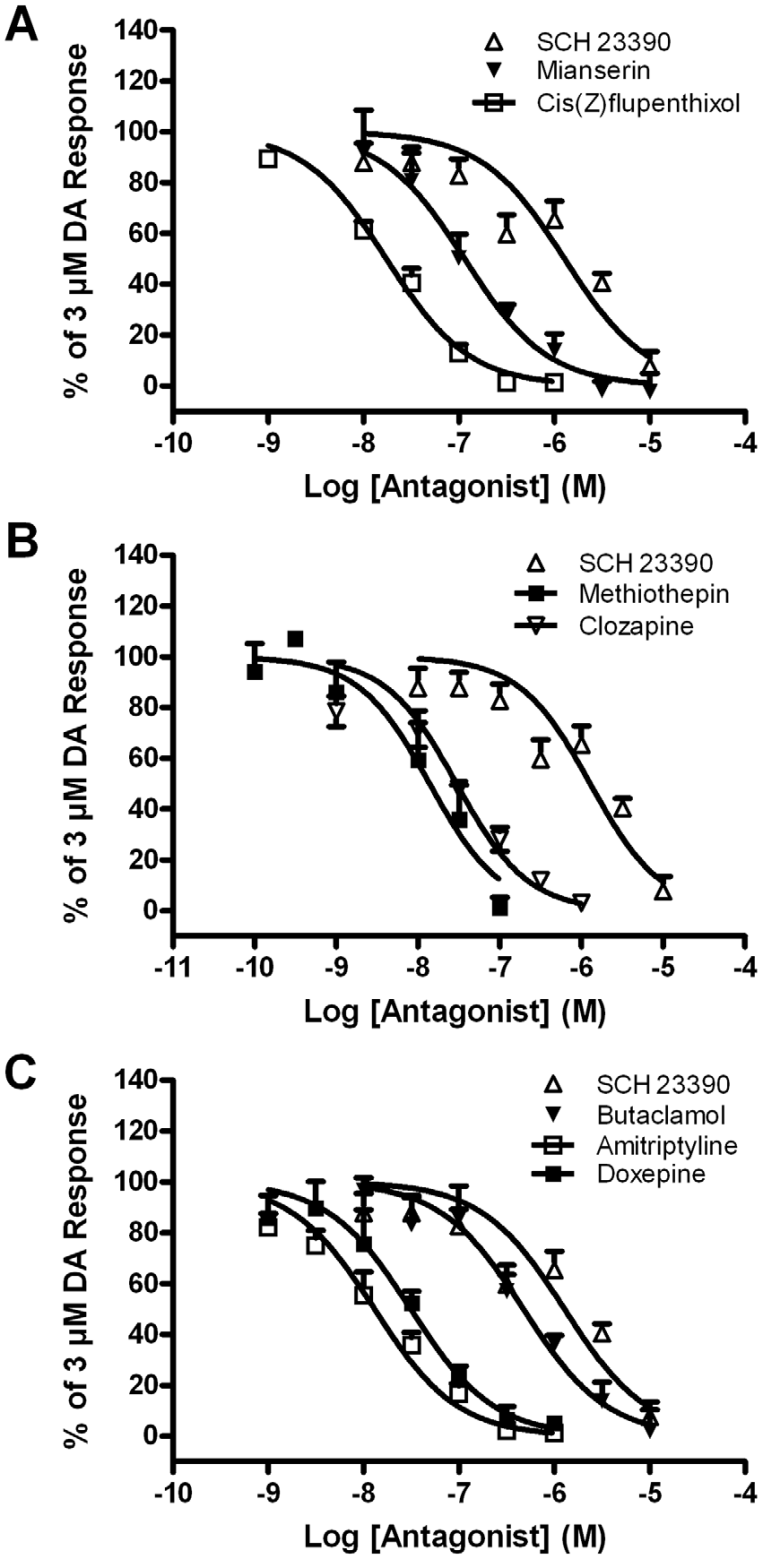
Dose-response curves for selected screen “hit” compounds that exhibited antagonistic effects on *Aa*DOP2. Direct cAMP accumulation assays were used for dose-response assays and determination of IC_50_ values for SCH23390 (antagonist control) and seven *Aa*DOP2 antagonists (shown in Table 3) identified in the chemical library screen.

Ten “hit” compounds (amitriptyline hydrochloride, (±)-butaclamol hydrochloride, clozapine, doxepin hydrochloride, cis-(Z)-flupenthixol dihydrochloride, methiothepin maleate, mianserin hydrochloride, niclosamide, piceatannol, and resveratrol), in addition to SCH23390 were selected for screen validation assays. These compounds were tested for their activity in cAMP accumulation assays to control for potential “off-target” effects (i.e. chemistries that affect the CRELuc reporter system). Seven of these compounds were potent antagonists of the *Aa*DOP2 receptor, as shown by the dose-dependent reduction of cAMP accumulation relative to the dopamine-stimulated control (Table 3, Figure 5). Three of the compounds (i.e. niclosamide, piceatannol, and resveratrol) showed no significant antagonistic effects against *Aa*DOP2 in the cAMP accumulation experiments, having IC_50_ values ≥10 µM.

**Table 3:** Confirmation and secondary assays for “hit” antagonists of *Aa*DOP2 and human D_1_ receptor.

Compound	IC_50_ value (at 3 µM dopamine for *Aa*DOP2)	IC_50_ value (at 100 nM dopamine for hD_1_)	Relative fold selectivity for *Aa*DOP2 vs. hD_1_
Amitriptyline	14±3.4 nM	470±49 nM	36
(+) Butaclamol	480±33 nM	3.7±0.64 nM	0.008
cis-(Z)-Flupenthixol	20±5.4 nM	11±1.9 nM	0.55
Clozapine	31±6.5 nM	300±35 nM	9.7
Doxepin	31±4.9 nM	960±86 nM	31
Methiothepin	14±5.1 nM	80±11 nM	5.7
Mianserin	120±40 nM	1200±260 nM	10
Niclosamide	≥10 µM	N.D.	N.D.
Piceatannol	≥10 µM	N.D.	N.D.
Resveratrol	≥10 µM	N.D.	N.D.
SCH23390	1600±73 nM	0.47±0.03 nM	0.0003

Select chemistries and the assay control (SCH23390) were tested in dose-response cAMP assays in the presence of 3 µM dopamine in *Aa*DOP2- or 100 nM dopamine in hD_1_-expressing cells (Figure 5). Compounds with IC_50_ values ≥10 µM are considered to lack activity at *Aa*DOP2 and were not tested at hD_1_. N.D. = not determined; hD_1_ = Human D_1_ dopamine receptor.

### “Hit-to-lead” studies

#### Confirmation and secondary *in vitro* assays

Selected hit compounds were also tested against the human D_1_ receptor (hD_1_) to allow for comparisons of relative potency between species (Table 3). These experiments clearly indicated a unique pharmacology of *Aa*DOP2 compared to hD_1_ with divergent rank order functional potencies that showed no significant correlation (R^2^<0.15). For example, the prototypical mammalian D_1_ antagonist, SCH23390 was greater than 3000-fold more selective for hD_1_ than *Aa*DOP2. In contrast, the data also revealed that two structurally-related tricyclic antidepressants (i.e. amitriptyline and doxepin) had more than 30-fold selectivity for *Aa*DOP2 when compared to hD_1_. These observations suggest that the significant differences between these receptors could be exploited for the development of *Aa*DOP2-selective compounds.

### *In vivo Ae. aegypti* bioassays

**Figure 6:**
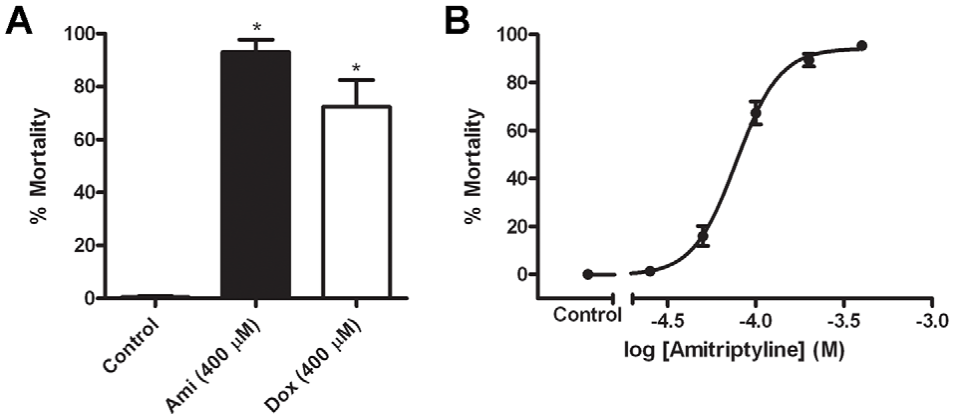
Toxicity of antagonist screen hits in *Ae. aegypti* larval bioassays. **A:** *Ae. aegypti* larval bioassay showing toxicity of amitriptyline and doxepin at a single dose point (400 µM) compared to the water control; Ami = amitriptyline, Dox = Doxepin; * indicates *p*<0.05; **B:** *Ae. aegypti* larval bioassay involving amitriptyline in a dose-response format (25 µM–400 µM).

The toxicity of the *Aa*DOP2 antagonist screen hits amitriptyline and doxepin was assessed in *Ae. aegypti* larval bioassays. These chemistries were selected due to their relatively higher potency at *Aa*DOP2 compared to hD_1_ (Table 3). Single dose-point assays at 400 µM effective concentration of drug revealed that amitriptyline (93% average mortality) and doxepin (72% average mortality) each caused significant mortality (*p*<0.05) 24 hours post-treatment relative to the water control (0% mortality) (Figure 6A), whereas no mortality was observed for SCH23390 during this timeframe (data not shown). In addition, dose-response experiments were conducted for amitriptyline, which caused a rapid and high mortality effect in the single-point assays. The toxicity of amitriptyline was dose-dependent, and the LC_50_ and LC_90_ values were determined at 78 µM and 185 µM, respectively (Figure 6B).

## Discussion

This work provides the first detailed investigation into the molecular and pharmacological properties of D_1_-like dopamine receptors, *Aa*DOP1 and *Aa*DOP2, from the mosquito vector of dengue and yellow fever, *Ae. aegypti*, and the development of a cell-based screen assay to discover antagonists of *Aa*DOP2. Our study employed a novel pipeline utilizing a “genome-to-lead” approach for the discovery of new chemistries for vector control. This research establishes a basis for improving understanding of mosquito dopaminergic processes *in vivo* and for chemical screening of these and other receptors characterized in arthropod vectors of human disease, such as in the Lyme disease tick, *I. scapularis* [36], [42]. To our knowledge, Lee and Pietrantonio [47] have published the only other study involving the functional characterization of a biogenic amine-binding GPCR in mosquitoes that was focused on a Gα_s_-coupled serotonin receptor in *Ae. aegypti*. Furthermore, ligands of only four other cloned GPCRs have been pharmacologically verified in mosquitoes, including those that target an adipokinetic hormone receptor, a corazonin receptor, a crustacean cardioactive peptide receptor [48], and an adipokinetic/corazonin-related peptide receptor in the malaria mosquito, *A. gambiae* [49].

Typically, insects possess three different dopamine receptors including two D_1_-like receptors and a single D_2_-like receptor [43]. Here, RT-PCR data were used to validate the two mosquito D_1_-like dopamine receptor gene models [10]; this enabled confirmation of intron/exon boundaries and prediction of the complete protein coding regions needed prior to heterologous expression studies. A putative D_2_-like dopamine receptor gene (*Aa*DOP3) was also identified in *Ae. aegypti* [10] although this receptor has not yet been functionally characterized. The RT-PCR studies also demonstrated that transcripts for both D_1_-like dopamine receptor genes were detectable in each developmental stage of *Ae. aegypti*, suggesting the importance of these receptors throughout the mosquito life cycle. Much progress has been made in determining the life-stage and tissue-specific expression dynamics of the orthologous dopamine receptors in *D. melanogaster* [14], [30], [31], [40], [44], [50], *A. mellifera* [41], [43], , and most recently in the Lyme disease tick, *I. scapularis* [42]. Our research will support future complementary studies needed to localize expression of these dopamine receptors in mosquito tissues to gain further insight toward their neurophysiological roles.

The *Aa*DOP1 and *Aa*DOP2 amino acid sequences were compared and analyzed to identify conserved as well as unique features of the receptors. Several characteristics typically associated with biogenic amine-binding GPCRs were evident, including aspartate residues in TM II and TM III that are thought to interact with the amine moieties of catecholamines [55]. The conserved serine residues in TM V and aromatic residues in TM V and VI are also potentially important for ligand interaction [56], [57]. In both receptors, the conceptual cytoplasmic region of TM III contained the conserved “DRY” motif associated with G protein-coupling [58], [59], and a pair of cysteine residues were located in the extracellular loops I and II that may form a disulfide bond for protein stabilization [58], [60], [61]. Interestingly, the divergent intracellular loop III was predicted to be almost twice as long in *Aa*DOP2 (115 amino acids) than in *Aa*DOP1 (62 amino acids), but the sizes of the carboxyl tail region were similar between these receptors. This corresponded well with the relative sizes of these features in the fruit fly and honeybee orthologs [43]; however, the significance of these characteristics is yet to be determined in the mosquito. Importantly, the *Aa*DOP1 and *Aa*DOP2 sequences were markedly different from the human D_1_-like dopamine receptor sequences. Although a modest level of amino acid identity (∼50%) was observed between the TM domains, the N- and C-termini and extracellular and intracellular loop regions were highly divergent (data not shown). These differences suggest that there exists potential for identifying chemistries that are mosquito-specific and, importantly, do not interfere with dopaminergic functioning in humans.

Heterologous expression experiments conducted in HEK293 cells provided experimental evidence that the *Ae. aegypti* receptors are functional D_1_-like dopamine receptors. We measured significant increases in cAMP accumulation following dopamine treatment of cells transiently expressing either *Aa*DOP1 or *Aa*DOP2, suggesting that both receptors couple to Gα_s_ proteins. This effect was further substantiated in cell lines stably co-expressing either of these receptors and the CRELuc reporter system, as measured by an increase in luciferase activity following dopamine treatment. Future research is needed to determine if these receptors operate through multiple cellular signaling mechanisms, such as was shown for the *D. melanogaster* dopamine receptor involved with both cAMP and calcium signaling [62].

The stably transformed cell lines were used to compare the pharmacological properties of *Aa*DOP1 and *Aa*DOP2 in response to seven different biogenic amines. For dopamine, we measured EC_50_ values in the nanomolar range for both *Aa*DOP1 (3.1±1.1 nM) and *Aa*DOP2 (240±16 nM). However, there were differences in the responses of these receptors to the other biogenic amines. *Aa*DOP2 was activated only with dopamine, whereas *Aa*DOP1 was stimulated by dopamine, epinephrine, and to a lesser extent, norepinephrine. These results were similar to those reported for the orthologous dopamine receptors in the tick *I. scapularis* [36], [42]. Another difference between *Aa*DOP1 and *Aa*DOP2 was observed regarding constitutive activity. In both transient and stable expression experiments, the *Aa*DOP1 receptor exhibited significant constitutive activity, as determined by the elevated levels of cAMP detected in the absence of a receptor agonist, whereas *Aa*DOP2 did not. Such constitutive activity was also reported for the D_1_-like dopamine receptors *Am*DOP1 of *A. mellifera* [41], *Ce*DOP1 from the nematode *Caenorhabditis elegans* [63], *Is*dop1 of *I. scapularis* [36], and the human D_5_ receptor [64]. Seifert and Wenzel-Seifert [65] proposed that constitutive activity of a GPCR may enable the maintenance of basal neuronal activity, although evidence is needed to support such activity for *Aa*DOP1 *in vivo*.

The pharmacological properties of *Aa*DOP1 and *Aa*DOP2 were further explored by testing their responses to synthetic dopamine receptor agonists and antagonists. Both receptors were strongly stimulated by the agonist DHX; however, only *Aa*DOP1 significantly responded to the well characterized D_1_ agonists SKF81297 and SKF38393. This differential response to the SKF compounds was also observed for the orthologous D_1_-like dopamine receptors in the tick *I. scapularis* [36]. Interestingly, neither of the *D. melanogaster* D_1_-like dopamine receptors was strongly stimulated by SKF38393 [31], [40]. Both *Aa*DOP1 and *Aa*DOP2 were inhibited by the antagonist SCH23390, as were the tick D_1_-like receptors [36]. This contrasted with the lack of significant inhibition reported by SCH23390 for D-dop1 in the fruit fly [40] and DOP1 of the honeybee [51]. Given the limited number of drugs that have been tested against these receptors, to date, these differential pharmacological responses provide further evidence that it may be possible to discover chemistries that operate specifically at the mosquito dopamine receptors.

Our over-arching goal was to develop a pipeline to identify lead chemistries active at biogenic-amine binding GPCRs in vector arthropods. Broadly speaking, we define a lead chemistry as any molecule, or its analog or derivative, with potential for insecticide development. In our study, this refers to any molecule identified by screening and subsequently confirmed in a variety of “hit-to-lead” assays. The LOPAC_1280_ library was chosen for our pilot screen because it is enriched with chemistries that influence dopaminergic processes and includes other GPCR-binding ligands. We hypothesized that chemistries that antagonize these dopamine receptors may possess insecticidal properties. Precedent for this concept stems from pest management successes associated with the use of phenylpyrazoles (e.g. Fipronil) and cyclodienes, which block GABA-gated chloride channels and have highly insecticidal properties [66], [67]. This notion was pursued using HEK293 cells stably expressing *Aa*DOP2 because this receptor has a robust response to dopamine and a low constitutive activity, which are properties that aid interpretation of screen data. Our initial screen was directed at the identification of *Aa*DOP2 antagonists; the success of this experiment justifies expanded screening to explore the antagonist chemical “space”, and with assay modification, screens to detect agonists active at this receptor. Moreover, development of the *Aa*DOP1 assay would enable comparative screens against LOPAC_1280_ chemistries.

Of the 51 hit *Aa*DOP2 antagonists identified in the LOPAC_1280_ library, 20 (39%) are known antagonists of mammalian dopamine receptors. A majority of these chemistries fall into the benzodiazepine, phenothiazine, or thioxanthene classes that in other systems are known to bind other biogenic amine receptors. Included were ligands selective for D_1_- and D_2_-like dopamine receptors in mammalian systems, as well as several non-dopamine receptor selective compounds such as (±)-butaclamol, cis-(Z)-flupenthixol, and the atypical antipsychotic, clozapine. These three compounds were tested in a dose-response format for their ability to inhibit dopamine-stimulated cAMP accumulation. The IC_50_ values demonstrated the following rank order of potency clozapine>cis-flupenthixol>butaclamol. The next largest grouping of identified compounds includes inhibitors of the biogenic amine transporters (9 compounds, 18%). Several serotonin receptor antagonists (6 compounds, 12%) were identified as well. Follow-up dose response studies with selected chemistries from the identified transport inhibitors and serotonin antagonists (i.e. methiothepin, mianserin, amitriptyiline, and doxepin) revealed that these compounds were potent antagonists at the *Aa*DOP2 receptor and were much more potent than the prototypical D_1_ antagonist, SCH23390 (Table 3). The antagonistic activity of these ligands is not completely surprising; the National Institute of Mental Health's Psychoactive Drug Screening Program (NIMH-PDSP) database reports K_i_ values for the human D_1_-like dopamine receptors at 80–900 nM (http://pdsp.med.unc.edu/). However, these observations, combined with the dopamine antagonist screen results, indicate that well studied and clinically used compounds could be used to target invertebrate GPCRs. In fact, a number of the chemistries identified in our screen have been used in humans for decades, suggesting the possibility of “drug repurposing” as insecticides. Further precedent for the concept of insect-specific chemistries can be drawn from the fact that a number of insecticides (e.g., pyrethroids and fipronil) are considerably more selective at invertebrate as opposed to mammalian targets [68]. The screen also identified multiple protein kinase modulators and several agents that regulate germane cellular functions that presumably inhibit the CRE response via non-*Aa*DOP2 mechanisms. Support for this hypothesis was demonstrated in the direct measurement of cAMP accumulation experiments, where resveratrol, pieacetannol, and niclosamide each lacked activity. The remaining three “hit” compound classes included antagonists of either histamine or muscarinic acetylcholine receptors, and this likely reflects the lack of receptor selectivity for these ligands.

The LOPAC_1280_ library includes several known antagonists of mammalian dopamine receptors that did not qualify as hits in our screen. In part, this can be explained by the fact that we used a highly stringent cut-off to signify antagonistic activity at *Aa*DOP2. Had we reduced the stringency to select for hits with an antagonistic effect equivalent to that of SCH23390+6 standard deviations (69% inhibition), our screen would have returned an additional 13 hit chemistries, including compounds predicted to have a modest antagonistic effect at *Aa*DOP2 and those that are more selective for D_2_-like dopamine receptors. Considering the substantial divergence between the mosquito and human D_1_-like dopamine receptor sequences, there is a strong possibility that a subset of the “non-hit” dopamine receptor antagonists are not active at the mosquito receptor. In support of this, the prototypical mammalian D_1_ antagonist, SCH23390, was greater than 3000-fold more selective for hD_1_ than *Aa*DOP2. Although our comparison data set is limited to only eight compounds, these experiments suggest a very divergent pharmacology between these human and mosquito dopamine receptors. Thus, our study provides a foundation for subsequent comparative pharmacological analyses of the mosquito and human dopamine receptors.

Analyses involving a small subset of compounds revealed a correlation between our *in vitro* and *in vivo* data. The *Aa*DOP2 antagonist screen hits, amitriptyline and doxepin, caused significant lethality in the mosquito bioassay. Our finding that these drugs each have a relatively higher potency at the mosquito dopamine receptor than at the human dopamine receptor (hD_1_) has implications for the identification of arthropod-selective chemistries. Drugs with minimal or no impact on the neurological functioning of humans or other vertebrate species are particularly desirable as prospects for insecticide development. Conversely, SCH23390, which is active at *Aa*DOP2 only in the micromolar range and was several fold more selective for hD_1_ in cAMP assays, did not cause significant mortality at 24 hr.

The success of this initial chemical library screen in identifying new mosquitocidal chemical leads justifies the pursuit of an expanded high-throughput screening effort involving thousands or hundreds of thousands of chemistries against mosquito dopamine receptors. Our platform is also amenable for the screening of agonist chemistries active at these mosquito dopamine receptors, as well as for Gα_s_-coupled biogenic amine targets of other vector arthropods, and also could be modified to screen Gα_i/o_-coupled receptors [69]. Importantly, the identification of lead *Aa*DOP2 receptor antagonistic chemistries provides a basis for investigating the effect of these or related compounds on mosquito dopaminergic processes *in vivo* [70]. Follow-up research is needed to determine the precise mechanism(s) of amitriptyline- and doxepin-induced mortality in *Ae. aegypti*. Further work is also needed to determine if these chemistries and associated derivatives or analogs identified by chemical screens possess the properties desired of an insecticide (e.g. bioavailability, *in vivo* potency/toxicity, suitable half-life, lack of effects on non-target organisms, suitability for synthesis and formulation). Molecular modeling of three dimensional GPCR structures and their binding capabilities, as reported for an adipokinetic hormone receptor in *A. gambiae* [71] and a tyramine receptor in the moth *Plodia interpunctella* [72], may facilitate *in silico* chemical screening [73] and ligand-receptor studies that permit the design or refinement of lead molecules active at mosquito GPCRs.

Historically, multiple neuroactive processes in arthropods have been exploited for pest control using insecticides such as chlorinated hydrocarbons, organophosphates, methylcarbamates, pyrethroids, amidines, and phenylpyrazoles [67]. Resistance involving each of these classes (the vast majority of which operate by affecting ion channels and neurotransmitters) has been documented. The development of new mode-of-action insecticides could improve our arsenal against mosquito populations that have developed resistance to existing chemical formulations [1]. We suggest that the two dopamine receptors characterized here, as well as other biogenic amine-binding GPCRs [74], [75], represent promising targets for new insecticide research, due to their presumably central roles in insect neurobiology. This “proof-of-concept” study sets the stage for target-specific approaches for vector control. Such efforts, in parallel with activities of organizations such as the Innovative Vector Control Consortium, may help to realize the goal of delivering new insecticides for reduction of vector-borne diseases [2].

## Supporting Information

Figure S1 **Gel electrophoresis for non-quantitative RT-PCR of** ***Aedes aegypti Aadop1*** **and** ***Aadop2*** **.** **A**: *Aadop1* amplified with primers *Aadop*_1F/1R (224 bp amplicon), **B**: *Aadop2* amplified with primers *Aadop2*_Full_F/R (1,425 bp amplicon). Transcripts were detected for both dopamine receptors in each developmental stage of the mosquito and both adult sexes. As expected, no amplification products were detected in the negative control, which contained identical reagents as the other reactions but lacked an RNA template. Abbreviations: (M) DNA size marker (HyperLadder I, Bioline USA Inc., Randolph, MA); (E) egg; (L) larva; (P) pupa; (AF) adult female; (AM) adult male; (C) negative control.
(TIF)

Figure S2 **Gene models for** ***Aedes aegypti Aadop1*** **and** ***Aadop2*** **.** **A**: *Aadop1*, **B**: *Aadop2*. Exons (E) are shown with gray bars, and introns with solid black lines. Numbers above the box/line indicate the size of exon/intron in base pairs (bp), respectively. The putative transmembrane domains (I–VII) are shown with black boxes along the exons. The gene structures of *Aadop1* and *Aadop2* include three and two introns, respectively, which is consistent with other characterized insect dopamine receptor genes that also contain introns [43], but is in contrast with the single exon gene structures reported for the two D_1_-like receptor genes in humans [39], [76] and the Lyme disease tick, *I. scapularis* [36]. The genomic supercontigs on which *Aadop1* and *Aadop2* reside have not yet been linked to chromosomal positions [10], so their relative genome organization cannot yet be compared with other insects. However, in *A. gambiae* the predicted orthologs of *Aadop1* and *Aadop2* are positioned on chromosome 2R (GPRDOP1: AGAP004613) and the X chromosome (GPRDOP2: AGAP000667) [9].
(TIF)

Figure S3 **Alignment of transmembrane domains of** ***Aedes aegypti Aa*** **DOP1 and** ***Aa*** **DOP2 and other D_1_-like receptors.** Aligned receptor amino acid sequences include each of the two D_1_-like receptors reported in *Drosophila melanogaster* (D-Dop1; DopR99B/DAMB) [30], [31], [40], [44], *Apis mellifera* (*Am*DOP1; *Am*DOP2) [41], *Ixodes scapularis* (*Is*dop1; *Is*dop2) [36], [42], and *Homo sapiens* (*Hs*D1, *Hs*D5) [39], [76]. Amino acids included in the alignment were related to the TM regions predicted for *D. melanogaster* [30], [31]. Shaded amino acids designate residues conserved among each of the aligned TM domain sequences.
(TIF)

Figure S4 **Expression of** ***Aedes aegypti Aadop1*** **and** ***Aadop2*** **in transiently-transfected HEK 293 cells.** Gel electrophoresis shows PCR and RT-PCR amplification of *Aedes aegypti* **A**: *Aadop1*and **B**: *Aadop2* using primers *Aadop*1_1F/2R (amplicon = 1058 bp) and *Aadop2*_FullF/FullR (1425 bp), respectively. Abbreviations: (M) DNA size marker (HyperLadder I, Bioline USA Inc., Randolph, MA); lanes under the heading “PCR” include controls for DNA contamination in the RNA preparation: (−) no DNA template; (+) **A**: DNA construct pcDNA3.1+/*Aadop1* and **B**: DNA construct pcDNA3.1+/*Aadop2*; (V) mRNA from cells transfected with empty vector pcDNA3.1; (C) mRNA from cells transfected with construct **A**: pcDNA3.1+/*Aadop1* and **B**: pcDNA3.1+/*Aadop2*. Lanes under the heading “RT-PCR” show mRNA transcript detection experiments; (−) no template mRNA; (+) mRNA from adult female *Ae. aegypti* (non-specific amplification products were eliminated with gel purification); (V) mRNA from cells transfected with empty vector pcDNA3.1; (C) mRNA from cells transfected with construct **A**: pcDNA3.1+/*Aadop1* and **B**: pcDNA3.1+/*Aadop2*.
(TIF)

Figure S5 **Response of** ***Aa*** **DOP1 and** ***Aa*** **DOP2 following dopamine treatment in transiently-transfected HEK cells.** Significant responses to dopamine were observed for both *Aa*DOP1 and *Aa*DOP2, relative to basal conditions (*p*<0.05).
(TIF)

Table S1 **Primer pairs and experimental conditions used in RT-PCR analysis of** ***Aadop1*** **and** ***Aadop2*** **transcripts.**
(DOC)

Table S2 **Summary of selected amino acid features of** ***Aedes aegypti Aa*** **DOP1 and** ***Aa*** **DOP2.**
(DOC)

Table S3 **Comparison of transmembrane domains of** ***A. aegypti Aa*** **DOP1 and** ***Aa*** **DOP2 and related D_1_-like receptors.**
(DOC)

Table S4 **Results of the** ***Aedes aegypti Aa*** **DOP2 antagonist screen of the LOPAC_1280_ library.**
(PDF)
