# Supplementary figures and images for: A “Genome-to-Lead” Approach for Insecticide Discovery: Pharmacological Characterization and Screening of Aedes aegypti D1-like Dopamine Receptors
Source: PLoS Negl Trop Dis. 2012 Jan 24;6(1):e1478. doi: 10.1371/journal.pntd.0001478 (PMC3265452; doi:10.1371/journal.pntd.0001478)

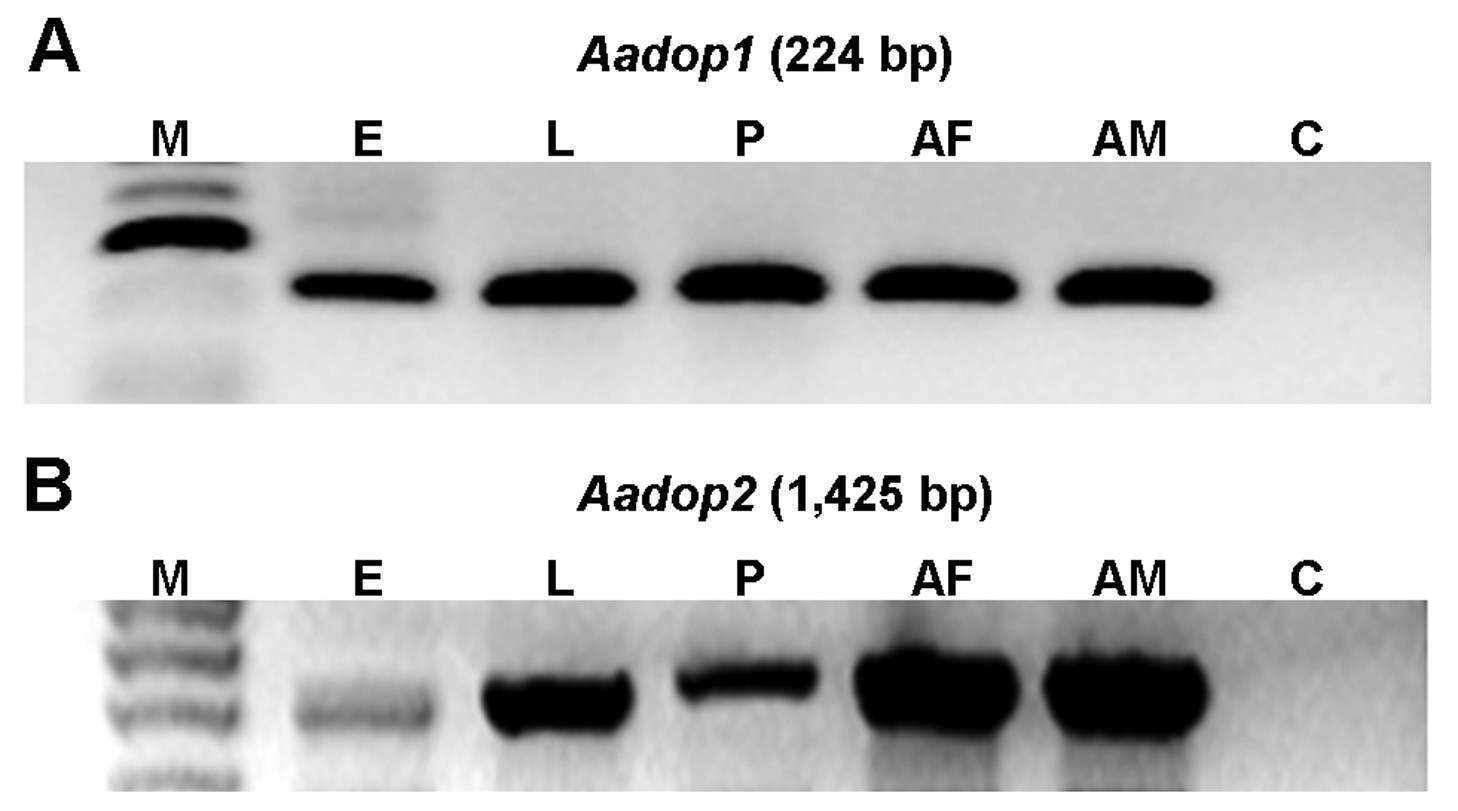

Supplement: Figure S1 — Gel electrophoresis for non-quantitative RT-PCR of Aedes aegypti Aadop1 and Aadop2 . A: Aadop1 amplified with primers Aadop_1F/1R (224 bp amplicon), B: Aadop2 amplified with primers Aadop2_Full_F/R (1,425 bp amplicon). Transcripts were detected for both dopamine receptors in each developmental stage of the mosquito and both adult sexes. As expected, no amplification products were detected in the negative control, which contained identical reagents as the other reactions but lacked an RNA template. Abbreviations: (M) DNA size marker (HyperLadder I, Bioline USA Inc., Randolph, MA); (E) egg; (L) larva; (P) pupa; (AF) adult female; (AM) adult male; (C) negative control. (TIF) [file pntd.0001478.s001.tif]

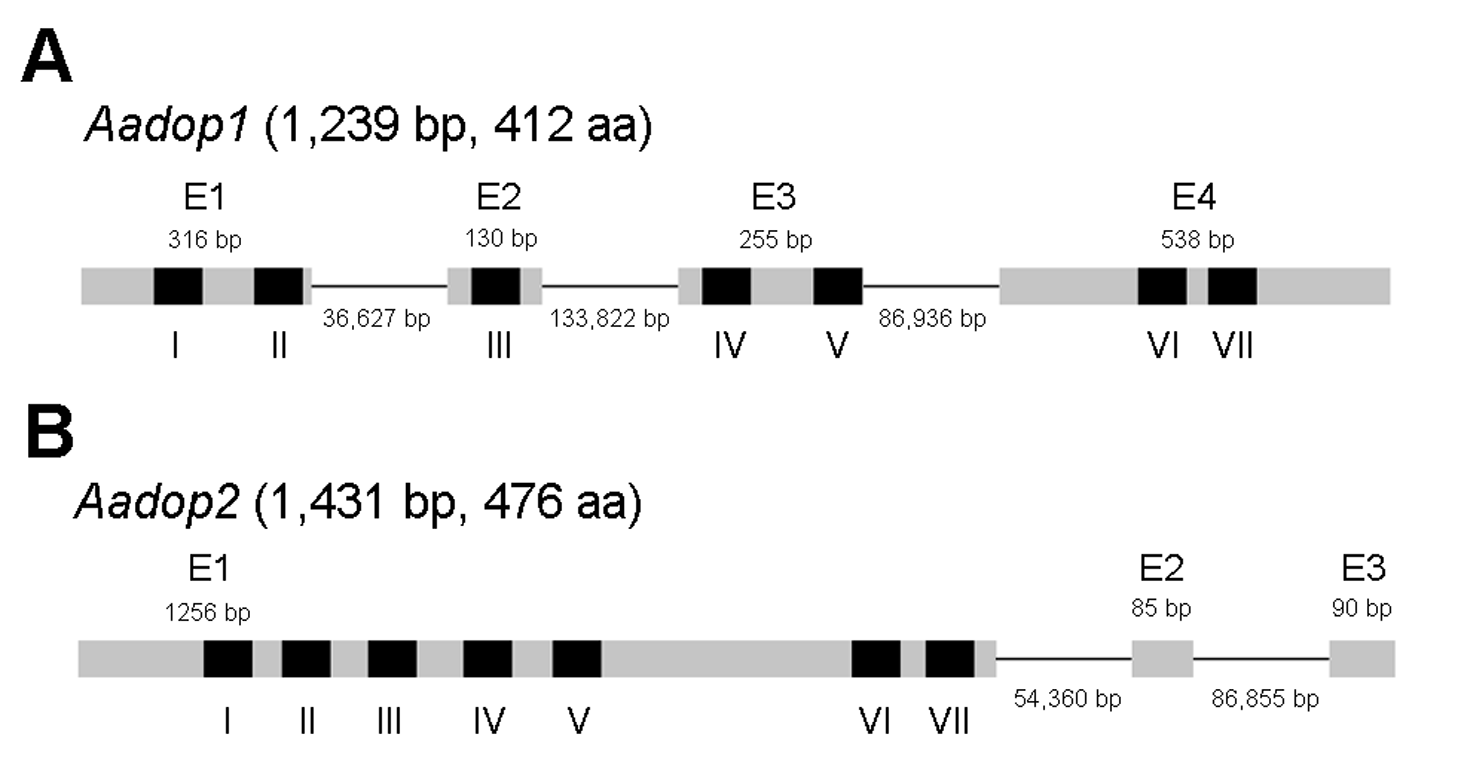

Supplement: Figure S2 — Gene models for Aedes aegypti Aadop1 and Aadop2 . A: Aadop1, B: Aadop2. Exons (E) are shown with gray bars, and introns with solid black lines. Numbers above the box/line indicate the size of exon/intron in base pairs (bp), respectively. The putative transmembrane domains (I–VII) are shown with black boxes along the exons. The gene structures of Aadop1 and Aadop2 include three and two introns, respectively, which is consistent with other characterized insect dopamine receptor genes that also contain introns [43], but is in contrast with the single exon gene structures reported for the two D1-like receptor genes in humans [39], [76] and the Lyme disease tick, I. scapularis [36]. The genomic supercontigs on which Aadop1 and Aadop2 reside have not yet been linked to chromosomal positions [10], so their relative genome organization cannot yet be compared with other insects. However, in A. gambiae the predicted orthologs of Aadop1 and Aadop2 are positioned on chromosome 2R (GPRDOP1: AGAP004613) and the X chromosome (GPRDOP2: AGAP000667) [9]. (TIF) [file pntd.0001478.s002.tif]

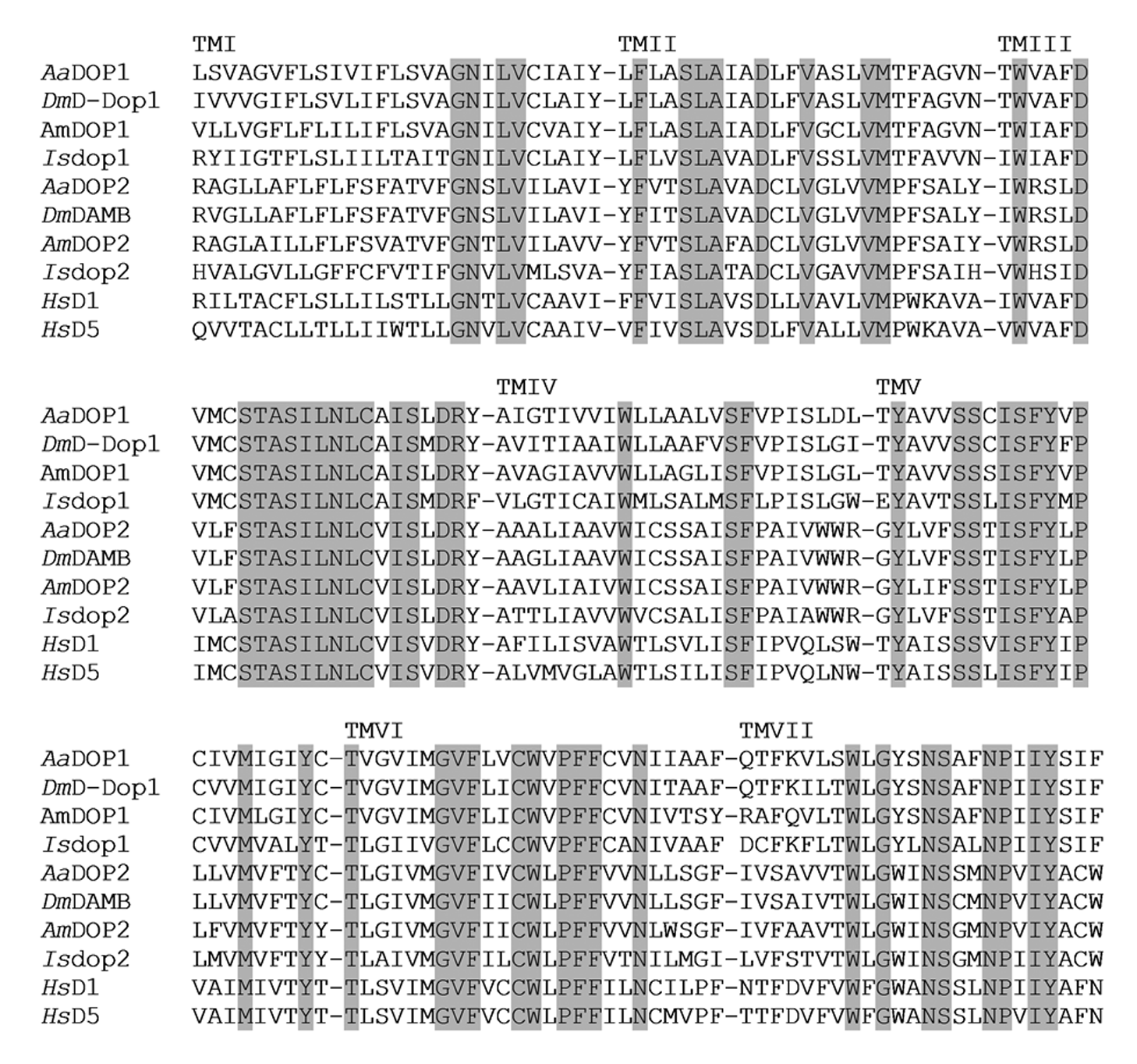

Supplement: Figure S3 — Alignment of transmembrane domains of Aedes aegypti Aa DOP1 and Aa DOP2 and other D1-like receptors. Aligned receptor amino acid sequences include each of the two D1-like receptors reported in Drosophila melanogaster (D-Dop1; DopR99B/DAMB) [30], [31], [40], [44], Apis mellifera (AmDOP1; AmDOP2) [41], Ixodes scapularis (Isdop1; Isdop2) [36], [42], and Homo sapiens (HsD1, HsD5) [39], [76]. Amino acids included in the alignment were related to the TM regions predicted for D. melanogaster [30], [31]. Shaded amino acids designate residues conserved among each of the aligned TM domain sequences. (TIF) [file pntd.0001478.s003.tif]

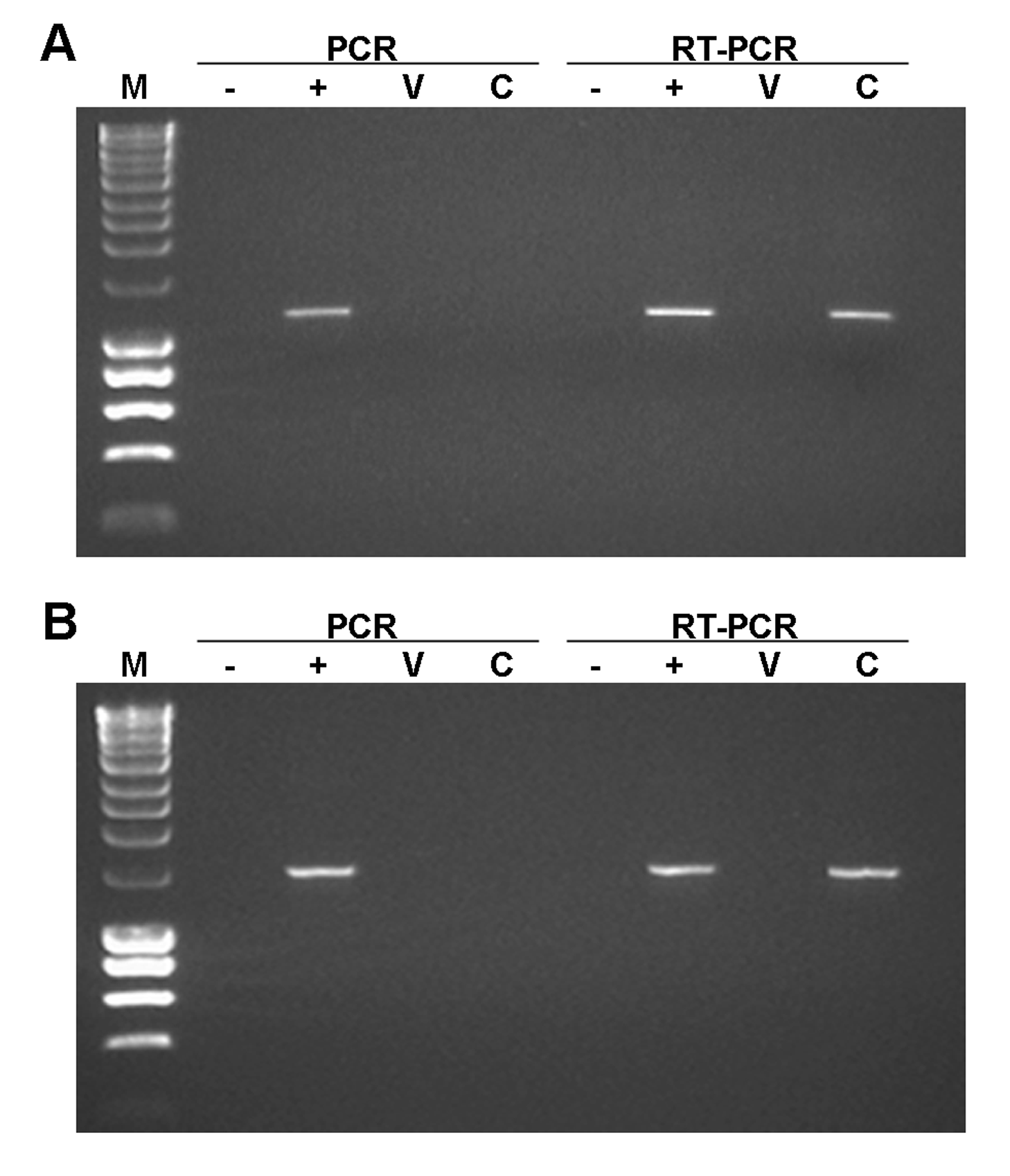

Supplement: Figure S4 — Expression of Aedes aegypti Aadop1 and Aadop2 in transiently-transfected HEK 293 cells. Gel electrophoresis shows PCR and RT-PCR amplification of Aedes aegypti A: Aadop1and B: Aadop2 using primers Aadop1_1F/2R (amplicon = 1058 bp) and Aadop2_FullF/FullR (1425 bp), respectively. Abbreviations: (M) DNA size marker (HyperLadder I, Bioline USA Inc., Randolph, MA); lanes under the heading “PCR” include controls for DNA contamination in the RNA preparation: (−) no DNA template; (+) A: DNA construct pcDNA3.1+/Aadop1 and B: DNA construct pcDNA3.1+/Aadop2; (V) mRNA from cells transfected with empty vector pcDNA3.1; (C) mRNA from cells transfected with construct A: pcDNA3.1+/Aadop1 and B: pcDNA3.1+/Aadop2. Lanes under the heading “RT-PCR” show mRNA transcript detection experiments; (−) no template mRNA; (+) mRNA from adult female Ae. aegypti (non-specific amplification products were eliminated with gel purification); (V) mRNA from cells transfected with empty vector pcDNA3.1; (C) mRNA from cells transfected with construct A: pcDNA3.1+/Aadop1 and B: pcDNA3.1+/Aadop2. (TIF) [file pntd.0001478.s004.tif]

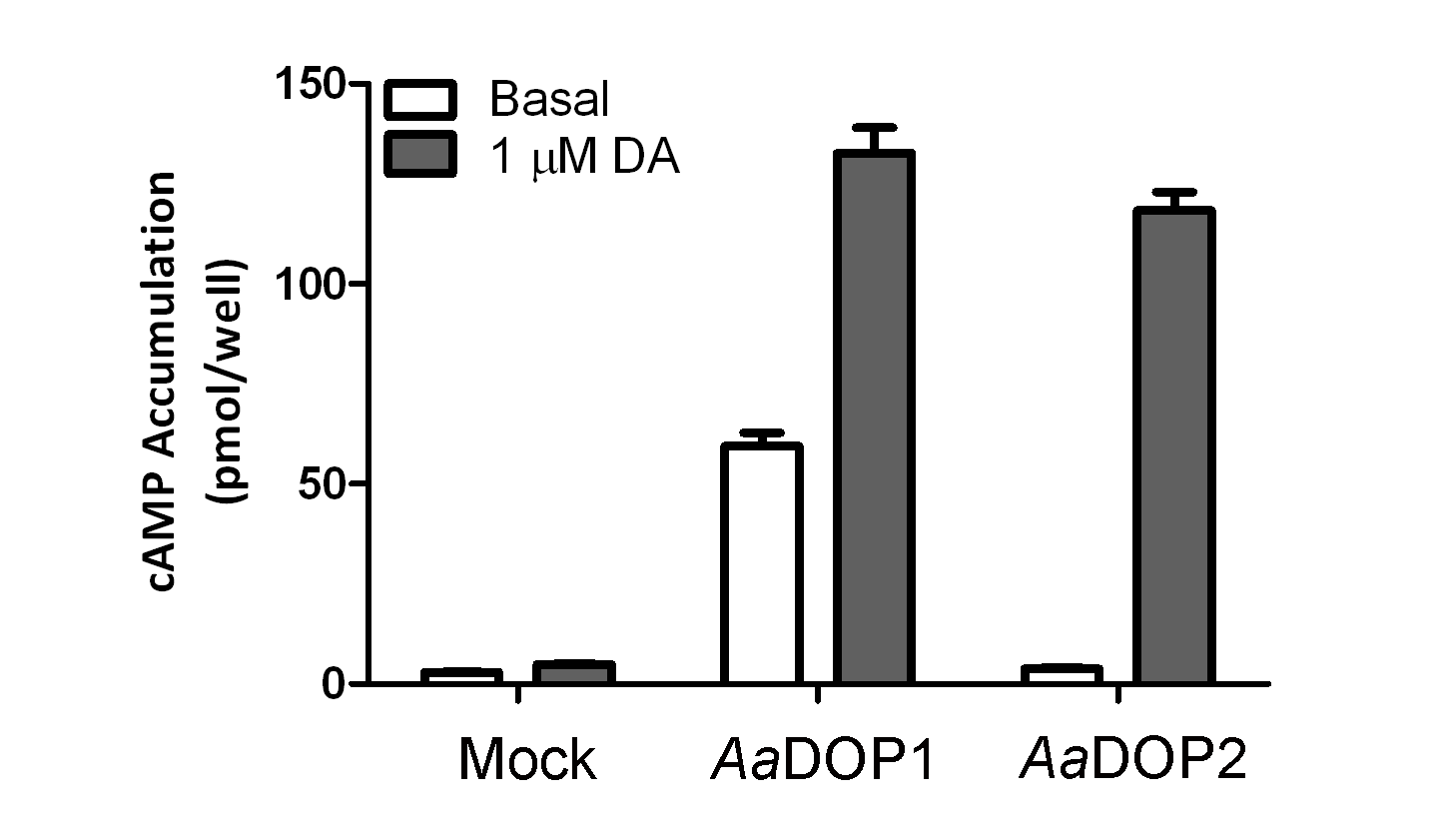

Supplement: Figure S5 — Response of Aa DOP1 and Aa DOP2 following dopamine treatment in transiently-transfected HEK cells. Significant responses to dopamine were observed for both AaDOP1 and AaDOP2, relative to basal conditions (p<0.05). (TIF) [file pntd.0001478.s005.tif]
