# Supplementary material for: A “Genome-to-Lead” Approach for Insecticide Discovery: Pharmacological Characterization and Screening of Aedes aegypti D1-like Dopamine Receptors
Source: PLoS Negl Trop Dis. 2012 Jan 24;6(1):e1478. doi: 10.1371/journal.pntd.0001478 (PMC3265452; doi:10.1371/journal.pntd.0001478)
Supplement: Table S1 — Primer pairs and experimental conditions used in RT-PCR analysis of Aadop1 and Aadop2 transcripts. (DOC) [file pntd.0001478.s006.doc]

**Table S1. Primer pairs and experimental conditions used in RT-PCR analysis of *Aadop1* and *Aadop2* transcripts.**

| **Gene** | **Primer name / Sequence** | **Exons joined by RT-PCR Product** | **Expected Product Size (bp)** | **Annealing Temperature (˚C)** | **Presence (+) / Absence (-) of RT-PCR Product^a^** |
| --- | --- | --- | --- | --- | --- |
| *Aadop1* | *Aadop1*_Full_F | 1-4 | 1233 | 45/50 | **- / -** |
|  | 5’-aatacgattgggattttttg-3’ |
|  | *Aadop1*_Full_R |
|  | 5’-gatggcggatacctgttcgag-3’ |
|  | *Aadop1*_1F | 1-2 | 224 | 50 | **+** |
|  | 5’-tttctctccgtagccggtaa-3’ |
|  | *Aadop1*_1R |
|  | 5’-gcggttgaacacatgacatc-3’ |
|  | *Aadop1*_1F | 1-4 | 1058 | 50 | **+** |
|  | 5’-tttctctccgtagccggtaa-3’ |
|  | *Aadop1*_2R |
|  | 5’-ggcggatacctgttcgagat-3’ |
| *Aadop2* | *Aadop2*_Full_F | 1-4 | 1425 | 50 | **+** |
|  | 5’-aataatgcaactgacttctac-3’ |
|  | *Aadop2*_Full_R |
|  | 5’-gatatacgtctgctcgcaagag-3’ |

^a^Data reflect RT-PCR results using total RNA samples extracted from adult female *A. aegypti*.
