# Supplementary material for: A “Genome-to-Lead” Approach for Insecticide Discovery: Pharmacological Characterization and Screening of Aedes aegypti D1-like Dopamine Receptors
Source: PLoS Negl Trop Dis. 2012 Jan 24;6(1):e1478. doi: 10.1371/journal.pntd.0001478 (PMC3265452; doi:10.1371/journal.pntd.0001478)
Supplement: Table S2 — Summary of selected amino acid features of Aedes aegypti Aa DOP1 and Aa DOP2. (DOC) [file pntd.0001478.s007.doc]

**Table S2. Summary of selected amino acid features of *Aedes aegypti Aa*DOP1 and *Aa*DOP2*.***

| **Protein features** | **Amino acids in *Aa*DOP1** | **Amino acids in *Aa*DOP2** |
| --- | --- | --- |
| Total size^a^ | 412 | 476 |
| Size of N-terminus^a^ | 41 | 57 |
| Size of intracellular loops I, II, III^a^ | 10, 20, 62 | 10, 20, 115 |
| Size of extracellular loops I, II, III^a^ | 14, 28, 7 | 15, 18, 9 |
| Size of carboxyl tail^a^ | 61 | 63 |
| 1-4 *N* linked glycosylation sites (N-terminus) | N.P.^g^ | N3, N19, N24, N46 |
| Conserved cysteines in extracellular loops 1-II^b^ | C115, C204 | C132, C211 |
| C-terminus palmitoylation sites | C370, C371 | C426, C428 |
| Protein kinase A/C phosphorylation (Intracellular loops and C-terminus) | S72, T155, S245, S262, S269, T364, T365, S368, S383, S399 | S166, T172, T250, S252, T278, T305, S339, S341, T440, S446, S451, S456, T457, S459, S470 |
| Conserved aspartate in TM II + III^c^ | D88, D122 | D104, D139 |
| Conserved “DRY” motif^d^ | D139, R140, Y141 | D156, R157, Y158 |
| Conserved serines in TM V^e^ | S216, S217, S220 | S223, S224, S227 |
| Conserved aromatic residues in TM V^f^ | F221 | F228 |
| Conserved aromatic residues in TM VI^f^ | W308, F311, F312 | W368, F371, F372 |

^a^Values refer to the number of amino acids composing these features

^b^Presumed to form a disulfide bond for protein stabilization

^c^Predicted as important for binding the amine moieties of catecholamines

^d^Implicated in G-protein coupling

^e^Predicted to form hydrogen bonds with catechol hydroxyl groups

^f^Aromatic residues implicated in ligand interaction

^g^N.P. = not predicted
