# Supplementary material for: A “Genome-to-Lead” Approach for Insecticide Discovery: Pharmacological Characterization and Screening of Aedes aegypti D1-like Dopamine Receptors
Source: PLoS Negl Trop Dis. 2012 Jan 24;6(1):e1478. doi: 10.1371/journal.pntd.0001478 (PMC3265452; doi:10.1371/journal.pntd.0001478)
Supplement: Table S3 — Comparison of transmembrane domains of A. aegypti Aa DOP1 and Aa DOP2 and related D1-like receptors. (DOC) [file pntd.0001478.s008.doc]

**Table S3. Comparison of transmembrane domains of *A. aegypti Aa*DOP1 and *Aa*DOP2 and related D_1_-like receptors.**

| **Receptor** | **Percent amino acid identity in TM domains**^a^ | | | | | | | |
| --- | --- | --- | --- | --- | --- | --- | --- | --- |
|  | ***D. melanogaster*** | | ***A. mellifera*** | | ***I. scapularis*** | | ***H. sapiens*** | |
|  | **D-Dop1** | **DopR99B** | ***Am*DOP1** | ***Am*DOP2** | ***Is*dop1** | ***Is*dop2** | **D1** | **D5** |
| *Aa*DOP1 | 88 | 47 | 81 | 44 | 70 | 47 | 54 | 52 |
| *Aa*DOP2 | 48 | 97 | 51 | 89 | 48 | 73 | 51 | 47 |

^a^Calculations of percent identity were based on the aligned TM sequences shown in Figure S2.
