# Supplementary material for: A “Genome-to-Lead” Approach for Insecticide Discovery: Pharmacological Characterization and Screening of Aedes aegypti D1-like Dopamine Receptors
Source: PLoS Negl Trop Dis. 2012 Jan 24;6(1):e1478. doi: 10.1371/journal.pntd.0001478 (PMC3265452; doi:10.1371/journal.pntd.0001478)
Supplement: Table S4 — Results of the Aedes aegypti Aa DOP2 antagonist screen of the LOPAC1280 library. (PDF) [file pntd.0001478.s009.pdf]

Table S4. Results of the *Aedes aegypti Aa* DOP2 antagonist screen of the $LOPAC_{1280}$ library

Compound Number	Compound Common Name	Compound effect[†]	Compound Chemical Name	Chemical Class	Molecular Weight
1	DL-alpha-Methyl-p-tyrosine	95	4-Hydroxy-alpha-methylphenylalanine	Neurotransmission	195.22
2	N-Phenylanthranilic acid	100	Diphenylamine-2-carboxylic acid; DPC	Cl- Channel	213.24
3	S(-)-p-Bromotetramisole oxalate	106	R 30402 oxalate	Phosphorylation	373.23
4	5-Aminovaleric acid hydrochloride	115	5-Aminopentanoic acid hydrochloride	GABA	153.61
5	(±)-Nipecotic acid	99	(±)-3-Piperidine carboxylic acid	GABA	129.16
6	Azelaic acid	107	Dicarboxylic acid C9; Nonanedioic acid; AZA	DNA Metabolism	188.23
7	Tryptamine hydrochloride	121	3-(2-Aminoethyl)indole hydrochloride	Serotonin	196.68
8	5-Fluoroindole-2-carboxylic acid	92		Glutamate	179.15
9	6-Methoxy-1,2,3,4-tetrahydro-9H-pyrido[3,4b] indole	116		Neurotransmission	202.26
10	S-(4-Nitrobenzyl)-6-thioguanosine	105	2-Amino-6-[(4-Nitrobenzyl)thio]-9-beta-D-ribofuranosylpurine	Adenosine	434.43
11	TMB-8 hydrochloride	98	8-(Diethylamino)octyl 3,4,5-trimethoxybenzoate hydrochloride	Intracellular Calcium	432.00
12	4-Aminopyridine	110		K+ Channel	94.12
13	Atropine sulfate	78		Cholinergic	676.83
14	Atropine methyl nitrate	112	AMN; Methylatropine nitrate	Cholinergic	366.42
15	Arcaine sulfate	122	N,N'-1,4-Butanediylbis(guanidine) sulfate	Glutamate	270.31
16	1-Aminocyclopropanecarboxylic acid hydrochloride	104	ACPC	Glutamate	137.57
17	Acetamide	108	Amide C2	Biochemistry	59.07
18	N-(4-Aminobutyl)-5-chloro-2-naphthalenesulfonamide hydrochloride	97	W-13	Intracellular Calcium	349.28
19	L-azetidine-2-carboxylic acid	115	(S)-Azetidine-2-carboxylic acid	Biochemistry	101.11
20	p-Aminoclonidine hydrochloride	79	Apraclonidine hydrochloride	Adrenoceptor	281.57
21	3-aminobenzamide	96	3-ABA; 3-AB	Apoptosis	136.15
22	(±)-Norepinephrine (+)bitartrate	102	(±)-Arterenol (+)bitartrate; (±)-Noradrenalin (+)bitartrate	Adrenoceptor	319.27
23	4-Amino-1,8-naphthalimide	104		Apoptosis	212.21
24	Reserpine	113	Methyl reserpate; 3,4,5-Trimethoxybenzoic acid ester	Serotonin	608.69
25	Amantadine hydrochloride	84	Tricyclo[3.3.1.1 3,7]decan-1-amine hydrochloride	Dopamine	187.71
26	Aminophylline ethylenediamine	129	Theophylline ethylenediamine	Adenosine	420.43
27	S-(p-Azidophenacyl)glutathione	105		Multi-Drug Resistance	466.48
28	Aminopterin	112	4-Aminofolic acid	Antibiotic	440.42
29	N-Acetyl-5-hydroxytryptamine	110	N-Acetylserotonin; Normelatonin	Melatonin	218.26
30	Aurintricarboxylic acid	132	ATA	Apoptosis	422.35
31	(±)-2-Amino-4-phosphonobutyric acid	124	(±)-AP-4	Glutamate	183.10
32	N-arachidonylglycine	116	NAGly	Cannabinoid	361.53
33	GABA	102	gamma-Aminobutyric acid	GABA	103.12
34	3'-Azido-3'-deoxythymidine	114	Azidothymidine; AZT	Immune System	267.25
35	Acetyl-beta-methylcholine chloride	127	Methacholine chloride	Cholinergic	195.69
36	5-azacytidine	103	4-Amino-1-(beta-D-ribofuranosyl)-1,3,5-triazin-2(1H)-one; Ladakamycin	DNA Metabolism	244.21
37	5-(N-Ethyl-N-isopropyl)amiloride	75	EIPA	Ion Pump	299.77
38	3-Aminopropionitrile fumarate	100		Multi-Drug Resistance	256.26
39	Apigenin	21	4',5,7-Trihydroxyflavone	Cell Cycle	270.24
40	W-7 hydrochloride	87	N-(6-Aminohexyl)-5-chloro-1-naphthalenesulfonamide hydrochloride	Intracellular Calcium	377.34
41	Gabaculine hydrochloride	106		GABA	175.62
42	AC 915 oxalate	107	N-(2-(3,4-dichlorophenyl)acetoxy)ethylpyrrolidine oxalate	Opioid	392.24
43	AA-861	69	2-(12-Hydroxydodeca-5,10-diynyl)-3,5,6-trimethyl-p-benzoquinone	Leukotriene	326.44
44	9-Amino-1,2,3,4-tetrahydroacridine hydrochloride	206	Tacrine hydrochloride; THA hydrochloride	Cholinergic	234.73
45	AL-8810	102	(5Z, 13E)-(9S,11S,15R)-9,15,dihydroxy-11-fluoro-15-(2-indanyl)-16,17,18,19,20,pentanor-5,13-prostadienoic acid	Prostaglandin	402.51
46	1-Aminobenzotriazole	141	ABT; 1-Benzotriazolamine	Multi-Drug Resistance	134.14
47	3-Amino-1-propanesulfonic acid sodium	94		GABA	161.16
48	Apomorphine hydrochloride hemihydrate	54	10,11-Dihydroxyaporphine hydrochloride hemihydrate	Dopamine	303.79
49	O-(Carboxymethyl)hydroxylamine hemihydrochloride	97	Aminooxyacetic acid; (Carboxymethoxy)amine hemihydrochloride	Biochemistry	218.60
50	5-(N,N-Dimethyl)amiloride hydrochloride	108	☐3-Amino-N-(aminoiminoethyl)-5-(dimethylamino)-6-chloropyrazinecarboxamide hydrochloride; DMA	Ion Pump	294.15
51	Azathioprine	103		P2 Receptor	277.27
52	Acyclovir	90	Acycloguanosine	Immune System	225.21
53	Amiprilose hydrochloride	99	1,2-O-Isopropylidene-3-O-[3'-(N,N-dimethylamino)propyl]-alpha-D-glucofuranose hydrochloride	Immune System	341.84
54	Alsterpaullone	92	9-Nitro-7,12-dihydroindolo-[3,2-d][1]benzazepin-6(5)-one	Phosphorylation	293.28
55	(±)-2-Amino-3-phosphonopropionic acid	102	(±)-AP-3	Glutamate	169.07
56	L-Arginine	94		Nitric Oxide	174.20
57	(±)-2-Amino-7-phosphonoheptanoic acid	93	(±)-AP-7	Glutamate	225.18
58	(±)-2-Amino-5-phosphonopentanoic acid	89	(±)-AP-5; (±)-AP-V	Glutamate	197.13
59	L-732,138	100	N-Acetyl-L-tryptophan 3,5-bis(trifluoromethyl)benzyl ester	Tachykinin	472.39
60	Acetylsalicylic acid	87	O-Acetylsalicylic acid; Aspirin	Prostaglandin	180.16
61	5-(N-Methyl-N-isobutyl)amiloride	72	MIA	Ion Pump	299.77
62	Acetylthiocholine chloride	122		Cholinergic	197.73
63	4-Androsten-4-ol-3,17-dione	107	4-OH-A; 4-Hydroxy-4-androstene-3,17-dione	Hormone	302.42
64	2-(2-Aminoethyl)isothiourea dihydrobromide	104	AET; S-(2-Aminoethyl)isothiouronium dihydrobromide	Nitric Oxide	281.01

65	N-Acetylprocainamide hydrochloride	126	Acedainide; N-Acetylnovocainamide hydrochloride; NAPA	Na+ Channel	313.83
66	Sodium Taurocholate	115	3alpha,7alpha,12alpha-Trihydroxy-5beta-cholan-24-oic-acid N-(2-sulfoethyl)amide	Multi-Drug Resistance	537.70
67	Amifostine	111	2-(3-Aminopropyl)aminoethyl phosphorothioate; WR2721	Cell Stress	214.22
68	Acetazolamide	106	N-[5-(Aminosulfonyl)-1,3,4-thiadiazol-2-yl]acetamide	Biochemistry	222.25
69	Arecoline hydrobromide	104	1-Methyl-1,2,5,6-tetrahydro-3-pyridinecarboxylic acid methyl ester hydrobromide	Cholinergic	236.11
70	A-315456	111	N-[3-(cyclohexylidene(1H-imidazol-4-ylmethyl)phenyl]ethanesulfonamide	Adrenoceptor	345.47
71	GR 4661	114	3-[3-(2-Dimethylaminoethyl)-1H-indol-5-yl]-N-(4-methoxybenzyl)acrylamide	Serotonin	377.49
72	2-Hydroxysaclofen	110	(±)-3-Amino-2-(4-chlorophenyl)-2-hydroxy-propylsulfonic acid	GABA	265.72
73	Actinonin	96	3-[[1-[(2-(Hydroxymethyl)-1-pyrrolidinyl)carbonyl]-2-methylpropyl]carbamoyl]octanohydroxamic acid	Biochemistry	385.51
74	Methotrexate	89	Methylaminopterin; MTX	DNA Metabolism	454.45
75	Atropine methyl bromide	105		Cholinergic	384.32
76	Amperozide hydrochloride	17	4-[4,4-bis(4-Fluorophenyl)butyl]-N-ethyl-1-piperazinecarboxamide hydrochloride	Serotonin	437.96
77	Aminoguanidine hemisulfate	107	Hydrazinecarboximidamide hemisulfate	Nitric Oxide	246.25
78	Agmatine sulfate	113	(4-Aminobutyl)guanadine sulfate	Imidazoline	228.27
79	4-Aminobenzamidine dihydrochloride	106		Biochemistry	208.09
80	3-Aminopropylphosphonic acid	101		GABA	139.09
81	N-Acetyl-L-Cysteine	101		Glutamate	163.20
82	L-2-aminoadipic acid	96	Aad; (S)-2-Aminohexanedioic acid; L-Homoglutamic acid	Glutamate	161.16
83	N-Acetyltryptamine	42	3-(2-N-Acetylaminoethyl)indole	Melatonin	202.26
84	Amiloride hydrochloride	110		Na+ Channel	266.09
85	(±)-Atenolol	121		Adrenoceptor	266.34
86	5alpha-Androstane-3alpha,17beta-diol	98	Dihydroandrosterone; 3alpha,17beta-Dihydroxy-5alpha-androstane	Hormone	292.47
87	L-allylglycine	107	L-2-Amino-4-pentenoic acid	Biochemistry	115.13
88	H-9 dihydrochloride	64	N-(2-Aminoethyl)-5-isoquinolinesulfonamide dihydrochloride	Phosphorylation	324.23
89	6-Aminohexanoic acid	120	6-Aminocaproic acid; EACA	Immune System	131.18
90	ATPO	109	(R,S)-2-Amino-3-[5-tert-butyl-3-(phosphonomethoxy)-4-isoxazolyl]propionic acid	Glutamate	322.26
91	Allopurinol	101	1H-Pyrazolo[3,4-d]pyrimidin-4-ol	Cell Stress	136.11
92	Amitriptyline hydrochloride	10		Adrenoceptor	313.87
93	Amiodarone hydrochloride	104		Adrenoceptor	681.78
94	4-(2-Aminoethyl)benzenesulfonyl fluoride hydrochloride	105	AEBSF	Biochemistry	239.70
95	Ancitabine hydrochloride	119	Cyclocytidine hydrochloride; Cyclo-C	DNA Metabolism	261.67
96	Alprenolol hydrochloride	104		Adrenoceptor	285.82
97	Altretamine	105	N,N,N',N',N'',N''-Hexamethyl-1,3,5-triazine-2,4,6-triamine	DNA Metabolism	210.28
98	N-Acetyldopamine monohydrate	95		Dopamine	195.22
99	Aminoguanidine hydrochloride	107	Guanylhydrazine hydrochloride	Nitric Oxide	110.55
100	BW 284c51	93	1,5-Bis(4-allyldimethylammoniumphenyl)pentan-3-one dibromide	Cholinergic	566.42
101	Adenosine	96		Adenosine	267.25
102	L-Aspartic acid	106		Glutamate	133.10
103	Astaxanthin	106	3,3'-Dihydroxy-beta,beta-carotene-4,4'-dione	Cell Stress	596.86
104	N-(4-Amino-2-chlorophenyl)phthalimide	99		Anticonvulsant	272.69
105	Adenosine 3',5'-cyclic monophosphate	100	cAMP; 3',5'-Cyclic AMP	Phosphorylation	329.21
106	L(-)-Norepinephrine bitartrate	115	(-)-Arterenol bitartrate; Noradrenaline bitartrate	Adrenoceptor	319.27
107	5-(N,N-hexamethylene)amiloride	82		Ion Pump	311.78
108	4-Androstene-3,17-dione	115	Androstenedione; 3,17-Dioxo-4-andostene	Hormone	286.42
109	(±)-p-Aminoglutethimide	106		Biochemistry	232.28
110	(±)-HA-966	107	(±)-3-Amino-1-hydroxy-2-pyrrolidone	Glutamate	116.12
111	Androsterone	109	5a-Androstan-3a-ol-17-one; cis-Androsterone	Hormone	290.45
112	Amsacrine hydrochloride	97	m-AMSA hydrochloride	DNA Repair	429.93
113	(±)-AMT hydrochloride	103	(±)-2-Amino-5,6-dihydro-6-methyl-4H-1,3-thiazine hydrochloride	Nitric Oxide	166.67
114	(-)Amethopterin	116		DNA Metabolism	454.45
115	Antozoline hydrochloride	115	2-(N-Benzylanilinomethyl)-2-imidazoline hydrochloride	Imidazoline	299.81
116	Aniracetam	101		Glutamate	219.24
117	1,3-Diethyl-8-phenylxanthine	105	DPX	Adenosine	284.32
118	8-(p-Sulfophenyl)theophylline	98		Adenosine	336.33
119	1,3-Dipropyl-8-p-sulfophenylxanthine	91		Adenosine	392.44
120	2-Methylthioadenosine triphosphate tetrasodium	99	2-Methylthio ATP tetrasodium	P2 Receptor	641.20
121	5'-N-Methyl carboxamidoadenosine	114	MECA	Adenosine	294.27
122	Adenosine amine congener	105	ADAC	Adenosine	576.62
123	(+)-N-Allylnormetazocine hydrochloride	90	(+)-NANM hydrochloride; SKF-10047	Opioid	293.84
124	Amoxapine	10		Adrenoceptor	313.79
125	Aminobenztropine	30	ABT	Cholinergic	322.45
126	Arecaidine propargyl ester hydrobromide	85	APE	Cholinergic	260.13
127	R(+)-Atenolol	91		Adrenoceptor	266.34
128	S(-)-Atenolol	90		Adrenoceptor	266.34
129	1-Allyl-3,7-dimethyl-8-p-sulfophenylxanthine	100		Adenosine	376.39
130	trans-(±)-ACPD	116	trans-(±)-1-Amino-1,3-cyclopentanedicarboxylic acid	Glutamate	173.17
131	(±)-N-Allylnormetazocine hydrochloride	105	(±)-NAMM hydrochloride; (±)-SKF-10047	Opioid	293.84
132	1-Amino-1-cyclohexanecarboxylic acid hydrochloride	110		Neurotransmission	179.65

133	Alaproclate hydrochloride	110	D,L-Alanine, 2-(4-chlorophenyl)-1,1-dimethylethyl ester hydrochloride	Serotonin	292.21
134	Rp-cAMPS triethylamine	67	Rp-Adenosine 3',5'-cyclic monophosphothioate triethylamine	Phosphorylation	446.47
135	SB 200646 hydrochloride	106	N-(1-Methyl-1H-indol-5-yl)-N'-3-pyridinyl-urea hydrochloride	Serotonin	304.78
136	D(-)-2-Amino-7-phosphonoheptanoic acid	102	D-AP-7	Glutamate	225.18
137	Acetohexamide	115		Hormone	324.40
138	SKF 97541 hydrochloride	101	3-Aminopropyl-(methyl)phosphinic acid hydrochloride	GABA	173.58
139	cis-4-Aminocrotonic acid	95	CACA	GABA	101.11
140	N6-2-(4-Aminophenyl)ethyladenosine	97	APNEA	Adenosine	386.41
141	Agroclavine	98	8,9-Didehydro-6,8-dimethyl-ergoline	Dopamine	238.34
142	gamma-Acetylinic GABA	104	4-Amino-5-hexynoic acid	GABA	127.14
143	AB-MECA	100	N6-(4-Aminobenzyl)-9-[5-(methylcarbonyl)-beta-D-ribofuranosyl]adenine	Adenosine	399.41
144	Alloxazine	104	Isoalloxazine	Adenosine	214.18
145	cis-Azetidine-2,4-dicarboxylic acid	112		Glutamate	145.12
146	trans-Azetidine-2,4-dicarboxylic acid	110	tADA	Glutamate	145.12
147	AGN 192403 hydrochloride	109	(2-endo,3exo)-3-(Methylethyl)-bicyclo[2.2.1]heptan-2-amine hydrochloride	Imidazoline	189.73
148	AIDA	95	1-Aminoindan-1,5-dicarboxylic acid; UPF 523	Glutamate	221.21
149	A-77636 hydrochloride	113		Dopamine	365.90
150	ATPA	101	(RS)-2-Amino-3-(3-hydroxy-5-tert-butylisoxazol-4-yl)propanoic acid	Glutamate	228.25
151	ARL 67156 trisodium salt	115	FPL 67156	P2 Receptor	785.06
152	Beclomethasone	97	9alpha-Chloro-16beta-methyl-1,4-pregnadiene-11beta,17alpha,21-triol-3,20-dione	Hormone	408.93
153	2,3-Butanedione monoxime	131	Diacetyl monoxime	K+ Channel	101.11
154	SB 222200	92		Tachykinin	380.49
155	1-benzoyl-5-methoxy-2-methylindole-3-acetic acid	105		Multi-Drug Resistance	323.35
156	p-Benzoquinone	96	Quinone; p-BQ	DNA Repair	108.10
157	8-Bromo-cGMP sodium	117	8-Bromoguanosine-3',5'-cyclophosphate sodium	Cyclic Nucleotides	446.09
158	H-89	46	N-[2-(p-Bromocinnamylamino)ethyl]-5-isoquinolinesulfonamide dihydrobromide	Phosphorylation	519.29
159	Bromoenol lactone	103	BEL; E-6-(Bromoethylene)tetrahydro-3-(1-naphthyl)-2H-pyran-2-one	Lipid	317.18
160	Benzamide	100	Benzoylamide	Apoptosis	121.14
161	3-Bromo-7-nitroindazole	83		Nitric Oxide	242.03
162	(+)-Bromocriptine methanesulfonate	37		Dopamine	750.72
163	O6-benzylguanine	80		DNA Repair	241.25
164	N-Bromoacetamide	104	NBA	Na+ Channel	137.96
165	(±)-Brompheniramine maleate	63		Histamine	435.32
166	Benzamil hydrochloride	60	N-(Benzylamidino)-3,5-diamino-6-chloropyrazinecarboxamide hydrochloride	Ion Pump	356.22
167	L-Buthionine-sulfoximine	81		Multi-Drug Resistance	222.31
168	DL-Buthionine-[S,R]-sulfoximine	77		Multi-Drug Resistance	222.31
169	ND	90	ND	ND	ND
170	Betaine hydrochloride	86		Biochemistry	153.61
171	Betaine aldehyde chloride	99	(Formylmethyl)trimethylammonium chloride	Cholinergic	137.61
172	Benazoline oxalate	109	4,5-Dihydro-2-(2-naphthalenyl)-1H-imidazole oxalate	Imidazoline	320.37
173	BWB70C	118	N-[3-[3-4(-Fluorophenoxy)phenyl]-1-methyl-2-propenyl]-N-hydroxyurea	Leukotriene	316.34
174	5-Bromo-2'-deoxyuridine	84	Br-dU; 5-Bromo-1-(2-deoxy-beta-D-ribofuranosyl)uracil	DNA Metabolism	307.10
175	Bepridil hydrochloride	78		Ca2+ Channel	403.01
176	(+)-Brompheniramine maleate	97	Dexbrompheniramine maleate	Histamine	435.32
177	(±)-Baclofen	111	Lioresal	GABA	213.67
178	SB 202190	65	4-[4-(4-Fluorphenyl)-5-(4-pyridinyl)-1H-imidazol-2-yl]phenol	Phosphorylation	331.35
179	Bay 11-7085	76	(E)-3-(4-t-Butylphenylsulfonyl)-2-propenenitrile	Cell Cycle	249.33
180	Betaxolol hydrochloride	95		Adrenoceptor	343.90
181	Benzamidine hydrochloride	87	Amidinobenzene hydrochloride	Biochemistry	156.62
182	Betamethasone	89	9alpha-Fluoro-16beta-methylprednisolone	Hormone	392.47
183	Buspirone hydrochloride	83		Serotonin	421.97
184	Benserazide hydrochloride	94	DL-Serine, 2-[(2,3,4-trihydroxyphenyl)methyl]hydrazide hydrochloride	Biochemistry	293.71
185	Brefeldin A from Penicillium brefeldianum	124	BFA; Ascotoxin, Cyanein	Cytoskeleton and ECM	280.37
186	Budesonide	128	16,17-Butylidenebis(oxy)-11,21-dihydroxypregna-1,4-diene-3,20-dione	Hormone	430.55
187	8-Bromo-cAMP sodium	109	8-Bromoadenosine-3',5'-cyclophosphate sodium	Cyclic Nucleotides	430.09
188	Benztropine mesylate	11		Cholinergic	403.54
189	Ro 20-1724	122	4-[(3-Butoxy-4-methoxyphenyl)methyl]-2-imidazolidione	Cyclic Nucleotides	278.35
190	Bestatin hydrochloride	145	N-[(2S,3R)-3-Amino-2-hydroxy-4-phenylbutyryl]-L-leucine hydrochloride	Biochemistry	344.84
191	Bretylium tosylate	97	2-Bromo-N-ethyl-N,N-dimethylbenzenemethanaminium 4-methyl-benzenesulfontate	Adrenoceptor	414.36
192	(+)-Bicuculline	94		GABA	367.36
193	BP 897	54	N-[4-(4-(2-methoxyphenyl)piperazinyl)butyl]-2-naphthamide	Dopamine	417.56
194	(E)-5-(2-Bromovinyl)-2'-deoxyuridine	121	BVdU	Immune System	333.14
195	BRL 15572	84	4-(3-Chlorophenyl)-alpha-(diphenylmethyl)-1-piperazineethanol hydrochloride	Serotonin	443.42
196	Chloroethylclonidine dihydrochloride	87	CEC dihydrochloride	Adrenoceptor	408.59
197	6-Fluoronorepinephrine hydrochloride	90	6-FNE hydrochloride	Adrenoceptor	223.63
198	Bromoacetyl alprenolol menthane	87		Adrenoceptor	481.48
199	Benoxathian hydrochloride	68	2-[[[2-(2,6-Dimethoxyphenoxy)ethyl]-amino]-methyl]-1,4-benzoxanthian hydrochloride	Adrenoceptor	397.92
200	Phenoxybenzamine hydrochloride	86		Adrenoceptor	340.30
201	Bupropion hydrochloride	77	(±)-1-(3-Chlorophenyl)-2-[(1,1-dimethylethyl)amino]-1-propanone hydrochloride	Dopamine	276.21

202	(-)-Bicuculline methbromide, 1(S), 9(R)	94		GABA	462.30
203	(±)-Bay K 8644	73	1,4-Dihydro-2,6-dimethyl-5-nitro-4-[2-(trifluoromethyl)-phenyl]- 3-pyridine carboxylic acid methyl ester	Ca2+ Channel	356.30
204	Bromoacetylcholine bromide	91	2-(2-Bromoacetyloxy)-N,N,N-trimethylethanaminium bromide	Cholinergic	305.01
205	BMY 7378 dihydrochloride	102	8-[2-[4-(2-Methoxyphenyl)-1-piperazinyl]ethyl- azaspiro[4.5]decane-7,9-dione dihydrochloride	Serotonin	458.43
206	R(+)-6-Bromo-APB hydrobromide	182	R(+)-6-Bromo-7,8-dihydroxy-3-allyl-1-phenyl-2,3,4,5-tetrahydro- 1H-3-benzazepine hydrobromide	Dopamine	455.19
207	BTCP hydrochloride	100		Dopamine	335.94
208	N6-Benzyl-5'-N-ethylcarboxamidoadenosine	108	N6-Benzyl-NECA	Adenosine	398.42
209	BU224 hydrochloride	92	2-(4,5-Dihydroimidazol-2-yl)-quinoline hydrochloride	Imidazoline	233.70
210	B-HT 933 dihydrochloride	79	Azepexole dihydrochloride	Adrenoceptor	254.16
211	(±)-Butaclamol hydrochloride	20	AY 23028	Dopamine	397.99
212	BRL 37344 sodium	87	(±)-(R*,R*)-[4-[2-[[2-(3-Chlorophenyl)-2-hydroxyethyl]□amino]propyl]phenoxy]-acetic acid sodium	Adrenoceptor	385.83
213	BRL 54443 maleate	80	3-(1-Methylpiperidin-4-yl)-1H-indol-5-ol maleate	Serotonin	346.39
214	BW 723C86	92		Serotonin	322.86
215	Chlorambucil	106	4-[Bis(2-chloroethyl)amino]benzenebutyric acid	DNA	304.22
216	Citicoline sodium	91	CDP-coline; Citidine (5')-diphosphocholine sodium	Lipid	497.29
217	Ciprofibrate	97	2-[p-(2,2-Dichlorocyclopropyl)phenoxy]-2-methylpropanoic acid	Transcription	289.16
218	6-Chloromelatonin	90	N-Acetyl-6-chloro-5-methoxytryptamine	Melatonin	266.73
219	Carmustine	82	BCNU; 1,3-Bis(2-chloroethyl)-1-nitrosourea	DNA	214.05
220	PK 11195	84	1-(2-Chlorophenyl)-N-methyl-N-(1-methylpropyl)	GABA	352.87
221	Caffeic Acid	114	3,4-Dihydroxycinnamic acid	Cell Stress	180.16
222	Cilostazol	108	OPC 13013; OPC 21; Pletaal	Cyclic Nucleotides	369.47
223	Caffeine	92	1,3,7-Trimethylxanthine	Adenosine	194.19
224	Cyclophosphamide monohydrate	83	2-[Bis(2-chloroethyl)amino]tetrahydro-2H-1,3,2-oxazaphosphorine 2-oxide	DNA	261.09
225	CGP-7930	89	3-(3',5'-Di-tert-butyl-4'-hydroxy)phenyl-2,2-dimethylpropanol	GABA	292.47
226	CGP-13501	98	3-(3',5'-Di-tert-butyl-4'-hydroxy)phenyl-2,2-dimethylpropanal	GABA	290.45
227	CP55940	85	5-(1,1-dimethylheptyl)-2-[5-hydroxy-2-(3-hydroxypropyl)cyclohexyl]phenol	Cannabinoid	376.58
228	L-Cycloserine	88	(S)-4-Amino-3-isoxazolidone	Sphingolipid	102.09
229	ML-9	102		Phosphorylation	361.29
230	(+)-Catechin Hydrate	111	(+)-Cyanidol-3	Cell Stress	290.28
231	Chlorpropamide	98		Hormone	276.74
232	1-(4-Chlorobenzyl)-5-methoxy-2-methylindole-3-acetic acid	77		Multi-Drug Resistance	343.81
233	Chlorprothixene hydrochloride	6	2-Chloro-9-(3-dimethylaminopropylidene)thioxanthene hydrochloride	Dopamine	352.33
234	Choline bromide	89	Choline-methyl-13C bromide	Cholinergic	184.08
235	Ceramide	87		Phosphorylation	707.25
236	CB 1954	81	5-(1-Aziridinyl)-2,4-dinitrobenzamide	DNA	252.19
237	Carcinine dihydrochloride	95	beta-Alanylhistamine dihydrochloride	Cell Stress	255.15
238	Corticosterone	87	Kendall's Compound B; 4-Pregnene-11beta,21-diol-3,20-dione; Reichstein's Substance H	Hormone	346.47
239	Carboplatin	83	cis-Diammine(1,1-cyclobutanedicarboxylato) platinum	DNA	371.26
240	Cortisone	86	Kendall's Compound E; 4-Pregnene-17alpha,21-diol-3,11,20-trione; Reichstein's Substance Fa	Hormone	360.45
241	Chelerythrine chloride	112	1,2-Dimethoxy-N-methyl(1,3)benzodioxolo(5,6-c)phenanthridinium chloride	Phosphorylation	383.83
242	1-(2-Chlorophenyl)-1-(4-chlorophenyl)-2,2-dichloroethane	108	Mitotane	Hormone	320.05
243	(±)-Chlorpheniramine maleate	77		Histamine	390.87
244	Cortisone 21-acetate	110	21-Acetoxy-4-pregnen-17alpha-ol-3,11,20-trione	Hormone	402.49
245	Cephalosporin C zinc salt	109		Antibiotic	478.78
246	CGP-74514A hydrochloride	99	N2-(cis-2-Aminocyclohexyl)-N6-(3-chlorophenyl)-9-ethyl-9H-purine-2,6-diamine hydrochloride; Compound 13	Phosphorylation	422.36
247	Cyproterone acetate	115	6-Chloro-1beta,2beta-dihydro-17-hydroxy-3'H-cyclopropa(1,2)-pregna-1,4,6-triene-3,20-dione acetate	Hormone	416.95
248	DL-p-Chlorophenylalanine methyl ester hydrochloride	71		Neurotransmission	250.13
249	Cyclosporin A	119	Antibiotic S 7481F1	Phosphorylation	1202.64
250	D-Cycloserine	91	R(+)-4-Amino-3-isoxazolidinone	Glutamate	102.09
251	8-(4-Chlorophenylthio)-cAMP sodium	97		Cyclic Nucleotides	493.80
252	Calmidazolium chloride	44	R 24571 chloride	Intracellular Calcium	687.71
253	GR 113808	97	1-Methyl-1H-indole-3-carboxylic acid, [1-[2-[(methylsulfonyl)amino]ethyl]-4-piperidinyl]methyl ester	Serotonin	393.51
254	Carbamazepine	75	5H-Dibenz[b,f]azepine-5-carboxamide	Anticonvulsant	236.28
255	Captopril	112	(S)-1-(3-Mercapto-2-methyl-1-oxo-propyl)-L-proline	Neurotransmission	217.29
256	CNS-1102	107	N-(3-Ethylphenyl)-N-methyl-N'-1-naphthalenylguanidine monohydrochloride; Cerestat; Aptiganel hydrochloride	Glutamate	339.87
257	Carbachol	99	Carbamylcholine chloride	Cholinergic	182.65
258	Chlorzoxazone	95	5-Chloro-2(3H)-benzoxazolone	Nitric Oxide	169.57
259	L-Cysteinesulfinic Acid	116		Glutamate	153.16
260	9-cyclopentyladenine	116	9-CP-Ade	Cyclic Nucleotides	299.35
261	Cephalothin sodium	92		Antibiotic	418.43
262	Cimetidine	88	SKF-92334; Tagamet	Histamine	252.34
263	Cyclobenzaprine hydrochloride	106	5-(3-Dimethylaminopropylidene)dibenzo[a,e]cycloheptatriene hydrochloride	Serotonin	311.86
264	Carbetapentane citrate	93	1-Phenyl-cyclopentanecarboxylic acid 2-[2-(Diethylamino)ethoxy]ethyl ester citrate	Opioid	525.60
265	Cephalexin hydrate	87		Antibiotic	347.40
266	Chlorothiazide	109		Biochemistry	295.72
267	(+)-Chlorpheniramine maleate	106	(gammaS)-gamma-(4-Chlorophenyl)-N,N-dimethyl-2-pyridinepropanamine maleate	Histamine	390.87
268	Cefazolin sodium	96	Sodium CEZ; SKF-41558	Antibiotic	476.49
269	Clemizole hydrochloride	96	1-p-Chlorobenzyl-2-(1-pyrrolidinyl)methylbenzimidazole hydrochloride	Histamine	362.31
270	2-Chloroadenosine	118	2-CADO	Adenosine	301.69

271	Bethanechol chloride	116	Carbamyl-beta-methylcholine chloride	Cholinergic	196.68
272	Cinnarizine	100	1-trans-Cinnamyl-4-diphenylmethylpiperazine	Ca2+ Channel	368.53
273	1-(3-Chlorophenyl)piperazine dihydrochloride	99	m-CPP dihydrochloride	Serotonin	269.60
274	SB 204741	95	N-(1-Methyl-1H-indo-5-yl)-N'-(3-methyl-5-isothiazolyl)urea	Serotonin	286.36
275	Ceftriaxone sodium	97	Ro-13-9904/001	Antibiotic	598.55
276	4-Chloromercuribenzoic acid	96	4-(Hydroxymercuri)benzoic acid	Biochemistry	357.16
277	(-)-Cotinine	96	S(-)-1-Methyl-5-(3-pyridyl)-2-pyrrolidone	Cholinergic	176.22
278	CL 316,243	101	Disodium 5-[(2R)-2-[[(2R)-2-(3-Chlorophenyl)-2-hydroxyethyl]amino]propyl]-1,3-benzodioxole-2,2-dicarboxylate	Adrenoceptor	465.80
279	7-Chloro-4-hydroxy-2-phenyl-1,8-naphthyridine	100		Adenosine	256.69
280	Clotrimazole	77	1-(o-Chlorotrityl)imidazole	K+ Channel	344.85
281	Cyproheptadine hydrochloride	86		Serotonin	323.87
282	5'-(N-Cyclopropyl)carboxamidoadenosine	82	CPCA	Adenosine	320.31
283	Cefmetazole sodium	74	CS-1170; SKF-83088	Antibiotic	493.52
284	Clozapine	19	8-Chloro-11-(4-methyl)-1-piperazinyl)-5H-dibenzo[b,e][1,4]diazepine	Dopamine	326.83
285	(±)-p-Chlorophenylalanine	62	p-CPA	Neurotransmission	199.64
286	Chloroquine diphosphate	83		DNA	515.87
287	Clofibrate	74		Lipid	242.70
288	Cytosine-1-beta-D-arabinofuranoside hydrochloride	92	Arabinocytidine hydrochloride; Arabinosylcytosine hydrochloride; Ara-C hydrochloride	DNA Metabolism	279.68
289	CB34	106	N,N-Dipropyl-2-(4-chlorophenyl)-6,8-dichloro-imidazo[1,2-a]pyridine-3-acetamide	Benzodiazepine	438.79
290	Cefaclor	87		Antibiotic	367.81
291	DL-Cycloserine	94	4-amino-3-isoxazolidinone	Sphingolipid	102.09
292	McN-A-343	121	(4-Hydroxy-2-butynyl)-1-trimethylammonium-m-chlorocarbanilate chloride	Cholinergic	317.22
293	N-(2-[4-(4-Chlorophenyl)piperazin-1-yl]ethyl)-3-methoxybenzamide	95		Dopamine	373.89
294	Cystamine dihydrochloride	84	Decarboxycystine dihydrochloride	Glutamate	225.20
295	Clomipramine hydrochloride	105	Anafranil hydrochloride; Chloripramine	Serotonin	351.32
296	Calcimycin	102	A23187; Calcium ionophore A23187	Intracellular Calcium	523.63
297	Cantharidin	96	Cantharidine	Phosphorylation	196.20
298	Citalopram hydrobromide	90	1-[3-(Dimethylamino)propyl]-1-(4-fluorophenyl)-1,3-dihydro-5-isobenzofurancarbonitrile hydrobromide	Serotonin	405.31
299	Clonidine hydrochloride	100		Adrenoceptor	266.56
300	Cefotaxime sodium	93	Cefotaxim sodium	Antibiotic	477.45
301	Cilostamide	88	OPC 3689	Cyclic Nucleotides	342.44
302	Chelidamic acid	82	4-Hydroxypyridine-2,6-dicarboxylic acid	Glutamate	183.12
303	N6-Cyclopentyladenosine	89	CPA	Adenosine	335.37
304	Cantharidic Acid	120		Phosphorylation	214.22
305	Chlorpromazine hydrochloride	95		Dopamine	355.33
306	Cefsulodin sodium salt hydrate	108	Sulcephalosporin	Antibiotic	554.54
307	Caffeic acid phenethyl ester	147	CAPE	Cell Cycle	284.31
308	Cephapirin sodium	116	BL-P-1322	Antibiotic	445.45
309	Cephradine	79	Cefradin; SQ-11436	Antibiotic	349.41
310	DSP-4 hydrochloride	111	N-(2-Chloroethyl)-N-ethyl-2-bromobenzylamine hydrochloride	Adrenoceptor	313.07
311	Cinoxacin	135	1-Ethyl-1,4-dihydro-4-oxo[1,3]dioxolo[4,5-g]cinnoline-3-carboxylic acid	Antibiotic	262.22
312	Carisoprodol	130		Neurotransmission	260.34
313	Centrophenoxine hydrochloride	99	Meclofenoxate hydrochloride	Nootropic	294.18
314	Clemastine fumarate	82		Histamine	459.97
315	beta-Chloro-L-alanine hydrochloride	99		Biochemistry	160.00
316	Pyrocatechol	101	1,2-Benzenediol; Catechol	Cell Cycle	110.11
317	Z-L-Phe chloromethyl ketone	124	N-Carbobenzyloxy-L-phenylalanyl chloromethyl ketone; ZPCK	Biochemistry	331.80
318	CPCCOEt	58	7-(Hydroxyimino)cyclopropa[b]chromen-1a-carboxylate ethyl ester	Glutamate	247.25
319	Colchicine	131		Cytoskeleton and ECM	399.45
320	L-Canavanine sulfate	92	L-alpha-Amino-gamma-(guanidinooxy)butyric acid sulfate	Nitric Oxide	274.25
321	Cyclothiazide	93	6-Chloro-3,4-dihydro-3-(2-norbornen-5-yl)-2H-1,2-4-benzothiadiazine-7-sulfonamide 1,1-dioxide	Glutamate	389.88
322	N6-Cyclohexyladenosine	100	CHA	Adenosine	349.39
323	(S)-(+)-Camptothecin	7		Apoptosis	348.36
324	10-(alpha-Diethylaminopropionyl)-phenothiazine hydrochloride	56	As-1397	Biochemistry	362.92
325	(+)-cis-Dioxolane iodide	96	L(+)-cis-2-Methyl-4-trimethylammoniummethyl-1,3-dioxolane iodide	Cholinergic	287.14
326	OXA-22 iodide	88	cis-2-Methyl-5-trimethylammoniummethyl-1,3-oxathiolane iodide	Cholinergic	303.21
327	8-Cyclopentyl-1,3-dipropylxanthine	111	DPCPX; PD 116,948	Adenosine	304.40
328	8-Cyclopentyl-1,3-dimethylxanthine	93	CPT; 8-Cyclopentyltheophylline	Adenosine	248.29
329	(±)-CPP	91	(±)-3-(2-Carboxypiperazin-4-yl)propyl-1-phosphonic acid	Glutamate	252.21
330	CGS-12066A maleate	104	7-Trifluoromethyl-4(4-methyl-1-piperazinyl)-pyrrolo[1,2-a]quinoxaline maleate	Serotonin	450.42
331	2-Cyclooctyl-2-hydroxyethylamine hydrochloride	95	CONH hydrochloride	Neurotransmission	207.75
332	5-Carboxamidotryptamine maleate	119	5-CT; AH-21467	Serotonin	319.32
333	7-Chlorokynurenic acid	79	7-Cl-KYNA	Glutamate	223.62
334	(±)-CGP-12177A hydrochloride	97	4-[4-[(1,1-Dimethylethyl)amino]-2-hydroxypropoxy]- 1,3-dihydro-2H-benzimidazol-2-one hydrochloride	Adrenoceptor	315.80
335	S-(-)-Carbidopa	112		Biochemistry	226.23
336	(±)-Chloro-APB hydrobromide	77	(±)-SKF-82958 hydrobromide	Dopamine	410.74
337	CGS-21680 hydrochloride	112	2-p-(2-Carboxyethyl)phenethylamino-5'-N-ethylcarboxamidoadenosine hydrochloride	Adenosine	535.99
338	2-Chloro-N6-cyclopentyladenosine	92	CCPA	Adenosine	369.81
339	1-(m-Chlorophenyl)-biguanide hydrochloride	83	m-CPBG hydrochloride	Serotonin	248.12

340	2-Chloroadenosine triphosphate tetrasodium	102	2-Chloro-ATP tetrasodium	P2 Receptor	629.56
341	(+)-Cyclazocine	86		Opioid	271.41
342	Capsazepine	69	N-[2-(4-Chlorophenyl)ethyl]-1,3,4,5-tetrahydro- 7,8-dihydroxy-2H-2-benzazepine-2-carbothioamide	Vanilloid	376.91
343	Chlormezanone	93	2-(4-Chlorophenyl)tetrahydro-3-methyl-4H-1,3-thiazin-4-one 1,1-dioxide	Neurotransmission	273.74
344	8-(3-Chlorostyryl)caffeine	91	CSC	Adenosine	330.78
345	CGS-15943	117	9-Chloro-2-(2-furyl)[1,2,4]triazolo[1,5-c]quinazolin-5-amine	Adenosine	285.69
346	2-Chloro-2-deoxy-D-glucose	114	2-Chloro-DG	Biochemistry	198.60
347	4'-Chloro-3-alpha-(diphenylmethoxy)tropane hydrochloride	15		Dopamine	378.35
348	Cirazoline hydrochloride	74	2-[(2-Cyclopropylphenoxy)methyl]-4,5-dihydro-1H-imidazole hydrochloride	Adrenoceptor	252.75
349	CGP 20712A methanesulfonate	92	(+/-)-2-Hydroxy-5-[2-[[2-hydroxy-3-[4-[1-methyl-4-(trifluoromethyl)-1H-imidazol-2-yl]phenoxy]propyl]amino]ethoxy]-benzamide methanesulfonate	Adrenoceptor	590.58
350	(2S,1'S,2'S)-2-(carboxycyclopropyl)glycine	97	L-CCG-1	Glutamate	159.14
351	CNQX disodium	91	6-Cyano-7-nitroquinoxaline-2,3-dione	Glutamate	276.12
352	CX 546	97	1-(1,4-Benzodioxan-6-ylcarbonyl)piperidine	Glutamate	247.30
353	Chloro-IB-MECA	120	2-Chloro-N6-(3-iodobenzyl)-adenosine-5'-N-methyluronamide	Adenosine	544.74
354	WB-4101 hydrochloride	101	2-(2,6-Dimethoxyphenoxyethyl)aminomethyl-1,4-benzodioxane hydrochloride	Adrenoceptor	381.86
355	DNQX	100	6,7-Dinitroquinoxaline-2,3-dione	Glutamate	252.14
356	Dihydroouabain	56		Ion Pump	586.68
357	Dobutamine hydrochloride	98	(±)-4-[2-[[3-(4-Hydroxyphenyl)-1-methylpropyl]amino]ethyl]-1,2-benzenediol hydrochloride	Adrenoceptor	337.85
358	Dihydrokainic acid	94	2-Carboxy-4-isopropyl-3-pyrrolidineacetic acid	Glutamate	229.28
359	Decamethonium dibromide	98	Decamethylene bis(trimethylammonium bromide)	Cholinergic	418.30
360	P1,P4-Di(adenosine-5')tetraphosphate triammonium	75	Ap4A	Biochemistry	887.49
361	Debrisoquin sulfate	86	3,4-Dihydro-2(1H)-isoquinolinecarboximidamide sulfate; Ro 5-33071	Neurotransmission	448.55
362	2',3'-didehydro-3'-deoxythymidine	99	2',3'-Anhydrothymidine; d4T	Immune System	224.22
363	Droperidol	58	1-[1-[3-(p-Fluorobenzoyl)propyl]-1,2,3,6-tetrahydro-4-pyridyl]-2-benzimidazolinone	Dopamine	379.44
364	L-3,4-Dihydroxyphenylalanine methyl ester hydrochloride	90	Methyl L-DOPA hydrochloride	Dopamine	247.68
365	1,4-Dideoxy-1,4-imino-D-arabinitol	103	DAB; 2-Hydroxymethyl-3,4-pyrrolidinediol	Phosphorylation	169.61
366	2,4-Dinitrophenyl 2-fluoro-2-deoxy-beta-D-glucopyranoside	75		Biochemistry	348.24
367	D-ribofuranosylbenzimidazole	24	DRB; 5,6-Dichlorobenzimidazole riboside	Transcription	319.15
368	Dequalinium analog, C-14 linker	39	C14 Linker; DECA-14; Quinolinium	Phosphorylation	766.60
369	Diltiazem hydrochloride	107		Ca2+ Channel	450.99
370	Dextromethorphan hydrobromide monohydrate	102	d-3-Methoxy-N-methylmorphinan hydrobromide	Glutamate	352.32
371	SB 203186	84	1-Piperidinylethyl-1H-indole-3-carboxylate hydrochloride	Serotonin	308.81
372	Dihydroergotamine methanesulfonate	46		Serotonin	679.80
373	Diphenyleneiodonium chloride	36	[1,1'-Biphenyl]-2,2'-diyiodonium chloride	Nitric Oxide	314.55
374	Diphenhydramine hydrochloride	55		Histamine	291.82
375	2,3-Butanedione	88	Biacetyl; BDM	Cytoskeleton and ECM	86.09
376	N,N,N',N'-Tetramethylazodicarboxamide	93	Azodicarboxylic acid bis(dimethylamide); diamide	Cell Stress	172.19
377	(S)-3,5-Dihydroxyphenylglycine	93	S-DHPG	Glutamate	183.17
378	Dequalinium dichloride	73	1,1'-Decamethylenebis(4-aminoquinaldinium) dichloride	K+ Channel	527.59
379	Doxylamine succinate	85		Histamine	388.47
380	Desipramine hydrochloride	29		Adrenoceptor	302.85
381	trans-Dehydroandrosterone	114	5-Androsten-3beta-ol-17-one; DHEA	Hormone	288.43
382	5,5-Diphenylhydantoin	96	Phenytoin	Anticonvulsant	252.28
383	N^G,N^G-Dimethylarginine hydrochloride	106	asym-Dimethylarginine hydrochloride ADMA	Nitric Oxide	238.72
384	Clodronic acid	83	Cl2MDP; Clodronic acid disodium magnesium salt; DMDP	Cytoskeleton and ECM	288.86
385	Phenytoin sodium	77	5,5-Diphenylhydantoin sodium	Anticonvulsant	274.26
386	Doxepin hydrochloride	10		Adrenoceptor	315.85
387	S(-)-Pindolol	91		Adrenergic	248.33
388	(-)-alpha-Methylnorepinephrine	88	Levonordefrin; (-)-3,4-Dihydroxynorephedrine	Adrenoceptor	183.21
389	Dilazep hydrochloride	62	Cormelian	Adenosine	677.63
390	Dehydroisoandrosterone 3-sulfate sodium	94	Dehydroepiandrosterone 3-sulfate sodium	GABA	390.48
391	1,7-Dimethylxanthine	94	Paraxanthine	Adenosine	180.17
392	2,3-Dimethoxy-1,4-naphthoquinone	45	DMNQ	Cell Stress	218.21
393	Daphnetin	74	7,8-Dihydroxycoumarin	Phosphorylation	178.15
394	DM 235	101		Nootropic	246.31
395	5,5-Dimethyl-1-pyrroline-N-oxide	94	DMPO	Cell Stress	113.16
396	2',3'-dideoxycytidine	102	ddC	Immune System	211.22
397	Diacylglycerol Kinase Inhibitor II	74	R59949	Phosphorylation	489.59
398	Dihydrexidine hydrochloride	110	(±)-trans-10,11-Dihydroxy-5,6,6a,7,8,12b-hexahydrobenzo[a]phenanthridine hydrochloride	Dopamine	303.79
399	N-Methyldopamine hydrochloride	94	Epinine hydrochloride; Deoxyepinephrine hydrochloride	Dopamine	203.67
400	1,1-Dimethyl-4-phenyl-piperazinium iodide	87	DMPP	Cholinergic	318.20
401	Diacylglycerol kinase inhibitor I	10	R 59022	Phosphorylation	459.59
402	Disopyramide phosphate	104		K+ Channel	437.48
403	Demeclocycline hydrochloride	84	7-chloro-6-demethyltetracycline hydrochloride	Antibiotic	501.32
404	Diethylenetriaminepentaacetic acid	118	Pentetic acid; DTPA	Biochemistry	393.35
405	Diclofenac sodium	94	2-[(2,6-Dichlorophenyl)amino]benzeneacetic acid sodium	Prostaglandin	318.14
406	DL-erythro-Dihydrosphingosine	88	DL-Sphinganine	Phosphorylation	301.52
407	R-(-)-Desmethyldeprenyl hydrochloride	90	L-Nordeprenyl hydrochloride	Neurotransmission	209.72
408	2,2'-Bipyridyl	94	alpha,alpha'-Bipyridyl	Biochemistry	156.19

409	Disopyramide	95	alpha-Diisopropylaminoethyl-alpha-phenylpyridine-2-acetamide	Na+ Channel	339.48
410	Daidzein	47	7-Hydroxy-3-(4-hydroxyphenyl)-4H-1-benzopyran-4-one	Cell Cycle	254.24
411	Dubinidine	100		Anticonvulsant	275.31
412	Dicyclomine hydrochloride	91	2-(Diethylamino)ethyl 1-cyclohexylcyclohexane-1-carboxylate hydrochloride	Cholinergic	345.96
413	3,4-Dichloroisocoumarin	96	3,4-DCI	Biochemistry	215.04
414	DBO-83	100	3-(6-Chloro-3-pyridazinyl)-3,8-diazabicyclo[3.2.1]octane dihydrochloride	Cholinergic	297.62
415	7,7-Dimethyl-(5Z,8Z)-eicosadienoic acid	82	DEDA	Lipid	336.56
416	(±) trans-U-50488 methanesulfonate	74	trans-(±)-3,4-Dichloro-□N-methyl-N-[2-(1-pyrrolidinyl)-cyclohexyl]-benzeneacetamide methanesulfonate	Opioid	465.44
417	Dephostatin	111	2-(N-methyl-N-nitroso)hydroquinone	Phosphorylation	168.15
418	3',4'-Dichlorobenzamil	73	L-594,881	Ion Pump	425.11
419	3-deazaadenosine	59	4-Amino-1-(beta-D-ribofuranosyl)-1H-imidazo(4,5)-pyridine	Immune System	252.23
420	(Z)-Gugglesterone	91	trans-4,17(20)-Pregnadiene-3,16-dione	Lipid Signaling	312.46
421	Danazol	83		Hormone	337.47
422	N,N-Dihexyl-2-(4-fluorophenyl)indole-3-acetamide	93	FGIN-1-27	Benzodiazepine	436.62
423	(R,R)-cis-Diethyl tetrahydro-2,8-chrysenediol	40	(5R, 11R)-5,11-Diethyl-5,6,11,12-tetrahydro-2,8-chrysenediol	Hormone	320.44
424	SP600125	101	Anthrapyrazolone; 1,9-Pyrazoloanthrone	Phosphorylation	220.23
425	Diazoxide	115		K+ Channel	230.67
426	3,4-Dihydroxyphenylacetic acid	120	DOPAC	Dopamine	168.15
427	Dantrolene sodium	100	1-([5-(p-Nitrophenyl)furfurylidene]amino)hydantoin	Intracellular Calcium	336.24
428	DCEBIO	67	5,6-Dichloro-1-ethyl-1,3-dihydro- 2H-benzimidazol-2-one	K+ Channel	231.08
429	1-Deoxynojirimycin hydrochloride	116	DNM; 1,5-Dideoxy-1,5-imino-D-sorbitol hydrochloride	Biochemistry	199.64
430	L-3,4-Dihydroxyphenylalanine	91	L-DOPA; Levodopa	Dopamine	197.19
431	Dipyridamole	112		Adenosine	504.64
432	Doxazosin mesylate	86	1-(4-amino-6,7-dimethoxy-2-quinazolinyl)-4-[4-(1,4-benzodioxan-2-yl)carpiperazin-1-yl)]-6,7-dimethoxyquinazoline mesylate	Adrenoceptor	547.59
433	Doxycycline hydrochloride	103		Antibiotic	480.91
434	6,7-ADTN hydrobromide	118	(±)-2-Amino-6,7-dihydroxy-1,2,3,4-tetrahydro-naphthalene hydrobromide	Dopamine	260.13
435	R(-)-Apocodeine hydrochloride	98	R(-)-10-Methoxy-11-hydroxyaporphine hydrochloride	Dopamine	317.82
436	R(-)-Propylnorapomorphine hydrochloride	99	R(-)-NPA hydrochloride	Dopamine	331.85
437	R(-)-2,10,11-Trihydroxyaporphine hybrobromide	71	R(-)-2-Hydroxyapomorphine hydrobromide	Dopamine	364.24
438	R(-)-2,10,11-Trihydroxy-N-propylnoraporphine hydrobromide	88	R(-)-TNPA HBr; R(-)-2-OH-NPA hydrobromide	Dopamine	392.30
439	Dipropyldopamine hydrobromide	106		Dopamine	318.26
440	(+)-Butaclamol hydrochloride	14		Dopamine	397.99
441	R(-)-N-Allylnorapomorphine hydrobromide	87		Dopamine	374.28
442	Amfonelic acid	96	7-Benzyl-1-ethyl-1,4-dihydro-4-oxo-1,8-naphthyridine-3-carboxylic acid	Dopamine	308.34
443	Icilin	87	AG-3-5; 1-(2-Hydroxyphenyl)-4-(3-nitrophenyl)-1,2,3,6-tetrahydropyrimidin-2-one	Neurotransmission	311.30
444	(±)-SKF-38393 hydrochloride	101	(±)-1-Phenyl-2,3,4,5-tetrahydro-(1H)-3-benzazepine-7,8-diol hydrochloride	Dopamine	291.78
445	GBR-12909 dihydrochloride	59	1-[2-[bis(4-Fluorophenyl)methoxy]ethyl]-4-[3-phenylpropyl]piperazine dihydrochloride	Dopamine	523.50
446	R(+)-SCH-23390 hydrochloride	17	R(+)-7-Chloro-8-hydroxy-3- methyl-1-phenyl-2,3,4,5-tetrahydro-1H-3-benzazepine hydrochloride	Dopamine	324.25
447	(±)-DOI hydrochloride	86	(±)-2,5-Dimethoxy-4-iodoamphetamine hydrochloride	Serotonin	357.62
448	(±)-2,3-Dichloro-alpha-methylbenzylamine hydrochloride	84	DCMB hydrochloride; LY-78335	Neurotransmission	226.53
449	4-DAMP methiodide	97	4-Diphenylacetoxy-N-methylpiperidine methiodide	Cholinergic	451.35
450	1,3-Dipropyl-7-methylxanthine	123		Adenosine	250.30
451	Domperidone	98		Dopamine	425.92
452	Propofol	94		Cholinergic	178.28
453	Dextrorphan D-tartrate	107	(+)-3-Hydroxy-N-methylmorphinan D-tartrate	Glutamate	407.47
454	R(+)-Butylindazone	97	R(+)-DIOA	Ion Pump	399.32
455	DPMA	110	PD-125944	Adenosine	521.58
456	3,5-Dinitrocatechol	95	OR-486	Neurotransmission	200.11
457	N,N-Dipropyl-5-carboxamidotryptamine maleate	106		Serotonin	403.48
458	6,7-Dichloroquinoxaline-2,3-dione	129	DCQX	Glutamate	231.04
459	3,7-Dimethyl-I-propargylxanthine	101	DMPX	Adenosine	218.22
460	5,7-Dichlorokynurenic acid	102	5,7-Dichloro-4-hydroxyquinoline-2-carboxylic acid	Glutamate	258.06
461	4-Diphenylacetoxy-N-(2-chloroethyl)piperidine hydrochloride	99	4-DAMP mustard hydrochloride	Cholinergic	394.34
462	1,10-Diaminodecane	99	DA10; Decamethylenediamine	Glutamate	172.32
463	Dihydro-beta-erythroidine hydrobromide	103	3beta-1,6-Didehydro-14,17-dihydro-3-methoxy-16(15H)-oxaerythrinan-15-one hydrobromide	Cholinergic	356.26
464	(R)-(-)-DOI hydrochloride	69	(-)-2,5-Dimethoxy-4-iodoamphetamine hydrochloride	Serotonin	357.62
465	Dihydroergocristine methanesulfonate	32		Dopamine	707.85
466	2,6-Diamino-4-pyrimidinone	114	DAHP; 2,4-Diamino-6-hydroxypyrimidine	Phosphorylation	126.12
467	DL-alpha-Difluoromethylornithine hydrochloride	94	DFMO hydrochloride	Angiogenesis	218.63
468	4',4''-Difluor-3alpha-(diphenylmethoxy)tropane hydrochloride	123		Dopamine	379.88
469	S(-)-DS 121 hydrochloride	94	S(-)-3-(1-Propyl-3-piperidinyl)-benzonitrile hydrochloride	Dopamine	264.80
470	Vanillic acid diethylamide	101	Ethamivan	Vanilloid	223.27
471	Epibestatin hydrochloride	107	([2R,3R]-3-Amino-2-hydroxy-4-phenylbutanoyl)-L-leucine hydrochloride	Biochemistry	344.84
472	Etodolac	98	1,8-Diethyl-1,3,4,9-tetrahydropyrano[3,4-b]indole-1-acetic acid	Prostaglandin	287.36
473	Enoximone	103	1,3-Dihydro-4-methyl-5-[4-methylthiobenzoyl]-2H-imidazol-2-one	Cyclic Nucleotides	248.31
474	Etoposide	90	Lastet	Apoptosis	588.57
475	ET-18-OCH3	113	3,5,9-Trioxa-4-phosphaheptacosan-1-aminium	Lipid	523.74
476	Etazolate hydrochloride	136	SQ 20,009; 1-Ethyl-4-[(1-methylethylidene)hydrazino]-1H-pyrazolo[3,4-b]pyridine-5-carboxylic acid ethyl ester hydrochloride	Adenosine	325.80
477	7-Cyclopentyl-5-(4-phenoxy)phenyl-7H-pyrrolo[2,3-d]pyrimidin-4-ylamine	76		Phosphorylation	370.46

478	Emetine dihydrochloride hydrate	14		Apoptosis	553.58
479	5'-N-Ethylcarboxamidoadenosine	141	NECA	Adenosine	308.30
480	E-64	112	L-trans-3-Carboxyoxiran-2-carbonyl-L-leucylagmatine	Biochemistry	357.41
481	SB 415286	48	3-[(3-Chloro-4-hydroxyphenyl)amino]-4-(2-nitrophenyl)-1H-pyrrol-2,5-dione	Phosphorylation	359.73
482	S-Ethylisothiourea hydrobromide	104	2-Ethyl-2-thiopseudourea hydrobromide	Nitric Oxide	185.09
483	(-)-Ephedrine hemisulfate	60		Adrenoceptor	428.55
484	Edrophonium chloride	118	Ethyl(m-hydroxyphenyl)dimethylammonium chloride	Cholinergic	201.70
485	Efaroxan hydrochloride	74	RX 821037A	Imidazoline	252.75
486	Ellipticine	98	5,11-Dimethyl-6H-pyrido[4,3-b]carbazole	Cell Cycle	246.31
487	Ebselen	102	2-Phenyl-1,2-benzisoselenazol-3(2H)-one	Leukotriene	274.18
488	rac-2-Ethoxy-3-hexadecanamido-1-propylphosphocholine	42		Phosphorylation	522.71
489	rac-2-Ethoxy-3-octadecanamido-1-propylphosphocholine	65		Phosphorylation	550.77
490	N-Ethylmaleimide	87	NEM	Biochemistry	125.13
491	(-)-Epinephrine bitartrate	136	Adrenaline bitartrate	Adrenoceptor	333.30
492	Ethylene glycol-bis(2-aminoethylether)-N,N,N',N'-tetraacetic acid	121	EGTA; Egtazic acid	Biochemistry	380.35
493	(±)-Epinephrine hydrochloride	130	(±)-Adrenalin hydrochloride	Adrenoceptor	219.67
494	Ethosuximide	146	2-Ethyl-2-methylsuccinimide	Anticonvulsant	141.17
495	Endothall	117	7-Oxabicyclo[2.2.1]heptane-2,3-dicarboxylic acid	Phosphorylation	186.17
496	Emodin	49		Phosphorylation	270.24
497	(-)-Physostigmine	93	Eserine	Cholinergic	275.35
498	NBI 27914	90	5-Chloro-4-(N-(cyclopropyl)methyl-N-propylamino)-2-methyl-6-(2,4,6-trichlorophenyl)-aminopyridine	Neurotransmission	434.20
499	beta-Estradiol	112	Dihydrofolliculin	Hormone	272.39
500	Estrone	91	Folliculin	Hormone	270.37
501	Methyl beta-carboline-3-carboxylate	118	beta-CCM	Benzodiazepine	226.24
502	N-Methyl-beta-carboline-3-carboxamide	80	FG-7142	GABA	225.25
503	Methyl 6,7-dimethoxy-4-ethyl-beta-carboline-3-carboxylate	79	DMCM	Benzodiazepine	314.34
504	(-)-Eseroline fumarate	56		Cholinergic	334.38
505	S-(-)-Eticlopride hydrochloride	85	FLB 131	Dopamine	377.31
506	(S)-ENBA	112	PD-126280; ((2S)-N6-[2-endo-Norbornyl]adenosine	Adenosine	361.40
507	erythro-9-(2-Hydroxy-3-nonyl)adenine hydrochloride	128	EHNA hydrochloride	Adenosine	313.83
508	Ergocristine	100	12'-Hydroxy-2'-(1-methyl-ethyl)-5'-(phenylmethyl)ergotaman-3'6'18-trione	Dopamine	609.73
509	Felbamate	113	2-Phenyl-1,3-propanediol dicarbamate	Glutamate	238.25
510	Fusidic acid sodium	131	Fusidin	Cell Cycle	538.71
511	Fenoterol hydrobromide	136	2-(3,5-Dihydroxyphenyl)-2-hydroxy-2'-(4-hydroxyphenyl)-1'-methyldiethylamine hydrobromide	Adrenoceptor	384.27
512	S-(+)-Fluoxetine hydrochloride	77		Serotonin	345.80
513	R-(-)-Fluoxetine hydrochloride	79		Serotonin	345.80
514	Fluvoxamine maleate	114	(E)-5-Methoxy-1-[4-(trifluoromethyl)phenyl]-1-pentanone-O-(2-aminoethyl)oxime maleate	Serotonin	434.42
515	1-(4-Fluorobenzyl)-5-methoxy-2-methylindole-3-acetic acid	103		Multi-Drug Resistance	327.36
516	Furegrelate sodium	146	5-(3-Pyridinylmethyl)benzofurancarboxylic acid sodium	Phosphorylation	275.24
517	Fiduxosin hydrochloride	69	(3-[4-((3alphaR,9betaR)-cis-9-methoxy-1,2,3,3a,4,9b-hexahydro-[1]-benzopyrano[3,4-c]pyrrol-2-yl)butyl]-8-phenyl-pyrazino-[2',3':4,5]thi-eno [3,2-d]pyrimid	Adrenoceptor	592.12
518	Furosemide	129		Ion Pump	330.75
519	p-Fluoro-L-phenylalanine	97	4-Fluoro-L-phenylalanine	Neurotransmission	183.18
520	Fluphenazine dihydrochloride	18		Dopamine	510.45
521	Fenofibrate	95	2-[4-(4-Chlorobenzoyl)phenoxy]-2-methylpropanoic acid 1-methyl-ethyl ester	Transcription	360.84
522	Fenspiride hydrochloride	98	8-(2-Phenylethyl)-1-oxa-3,8-diazaspiro[4.5]decan-2-one hydrochloride	Adrenoceptor	296.80
523	Flumazenil	124	Ro 15-1788	Benzodiazepine	303.30
524	Foliosidine	190		Anticonvulsant	307.35
525	Fusaric acid	152	5-Butyl-2-pyridinecarboxylic acid	Dopamine	179.22
526	5-Fluorouracil	156	5-FU	Cell Cycle	130.08
527	Flecainide acetate	111	N-(2-Piperidylmethyl)-2,5-bis-(2,2,2-trifluoroethoxy)benzamide acetate	Na+ Channel	474.40
528	Fenoldopam bromide	112	SKF-82526	Dopamine	386.68
529	Forskolin	46		Cyclic Nucleotides	410.51
530	Famotidine	115	N'-(Aminosulfonyl)-3-([2-(diaminomethyleneamino)-4-thiazolyl]methylthio)propanamidine	Histamine	337.45
531	FSCPX	97	8-Cyclopentyl-N3-[3-(4-(fluorosulfonyl)benzoyloxy)propyl]-N1-propylxanthine	Adenosine	506.56
532	Farnesylthiosalicylic acid	120	FTS	G protein	358.55
533	Flunarizine dihydrochloride	71	1-[bis(4-fluorophenyl)methyl]-4-(3-phenyl-2-propenyl)-piperazine dihydrochloride	Ion Pump	477.43
534	5-fluoro-5'-deoxyuridine	111	5'dFUrd	DNA Metabolism	246.20
535	Flupirtine maleate	109	2-amino-6-[[(4-fluorophenyl)methyl]amino]-3-pyridinyl]-carbamic acid, ethyl ester maleate	Glutamate	420.40
536	Flutamide	99	2-Methyl-N-(4-nitro-3-[trifluoromethyl]phenyl)propanamide	Hormone	276.22
537	Fexofenadine hydrochloride	87	Terfenidine carboxylate, MDL 16455	Histamine	501.67
538	Formoterol	91	(R*,R*)-N-[2-hydroxy-5-[1-hydroxy-2-[[2-(4-methoxyphenyl)-1-methylethyl]amino]ethyl]phenyl]formamide	Adrenoceptor	804.90
539	Felodipine	96	Plendil	Ca2+ Channel	384.26
540	Fluspirilene	53	R 6218	Dopamine	475.59
541	cis-(Z)-Flupenthixol dihydrochloride	12	(Z)-4-[3-[2-(Trifluoromethyl)-9H-thioxanthen-9-ylidene]propyl]-1-piperazine-ethanol dihydrochloride	Dopamine	507.45
542	Furafylline	120	3-(2-Furanylmethyl)-3,7-dihydro-1,8-dimethyl-1H-purine-2,6-dione	Biochemistry	260.25
543	FPL 64176	86	2,5-Dimethyl-4-[2-(phenylmethyl)benzoyl]-1H-pyrrole-3-carboxylic acid methyl ester	Ca2+ Channel	347.42
544	Fluoxetine hydrochloride	118	Prozac; LY-110,140 hydrochloride	Serotonin	345.80
545	N-(3,3-Diphenylpropyl)glycinamide	105	N20C	Glutamate	268.36
546	Glibenclamide	118	Glyburide	K+ Channel	494.01

547	GW2974	68	N4-(1-Benzyl-1H-indazol-5-yl)-N6,N6-dimethyl-pyrido[3,4-d]pyrimidine4-4,6-diamine	Phosphorylation	395.47
548	Guanfacine hydrochloride	112	N-(aminoiminomethyl)-2,6-dichloro-benzeneacetamide hydrochloride	Adrenoceptor	282.56
549	L-Glutamic acid hydrochloride	93	S(+)-1-Aminopropane-1,3-dicarboxylic acid hydrochloride	Glutamate	183.59
550	Ganciclovir	92		Cell Cycle	255.24
551	L-Glutamine	115	S(+)-Glutamic acid 5-amide	Glutamate	146.15
552	Guanidinyl-naltrindole di-trifluoroacetate	120	GNTI di-trifluoroacetate	Opioid	699.61
553	Guanidinoethyl disulfide dihydrobromide	105	GED dihydrobromide	Nitric Oxide	398.19
554	GW1929	131	N-(2-Benzoylphenyl)-O-[2-(methyl-2-pyridinylamino)ethyl]-L-tyrosine; N-(2-Benzoyl phenyl)-L-tyrosine	Transcription	495.58
555	GW5074	25	3-(3, 5-Dibromo-4-hydroxybenzylidine-5-iodo-1,3-dihydro-indol-2-one)	Phosphorylation	520.95
556	Genistein	67	5,7-Dihydroxy-3-(4-hydroxyphenyl)-4H-1-benzopyran-4-one	Phosphorylation	270.24
557	GW7647	50	2-(4-(2-(1-Cyclohexanebutyl)-3-cyclohexylureido)ethyl)phenylthio)-2-methylpropionic acid	Transcription	502.77
558	alpha-Guanidinoglutaric acid	121	GGA	Nitric Oxide	189.17
559	Gallamine triethiodide	120		Cholinergic	891.54
560	GBR-12935 dihydrochloride	61	1-[2-(Diphenylmethoxy)ethyl]-4-(3-phenylpropyl)-piperazine dihydrochloride	Dopamine	487.52
561	Isoguvacine hydrochloride	71	1,2,3,6-Tetrahydro-4-pyridinecarboxylic acid hydrochloride	GABA	163.61
562	Guvacine hydrochloride	94		GABA	163.61
563	(±)-AMPA hydrobromide	101	(±)-alpha-Amino-3-hydroxy-5-methylisoxazole-4-propionic acid hydrobromide	Glutamate	267.08
564	Muscimol hydrobromide	130	3-Hydroxy-5-aminomethylisoxazole hydrobromide	GABA	195.02
565	Guanabenz acetate	156	WY-8678	Adrenoceptor	291.14
566	gamma-D-Glutamylaminomethylsulfonic acid	109	GAMS	Glutamate	240.24
567	Glipizide	103		K+ Channel	445.54
568	GYKI 52466 hydrochloride	90	1-(4-Aminophenyl)-4-methyl-7,8-methylenedioxy-5H-2,3-benzodiazepine hydrochloride	Glutamate	329.79
569	GYKI 52895	71	1-(4-Aminophenyl)-4-methyl-7,8- methylenedioxy-3,4-dihydro-5H-2,3-benzodiazepine hydrochloride	Dopamine	295.34
570	GR-89696 fumarate	79	4-[(3,4-Dichlorophenyl)acetyl]-3- (1-pyrrolidinylmethyl)-1-piperazinecarboxylic acid methyl ester fumarate	Opioid	530.41
571	Gabapentin	112	1-(Aminomethyl)-cyclohexaneacetic acid	Anticonvulsant	171.24
572	DL-Homatropine hydrobromide	138	Tropine mandelate hydrobromide	Cholinergic	356.26
573	(±)-Vanillylmandelic acid	115	(±)-4-Hydroxy-3-methoxymandelic acid	Adrenoceptor	198.18
574	6-Hydroxymelatonin	101	3-(N-Acetylaminoethyl)-6-hydroxy-5-methoxyindole	Melatonin	248.28
575	Hexamethonium bromide	91	Hexane-1,6-bis(trimethylammonium bromide)	Cholinergic	362.19
576	4-Hydroxy-3-methoxyphenylacetic acid	82	Homovanillic acid; HVA	Dopamine	182.18
577	MHPG piperazine	104	MOPEG piperazine	Adrenoceptor	454.52
578	Hypotaurine	89	2-Aminoethanesulfinic acid	Cell Stress	109.15
579	Haloperidol	56		Dopamine	375.87
580	Hydralazine hydrochloride	100	1-Hydrazinophthalazine hydrochloride	Neurotransmission	196.64
581	4-Imidazolemethanol hydrochloride	114	4-(Hydroxymethyl)imidazole hydrochloride	Histamine	134.57
582	Hexamethonium dichloride	94	N,N,N,N',N',N'-Hexamethyl-1,6-hexanediaminium dichloride	Cholinergic	273.29
583	Hydrocortisone 21-hemisuccinate sodium	136	Cortisol 21-hemisuccinate sodium	Hormone	484.53
584	6-Hydroxy-DL-DOPA	101	2,5-Dihydroxy-DL-tyrosine	Adrenoceptor	213.19
585	DL-threo-beta-hydroxyaspartic acid	118	threo-2-Amino-3-hydroxysuccinic acid	Glutamate	149.10
586	4-Methoxy-3-hydroxyphenethylamine hydrochloride	130	4-O-Methyldopamine hydrochloride	Dopamine	203.67
587	Hydroxytacrine maleate	86	HP-029	Cholinergic	330.34
588	Hydrocortisone	78	Cortisol	Hormone	362.47
589	Lithium Chloride	86		Neurotransmission	42.39
590	Hydrochlorothiazide	106	6-Chloro-3,4-dihydro-2H-1,2,4-benzothiadiazine-7-sulfonamide 1,1-dioxide	Biochemistry	297.74
591	SB 218795	98	(-)-(R)-N-(alpha-methoxycarbonylbenzyl)-2-phenylquinoline-4-carboxamide	Neurotransmission	396.45
592	Hispidin	81	6-(3,4-dihydroxystyrl)-4-hydroxy-2-pyrone	Phosphorylation	246.22
593	17alpha-hydroxyprogesterone	83	17alpha-Hydroxy-4-pregnene-3,20-dione	Hormone	330.47
594	1,3,5-tris(4-hydroxyphenyl)-4-propyl-1H-pyrazole	73	PPT	Hormone	386.45
595	1-(4-Hydroxybenzyl)imidazole-2-thiol	63		Dopamine	206.27
596	Histamine dihydrochloride	95	1H-Imidazole-4-ethanamine dihydrochloride	Histamine	184.07
597	Harmane	60	Aribine	Imidazoline	182.23
598	NG-Hydroxy-L-arginine acetate	94	NOHA acetate	Nitric Oxide	250.26
599	Retinoic acid p-hydroxyanilide	88	N-(4-Hydroxyphenyl)retinamide	Cell Cycle	391.56
600	HE-NECA	99	2-Hexynyl-5'-ethylcarboxamidoadenosine	Adenosine	388.43
601	L-Histidine hydrochloride	91	S(+)-alpha-Amino-1H-imidazole-4-propanoic acid hydrochloride	Histamine	191.62
602	(±)-8-Hydroxy-DPAT hydrobromide	102	(±)-8-Hydroxy-dipropylaminotetralin hydrobromide	Serotonin	328.30
603	Dopamine hydrochloride	106	3-Hydroxytyramine hydrochloride	Dopamine	189.64
604	Hydroxyurea	122		DNA Metabolism	76.06
605	(+)-Hydrastine	122	(+)-beta-Hydrastine 1(S),9(R)	GABA	383.40
606	(±)-7-Hydroxy-DPAT hydrobromide	62	(±)-7-OH-DPAT HBr; (±)-Hydroxy-dipropylaminotetralin hydrobromide	Dopamine	328.30
607	MHPG sulfate potassium	131	4-Hydroxy-3-methoxyphenylglycol-4-sulfate potassium	Adrenoceptor	302.35
608	5-Hydroxyindolacetic acid	85	5-HIAA	Serotonin	191.19
609	L-Hyoscyamine	75	[3(S)-endo]-alpha-(Hydroxymethyl)benzeneacetic acid 8-methyl-8-azabicyclo[3.2.1]oct-3-yl ester	Cholinergic	289.38
610	Hydroquinone	89	1,4-Benzenediol	Leukotriene	110.11
611	BU99006	104	2-(Imidazolin-2-yl)-5-isothiocyanatobenzofuran	Imidazoline	243.29
612	3-Hydroxybenzylhydrazine dihydrochloride	104	NSD-1015; alpha-Hydrazino-m-cresol dihydrochloride	Biochemistry	211.09
613	Serotonin hydrochloride	110	5-HT hydrochloride	Serotonin	212.68
614	L-165,041	116	4-[3-(4-Acetyl-3-hydroxy-2-propylphenoxy)propoxy]phenoxyacetic acid	Lipid Signaling	402.45
615	5-Hydroxy-L-tryptophan	126	S(+)-1-alpha-Amino-5-hydroxyindole-3-propionic acid	Serotonin	220.23

616	Hydroxylamine hydrochloride	118		Neurotransmission	69.49
617	4-Hydroxybenzhydrazide	78	4-Hydroxybenzoylhydrazine	Biochemistry	152.15
618	Hemicholinium-3	106		Cholinergic	574.36
619	HA-1004 hydrochloride	89	N-(2-Guanidinoethyl)-5-isoquinolinesulfonamide hydrochloride	Phosphorylation	329.81
620	H-7 dihydrochloride	38	1-(5-Isoquinolinesulfonyl)-2-methylpiperazine dihydrochloride	Phosphorylation	364.30
621	Hexahydro-sila-difenidol hydrochloride, p-fluoro analog	73	p-F-HHSiD hydrochloride	Cholinergic	386.03
622	Histamine, R(-)-alpha-methyl-, dihydrochloride	127	R(-)-alpha-Methylhistamine dihydrochloride	Histamine	198.10
623	5-hydroxydecanoic acid sodium	126		K+ Channel	210.25
624	R-(+)-8-Hydroxy-DPAT hydrobromide	89	(+)-8-Hydroxy-2-(dipropylamino)tetralin hydrobromide	Serotonin	328.30
625	R-(+)-7-Hydroxy-DPAT hydrobromide	85	R(+)-7-Hydroxy-dipropylaminotetralin hydrobromide	Dopamine	328.30
626	GR 125487 sulfamate salt	87	[1-[2-[(Methylsulfonyl)amino]ethyl]-4-piperidinyl]methyl-5-fluoro-2-methoxy-1H-indole-3-carboxylate	Serotonin	524.59
627	IEM-1460	97	1-Trimethylammonio-5-(1-adamantanemethylammonio)pentane dibromide	Glutamate	428.30
628	Ibudilast	136	KC-404	Cyclic Nucleotides	230.31
629	Imidazole-4-acetic acid hydrochloride	122	I4AA	GABA	162.58
630	Indirubin-3'-oxime	23	Indirubin-3'-monoxime	Phosphorylation	277.28
631	NSC 95397	17	2,3-bis[(2-Hydroxyethyl)thio]-1,4-naphthoquinone	Phosphorylation	310.39
632	Imazodan	92	CI 914	Cyclic Nucleotides	240.27
633	Iodoacetamide	84		Biochemistry	184.96
634	HA-100	71	1-(5-Isoquinolinesulfonyl)piperazine hydrochloride	Phosphorylation	313.81
635	Ipratropium bromide	118	Atropine isopropyl bromide	Cholinergic	412.37
636	Idarubicin	17	Idamycin	DNA Metabolism	497.51
637	2-Iodomelatonin	113	N-Acetyl-2-iodo-5-methoxytryptamine	Melatonin	358.18
638	KN-62	104	1-[N,O-bis(5-Isoquinolinesulfonyl)-N-methyl-L-tyrosyl]-4-phenylpiperazine	Phosphorylation	721.86
639	IMID-4F hydrochloride	109	2-[N-(2,6-dichlorophenyl)-N-(4-fluorobenzyl)amino]-2-imidazoline hydrochloride	K+ Channel	374.68
640	R(-)-Isoproterenol (+)-bitartrate	118		Adrenoceptor	361.35
641	ML-7	85	1-(5-Iodonaphthalene-1-sulfonyl)-1H-hexahydro-1,4-diazepine hydrochloride	Phosphorylation	452.74
642	(±)-Ibotenic acid	105	(±)-alpha-Amino-3-hydroxy-5-isoxazoleacetic acid	Glutamate	158.11
643	Ifenprodil tartrate	64	alpha-(4-Hydroxyphenyl)-beta-(4-benzylpiperidin-1-yl)-beta-methylethanol tartrate	Glutamate	801.00
644	Isotharine mesylate	115	4-[1-Hydroxy-2-[(1-methylethyl)amino]butyl]-1,2-benzenediol mesylate	Adrenoceptor	335.42
645	Isoliquiritigenin	53	2',4,4'-Trihydroxychalcone	Cyclic Nucleotides	256.26
646	(±)-Ibuprofen	98	alpha-Methyl-4-(isobutyl)phenylacetic acid	Prostaglandin	206.29
647	IIK7	96	N-Butanoyl 2-(9-methoxy-6H-isoindolo[2,1-a]indol-11-yl)ethanamine	Melatonin	348.45
648	(±)-Isoproterenol hydrochloride	124		Adrenoceptor	247.72
649	3-Isobutyl-1-methylxanthine	129	IBMX	Adenosine	222.25
650	Idazoxan hydrochloride	103	RX 781094	Imidazoline	240.69
651	1-(5-Isoquinolinylsulfonyl)-3-methylpiperazine dihydrochloride	100	Iso-H-7	Phosphorylation	364.30
652	(-)-Isoproterenol hydrochloride	119	(-)-Isoprenaline hydrochloride	Adrenoceptor	247.72
653	1-(5-Isoquinolinylsulfonyl)-2-methylpiperazine dihydrochloride	29	H-7 dihydrochloride	Phosphorylation	364.30
654	Indomethacin	100		Prostaglandin	357.80
655	Imipramine hydrochloride	4		Serotonin	316.88
656	Isoxanthopterin	106	2-Amino-4,7-dihydroxypteridine	Cell Stress	179.14
657	Iproniazid phosphate	97		Neurotransmission	277.22
658	S(+)-Isoproterenol (+)-bitartrate	122		Adrenoceptor	361.35
659	L-N6-(1-Iminoethyl)lysine hydrochloride	101	L-NIL	Nitric Oxide	223.70
660	3-Iodo-L-tyrosine	100	S(-)-3-Iodo-4-hydroxyphenylalanine	Neurotransmission	307.09
661	L-N5-(1-Iminoethyl)ornithine hydrochloride	102	L-NIO	Nitric Oxide	209.68
662	Ivermectin	101	MK-933	Cholinergic	875.12
663	Imiloxan hydrochloride	96	RS 21361	Adrenoceptor	280.76
664	CR 2945	101	Itriglumide	Cholecystokinin	548.66
665	m-Iodobenzylguanidine hemisulfate	96	MIBG	Apoptosis	648.26
666	S(+)-Ibuprofen	102	S-(+)-2-(4-Isobutylphenyl)propionic acid	Prostaglandin	206.29
667	p-Iodoclonidine hydrochloride	83	2-[(2,6-Dichloro-4-iodophenyl)imino]imidazoline hydrochloride	Adrenoceptor	392.46
668	R(+)-IAA-94	100	R(+)-Methylindazone; Indanyloxyacetic acid 94	Cl- Channel	357.24
669	Indatraline hydrochloride	54	Lu 19-005	Dopamine	328.67
670	Iofetamine hydrochloride	90	N-Isopropyl-p-iodoamphetamine hydrochloride	Neurotransmission	339.65
671	ICI 204,448 hydrochloride	97		Opioid	501.84
672	ICI 118,551 hydrochloride	92	(±)-1-[2,3-(Dihydro-□7-methyl-1H-inden-4-yl)oxy]-3-[(1-methylethyl)amino]-2-butanol hydrochloride	Adrenoceptor	313.87
673	Imetit dihydrobromide	91	S-[2-(Imidazol-4-yl)ethyl]isothiourea dihydrochloride	Histamine	332.06
674	1,5-Isoquinolinediol	92	1,5-Dihydroxyisoquinoline	Apoptosis	161.16
675	S(-)-IBZM	85	Iodobenzamide	Dopamine	404.25
676	IB-MECA	105	1-Deoxy-1-[6-[[(3-iodophenyl) methyl]amino]-9H-purin-9-yl]-N-methyl-beta-D-ribofuranuronamide	Adenosine	510.29
677	Indomethacin morpholinylamide	89	BML-190	Cannabinoid	426.90
678	3-(1H-Imidazol-4-yl)propyl di(p-fluorophenyl)methyl ether hydrochloride	52		Histamine	364.83
679	Isonipecotic acid	94	4-Piperidine carboxylic acid	GABA	129.16
680	JWH-015	73	(2-Methyl-1-propyl-1H-indol-3-yl)-1-naphthalenylmethanone	Cannabinoid	327.43
681	JL-18	2	8-Methyl-6-(4-methyl-1-piperazinyl)-11H-pyrido[2,3-b][1,4]benzeodiazepine	Dopamine	307.40
682	Kainic acid	98	2-Carboxy-3-carboxymethyl-4-isopropenylpyrrolidine	Glutamate	213.24
683	Ketoconazole	85	cis-1-Acetyl-4-[4-[[2-(2,4- dichlorophenyl)-2-(1H-imidazol-1-ylmethyl)-1,3-dioxolan-4-yl]methoxy]phenyl]-piperazine	Multi-Drug Resistance	531.44
684	Ketorolac tris salt	98	Toradol	Prostaglandin	376.41

685	Ketoprofen	101	2-(3-Benzoylphenyl)propionic acid	Prostaglandin	254.29
686	K 185	80	N-Butanoyl 2-(5,6,7-trihydro-11-methoxybenzo[c]cyclohept[2,1-a]indol-13-yl)ethanamine	Melatonin	376.50
687	Ketotifen fumarate	4		Histamine	425.51
688	Kynurenic acid	99	4-Hydroxyquinoline-2-carboxylic acid	Glutamate	189.17
689	Kenpaullone	17	NSC 664704	Phosphorylation	327.18
690	Karakoline	101		Cholinergic	377.53
691	L-701,324	90	7-Chloro-4-hydroxy-3-(3-phenoxy)phenylquinolin-2[1H]-one	Glutamate	363.80
692	loxoprofen	92	Koloxo	Prostaglandin	246.31
693	Labetalol hydrochloride	83	2-Hydroxy-5-(1-hydroxy-2-[(1-methyl-3-phenylpropyl)amino]ethyl)benzamide hydrochloride	Adrenoceptor	364.88
694	L-162,313	99	(5,7-dimethyl-2-ethyl-3-[[4-[2(n-butyloxycarbonylsulfonamido)-5-isobutyl-3-thienyl]phenyl]methyl]imidazo[4,5,6]pyridine	Neurotransmission	582.79
695	Lidocaine N-methyl hydrochloride	92	QX-222	Na+ Channel	256.78
696	beta-Lapachone	14		Apoptosis	242.28
697	LY-367,265	29	1-[2-[4-(6-fluoro-1H-indol-3-yl)-3,6-dihydro-1(2H)-pyridinyl]ethyl]-5,6-dihydro-1H,4H-[1,2,5]thiadiazolo[4.3.2-ij]quinoline-2,2-dioxide	Serotonin	452.55
698	LY-310,762 hydrochloride	19		Serotonin	430.95
699	L-368,899	94	1-((7,7-Dimethyl-2(S)-(2(S)-amino-4-(methylsulfonyl)butyramido)bicyclo[2,2,1]heptan-1(S)-yl)methylsulfonyl)-4-(2-methylphenyl)piperazine hydrochloride	Neurotransmission	591.24
700	Lomefloxacin hydrochloride	90		Antibiotic	387.82
701	Lamotrigine	87	GI 267119X; 6-(2,3-dichlorophenyl)-1,2,4-triazine-3,5-diamine	Anticonvulsant	256.10
702	alpha-Lobeline hydrochloride	89	(-)-Lobeline hydrochloride	Cholinergic	373.93
703	Loperamide hydrochloride	83		Opioid	513.51
704	Lonidamine	90	Diclondazolic acid	Cell Stress	321.17
705	Leflunomide	97	5-Methylisoxazole-4-(4-trifluoromethylcarboxanilide)	Immune System	270.21
706	VER-3323 hemifumarate salt	82	(S)-1-(6-Bromo-2,3-dihydroindol-1-yl)-2-propylamine hemifumarate salt	Serotonin	626.39
707	Lidocaine hydrochloride	86		Na+ Channel	270.81
708	Lidocaine N-ethyl bromide quaternary salt	85	QX-314	Na+ Channel	343.31
709	L-Leucinethiol, oxidized dihydrochloride	81	Dithiobis(2-amino-4-methylpentane)	Biochemistry	337.42
710	LE 300	1	7-Methyl-6,7,8,9,14,15-hexahydro-5H-benz[d]indolo[2,3-g]azecine	Dopamine	290.41
711	Lansoprazole	91		Ion Pump	369.37
712	L-687,384 hydrochloride	87	1'-Benzyl-3,4-dihydrospiro[naphthalene-1-(2H),4'-piperidine] hydrochloride	Opioid	327.90
713	LFM-A13	98	alpha-Cyano-beta-hydroxy-beta-methyl-N-(2,5-dibromophenyl)propenamide	Phosphorylation	360.01
714	Luteolin	87	3',4',5,7-Tetrahydroxyflavone	Cell Stress	286.24
715	L-655,240	73	3-[1-(4-Chlorobenzyl)-5-fluoro-3-methyl-indol-2-yl]-2,2-dimethyl propanoic acid	Thromboxane	373.86
716	Loratadine	88	4-(8-chloro-5,6-dihydro-11H-benzo[5,6]cycloheptal[1,2-b]pyridin-11-ylidene-1-piperidinecarboxylic acid ethyl ester	Histamine	382.89
717	(-)-Tetramisole hydrochloride	90	Levamisole hydrochloride	Phosphorylation	240.76
718	L-655,708	90	Ethyl (S)-11,12,13,13a-Tetrahydro-7-methoxy-9-oxo-9H-imidazo[1,5-a]pyrrolo[2,1-c][1,4]benzodiazepine-1-carboxylate	Benzodiazepine	341.37
719	LY-294,002 hydrochloride	53	2-(4-Morpholinyl)-8-phenyl-4H-1-benzopyran-4-one hydrochloride	Phosphorylation	343.81
720	Loxapine succinate	3		Dopamine	445.91
721	LY-53,857 maleate	73	6-Methyl-1-(1-methylethyl)- ergoline-8beta-carboxylic acid 2-hydroxy-1-methylpropyl ester maleate	Serotonin	500.60
722	Lorglumide sodium	122	CR 1409	Cholecystokinin	481.40
723	LY-278,584 maleate	92	1-Methyl-N-(8-methyl-8-azabicyclo[3.2.1]-oct-3-yl)-1H-indazole-3-carboxamide maleate	Serotonin	414.47
724	R(+)-Lisuride hydrogen maleate	31	R(+)-N'-[(8alpha)-9,10-Didehydro-6-methylergolin-8-yl]-N,N,-diethylurea hydrogen maleate	Dopamine	454.53
725	L-703,606 oxalate	98	cis-2-(Diphenylmethyl)-N-[(2-iodophenyl)methyl]-1-azabicyclo[2.2.2]octan-3-amine oxalate	Tachykinin	598.49
726	Levallorphan tartrate	93	17-(2-Propenyl)morphinan-3-ol tartrate	Opioid	433.51
727	S-(-)-Lisuride	69	S(-)-N'-[(8a)-9,10-Didehydro-6-methylergolin-8-yl]-N,N-diethyl-urea	Dopamine	338.46
728	L-745,870 hydrochloride	111	3-[[4-(4-Chlorophenyl)piperazin-1-yl]methyl]-1H-pyrrolo[2,3-b]pyridine hydrochloride	Dopamine	363.29
729	L-750,667 trihydrochloride	107	(±)-3-[4-Iodophenyl)-1-piperazyl] methylpyrrolo [2,3-b] pyrimidine	Dopamine	527.67
730	Linopirdine	106	DuP 996	Cholinergic	391.48
731	L-741,626	104	(±)-3-[4-(4-Chlorophenyl)-4-hydroxypiperidinyl]methylindole	Dopamine	340.86
732	L-733,060 hydrochloride	94	(2S,3S) 3-([3,5-Bis(trifluoromethyl)phenyl]methoxy)-2-phenylpiperidine hydrochloride	Tachykinin	439.83
733	Metoclopramide hydrochloride	101		Dopamine	336.26
734	R(-)-Me5	93	1-(2,6-Dimethylphenoxy)-3-methyl-2-butanamine hydroiodide	Na+ Channel	335.23
735	Dihydrocapsaicin	102	8-Methyl-N-vanillylnonanamide	Vanilloid	307.44
736	(-)-Naproxen sodium	105	(S)-6-Methoxy-alpha-methyl-2-naphthaleneacetic acid sodium	Prostaglandin	252.25
737	4-Methylpyrazole hydrochloride	111	Fomepizole	Biochemistry	118.57
738	Nocodazole	89	R 17934	Cytoskeleton and ECM	301.33
739	N-omega-Methyl-5-hydroxytryptamine oxalate salt	115	N-omega-Methylserotonin	Serotonin	280.28
740	Moxonidine hydrochloride	94	BDF-5895	Adrenoceptor	278.14
741	MRS 1845	101	N-Propargylnitrendipene	Ca2+ Channel	398.42
742	N-Methyl-1-deoxynojirimycin	101	1,5-Dideoxy-1,5-imino-1-methyl-D-sorbitol	Biochemistry	177.20
743	MRS 1523	101	3-propyl-6-ethyl-5-[(ethylthio)carbonyl]-2-phenyl-4-propyl-3-pyridine carboxylate	Adenosine	399.56
744	Melphalan	102	L-Phenylalanine mustard	DNA Metabolism	305.21
745	5-Methoxy DMT oxalate	104	5-Methoxy-N,N-dimethyltryptamine hydrogen oxalate	Serotonin	308.34
746	Metaproterenol hemisulfate	111		Adrenoceptor	520.60
747	Mianserin hydrochloride	5	1,2,3,4,10,14b-Hexahydro-2-methyldibenzo[c,f]pyryzino[1,2-a]azepine hydrochloride	Serotonin	300.83
748	Mevastatin	106	Compactin	Antibiotic	390.52
749	8-Methoxymethyl-3-isobutyl-1-methylxanthine	107	8-Methoxymethyl-IBMX	Cyclic Nucleotides	266.30
750	MK-886	65	3-[3-tert-Butylthio-1-(4-chlorobenzyl)-5-isopropyl-1H-indol-2-yl]-2,2-dimethylpropionic acid, sodium salt	Leukotriene	494.08
751	Mexiletene hydrochloride	105	1-(2,6-Xylyloxy)-2-aminopropane	Na+ Channel	215.73
752	Methylergonovine maleate	107	Methergine maleate	Dopamine	455.52
753	Molsidomine	98	SIN-10	Nitric Oxide	242.24

754	3-Methyl-6-(3-[trifluoromethyl]phenyl)-1,2,4-triazolo[4,3-b]pyridazine	87	CL 218,872	Benzodiazepine	278.24
755	Mizoribine	94	N'-(beta-D-Ribofuranosyl)-5-hydroxyimidazole-4-carboxamide	DNA Metabolism	259.22
756	S-Methylisothiourea hemisulfate	99	Carbamimidothioic acid methyl ester hemisulfate	Nitric Oxide	278.37
757	MG 624	89	N,N,N-Triethyl-2-(4-trans-stilbenoxy)ethylammonium iodide	Cholinergic	437.37
758	N-Methyl-D-aspartic acid	97	NMDA	Glutamate	147.13
759	alpha-Methyl-DL-tyrosine methyl ester hydrochloride	101	AMPT	Neurotransmission	245.71
760	MJ33	77	1-Hexadecyl-3-(trifluoroethyl)- sn-glycero-2-phosphomethanol lithium	Lipid	514.49
761	Metergoline	33	[[(8beta)-1,6-Dimethylergolin-8-yl]-methyl]carbamic acid phenylmethyl ester	Serotonin	403.53
762	(-)-cis-(1S,2R)-U-50488 tartrate	98	(-)-(1S,2R)-cis-3,4-Dichloro-N-methyl-N-[2-(1-pyrrolidinyl)cyclohexyl]benzeneacetamide tartrate	Neurotransmission	519.43
763	Clorgyline hydrochloride	96	N-Methyl-N-propargyl-3-(2,4-dichlorophenoxy)-propylamine hydrochloride	Neurotransmission	308.64
764	MRS 2179	105	2'-Deoxy-N6-methyl adenosine 3',5'-diphosphate diammonium salt	P2 Receptor	459.30
765	Meloxicam sodium	115	4-Hydroxy-2-methyl-N-(5-methyl-2-thiazolyl)-2H-1,2-benzothiazine-3-carboxamide 1,1-dioxide	Prostaglandin	373.39
766	Morin	117	2',3,4',5,7-Pentahydroxyflavone	Cell Stress	302.24
767	Minoxidil	110		K+ Channel	209.25
768	3-Methoxy-4-hydroxyphenethylamine hydrochloride	116	3-Methoxytyramine hydrochloride	Dopamine	203.67
769	Meclofenamic acid sodium	107	2-([2,6-Dichloro-3-methylphenyl]amino)benzoic acid sodium	Prostaglandin	318.14
770	Milrinone	104	1,6-Dihydro-2-methyl-6-oxo-[3,4'-bipyridine]-5-carbonitrile	Cyclic Nucleotides	211.23
771	(±)-alpha-Methyl-4-carboxyphenylglycine	108	(±)-MCPG	Glutamate	209.20
772	1-Methylhistamine dihydrochloride	94		Histamine	198.10
773	Moxisylyte hydrochloride	33	4-Dimethylaminoethoxy-5-isopropyl-2-methylphenyl acetate hydrochloride	Adrenoceptor	315.84
774	S-Methyl-L-thiocitrulline acetate	97	N5-[Imino(methylthio)methyl]-L-ornithine acetate	Nitric Oxide	265.33
775	Melatonin	104	N-[2-(5-Methoxyindol-3-yl)ethyl]acetamide	Melatonin	232.28
776	L-Methionine sulfoximine	103	L-S-(3-Amino-3-carboxypropyl)-S-methylsulfoximine	Glutamate	180.23
777	(±)-Metoprolol (+)-tartrate	98	1-(Isopropylamino)-3-(p-[beta-methoxyethyl]phenoxy)-2-propanol tartrate	Adrenoceptor	684.83
778	6-Methyl-2-(phenylethynyl)pyridine hydrochloride	74	MPEP hydrochloride	Glutamate	229.71
779	Mibefradil dihydrochloride	93	Ro 40-5967; (1S,2S)-2-[2[[3-(2-benzimidazolylpropyl]methylamino]ethyl]-6-fluoro-1,2,3,4-tetrahydro-1-isopropyl-2-naphthyl methoxyacetate dihydrochloride	Ca2+ Channel	568.56
780	N6-Methyladenosine	80	6-Methylaminopurine-9-ribofuranoside	Adenosine	281.27
781	(S)-MAP4 hydrochloride	100	(S)-2-Amino-2-methyl-4-phosphonobutanoate hydrochloride	Glutamate	232.60
782	(±)-Methoxyverapamil hydrochloride	88	D600; Gallopamil	Ca2+ Channel	521.10
783	Metrazoline oxalate	91	1H-Imidazole, 4,5-dihydro-2-[(1E)-2-(2-methylphenyl)ethenyl,-ethandioate	Imidazoline	276.29
784	GW9662	109	2-Chloro-5-nitro-N-phenyl-benzamide	Transcription	276.68
785	MRS 1754	98	8-[4-[((4-Cyanophenyl)carbamoylmethyl)oxy]phenyl]-1,3-di(n-propyl)xanthine	Adenosine	486.53
786	2-methoxyestradiol	99	2-Hydroxyestradiol 2-methyl ether	Hormone	302.42
787	Cysteamine hydrochloride	94	Mercaptamine; MEA hydrochloride	Somatostatin	113.61
788	alpha,beta-Methylene adenosine 5'-triphosphate dilithium	97	alpha,beta-Methylene ATP, AMP-CPP dilithium	P2 Receptor	517.08
789	Methoxamine hydrochloride	92		Adrenoceptor	247.72
790	Mitoxantrone	18	1,4-Dihydroxy-5,8-bis([2-([2-hydroxyethyl]amino)ethyl]amino)-9,10-anthracenedione	DNA Metabolism	517.41
791	O-Methylserotonin hydrochloride	118	Mexamine hydrochloride	Serotonin	226.71
792	Se-(methyl)selenocysteine hydrochloride	129	Se-MSC	Cell Cycle	218.54
793	MDL 28170	96	Z-Val-Phe-CHO	Cell Cycle	382.46
794	Myricetin	63	Cannabiscetin	Phosphorylation	318.24
795	NG-Monomethyl-L-arginine acetate	94	L-NMMA	Nitric Oxide	248.28
796	MK-912	73	L-657,743 hydrochloride	Adrenoceptor	375.90
797	(±)-3-(3,4-dihydroxyphenyl)-2-methyl-DL-alanine	99	DL-alpha-Methyl-DOPA	Neurotransmission	211.22
798	MRS 2159	91		P2 Receptor	461.21
799	GR 127935 hydrochloride	90	N-[4-Methoxy-3-(4-methyl-1-piperazinyl)phenyl]-2'-methyl-4'-(5-methyl-1,2,4-oxadiazol-3-yl)-1,1'-biphenyl-4-carboxamide hydrochloride	Serotonin	534.06
800	2,6-Difluoro-4-[2-(phenylsulfonylamino)ethylthio]phenoxyacetamide	89	PEPA	Glutamate	402.44
801	Mifepristone	94	RU-486	Hormone	429.61
802	L-alpha-Methyl-p-tyrosine	100	(S)-alpha-Methyltyrosine	Neurotransmission	195.22
803	Monastrol	103	4-(3-Hydroxyphenyl)-6-methyl-2-thioxo-1,2,3,4-tetrahydro-4H-pyrimidin-5-carboxylic acid ethyl ester	Cell Cycle	292.36
804	1-Methylimidazole	100	Methimazole	Prostaglandin	82.11
805	Mecamylamine hydrochloride	114		Cholinergic	203.76
806	Methapyrilene hydrochloride	88	N,N-Dimethyl-N'-(2-pyridinyl)-N'-(2-thienylmethyl)-1,2-ethanediamine hydrochloride	Histamine	297.85
807	Memantine hydrochloride	91	3,5-Dimethyl-1-adamantanamine hydrochloride	Glutamate	215.77
808	Me-3,4-dephostatin	101	3,4-Dihydroxy-N-methyl-N-nitrosaline	Phosphorylation	168.15
809	Minocycline hydrochloride	94		Cell Cycle	492.96
810	Maprotiline hydrochloride	18	9-(gamma-Methylaminopropyl)-9,10-dihydro-9,10-ethanoanthracene hydrochloride	Adrenoceptor	313.87
811	H-8 dihydrochloride	43	N-[2-(Methylamino)ethyl]-5-isoquinolinesulfonamide dihydrochloride	Phosphorylation	338.26
812	Proglumide	97	4-Benzoylamino-5-dipropylamino-5-oxopentanoic acid	Cholecystokinin	334.42
813	R-(-)-Deprenyl hydrochloride	93	Selegiline	Neurotransmission	223.75
814	(±)-Muscarine chloride	104	Tetrahydro-4-hydroxy-N,N,N,5-tetramethyl-2-furanmethanammonium chloride	Cholinergic	209.72
815	Methoctramine tetrahydrochloride	66	N,N'-bis[6-[[(2-Methoxyphenyl)methyl]amino]hexyl]1,8-octane diamine tetrahydrochloride	Cholinergic	728.77
816	(+)-MK-801 hydrogen maleate	107	Dizocilipine maleate	Glutamate	337.38
817	(-)-MK-801 hydrogen maleate	98	(5S,10R)-(–)-5-Methyl-10,11-dihydro-5H-dibenzo[a,d]cyclohepten-5,10-imine	Glutamate	337.38
818	2-Methyl-5-hydroxytryptamine maleate	102	2-Methylserotonin maleate	Serotonin	306.32
819	alpha-Methyl-5-hydroxytryptamine maleate	92	alpha-Methylserotonin maleate	Serotonin	306.32
820	Metolazone	90		Ion Pump	365.84
821	Metaphit methanesulfonate	93	1-(1-[3-Isothiocyanato]phenyl)cyclohexylpiperidine methansulfonate	Opioid	396.57
822	L-alpha-Methyl DOPA	98	MK-351; Methyldopa	Biochemistry	211.22

823	Methysergide maleate	82		Serotonin	469.54
824	Methylcarbamylcholine chloride	103	Methylcarbachol chloride	Cholinergic	196.68
825	Methiothepin mesylate	1	1-[10,11-Dihydro--8-(methylthio)dibenzo[b,f]thiepin-10-yl]-4-methylpiperazine mesylate	Serotonin	452.66
826	2-Methylthioadenosine diphosphate trisodium	92	2-(Methylthio)-adenosine 5'-trihydrogen diphosphate trisodium	P2 Receptor	539.24
827	Mesulergine hydrochloride	88	CU 32-085 hydrochloride	Dopamine	397.97
828	MDL 26,630 trihydrochloride	93	1,5-(Diethylamino)piperidine trihydrochloride	Glutamate	280.67
829	ZM 39923 hydrochloride	83	3-(N-Benzyl-N-isopropyl)amino-1-(naphthalen-2-yl)propan-1-one hydrochloride	Phosphorylation	367.92
830	3-Morpholinosydnonimine hydrochloride	90	Linsidomine hydrochloride	Nitric Oxide	206.63
831	3-Methoxy-morphanin hydrochloride	94	nor-Dextromethorphan hydrochloride	Glutamate	293.84
832	p-MPPI hydrochloride	73	4-Iodo-N-[2-4-(methoxyphenyl)-1-piperazinyl]ethyl]-N-2-pyridinyl-benzamide hydrochloride	Serotonin	578.88
833	MDL 105,519	83	(Z)-2-Carboxy-4,6-dichloroindole-3-(2'-phenyl-2'-carboxy)-ene	Glutamate	376.20
834	Metrifudil	96	N-[(2-Methylphenyl)methyl]-adenosine	Adenosine	371.40
835	p-MPPF dihydrochloride	81	4-Fluoro-N-(2-[4-(2-methoxyphenyl)1-piperazinyl]ethyl)-N-(2-pyridinyl)benzamide dihydrochloride	Serotonin	507.44
836	(-)-3-Methoxynaltrexone hydrochloride	75		Opioid	391.90
837	Niflumic acid	90	2-(3-[Trifluoromethyl]anilino)nicotinic acid	Prostaglandin	282.22
838	Nimesulide	88	N-(4-Nitro-2-phenoxyphenyl)methanesulfonamide	Prostaglandin	308.31
839	Nialamide	87	4-Pyridinecarboxylic acid 2-[3-oxo-3-[(phenylmethyl)amino]propyl]hydrazide	Neurotransmission	298.35
840	Nomifensine maleate	42	1,2,3,4-Tetrahydro-2-methyl-4-phenyl-8-isoquinolinamine maleate	Dopamine	354.41
841	nor-Binaltorphimine dihydrochloride	98	nor-BNI dihydrochloride	Opioid	734.73
842	Neostigmine bromide	100		Cholinergic	303.20
843	CR 2249	92	Nebostinel	Glutamate	256.35
844	S-(4-Nitrobenzyl)-6-thioinosine	93	NBTI	Adenosine	419.42
845	Naltrexone hydrochloride	101	17-(Cyclopropylmethyl)-4,5-epoxy-3,14-dihydroxymorphinan-6-one hydrochloride	Opioid	377.87
846	S-Nitroso-N-acetylpenicillamine	88	SNAP	Nitric Oxide	220.25
847	Niclosamide	5	2',5'-Dichloro-4'-nitrosalicylanilide	Antibiotic	327.13
848	NAN-190 hydrobromide	42	1-(2-Methoxyphenyl)-4-[4-(2-phthalimido)butyl]piperazine hydrobromide	Serotonin	474.40
849	NCS-356	99	4-(4-chlorophenyl)-4-hydroxy-2-butanoic acid	GABA	234.62
850	S-Nitrosoglutathione	100	GSNO	Nitric Oxide	336.33
851	NCS-382	97	1-Ethyl-1,4-dihydro-7-methyl-4-oxo-1,8-naphthyridine-3-carboxylic acid	GABA	242.25
852	Nalidixic acid sodium	99	1-Ethyl-1,4-dihydro-7-methyl-4-oxo-1,8-naphthyridine-3-carboxylic acid sodium	Antibiotic	254.22
853	Nalbuphine hydrochloride	96		Opioid	393.91
854	5-Nitro-2-(3-phenylpropylamino)benzoic acid	99	NPPB	Cl- Channel	300.32
855	NF449 octasodium salt	112	4,4',4'',4'''-[Carbonyl-bis[imino-5,1,3-benzenetriyl bis-(carbonyl-imino)]]tetrakis(benzene-1,3-disulfonic acid)	G protein	1505.10
856	Nordihydroguaiaretic acid from Larrea divaricata (creosote bush)	64	NDGA	Leukotriene	302.37
857	(-)-Nicotine hydrogen tartrate salt	92	(-)-1-Methyl-2-(3-pyridyl)pyrrolidine	Cholinergic	462.41
858	NG-Nitro-L-arginine	95	L-NOARG; L-NNA	Nitric Oxide	219.20
859	Naphazoline hydrochloride	90	2-(1-Naphthylmethyl)imidazoline nitrate	Adrenoceptor	246.74
860	3-Nitropropionic acid	91		Cell Stress	119.08
861	NG-Nitro-L-arginine methyl ester hydrochloride	96	L-NAME hydrochloride	Nitric Oxide	269.69
862	(±)-Normetanephrine hydrochloride	81	alpha-(Aminomethyl)-4-hydroxy-3-methoxy-benzyl alcohol hydrochloride	Adrenoceptor	219.67
863	Nortriptyline hydrochloride	4		Adrenoceptor	299.85
864	NADPH tetrasodium	92	Reduced nicotinamide adenosine dinucleotide phosphate tetrasodium	Nitric Oxide	833.36
865	Nicardipine hydrochloride	76	YC-93 hydrochloride	Ca2+ Channel	516.00
866	Nifedipine	78		Ca2+ Channel	346.34
867	Naloxone hydrochloride	75		Opioid	363.84
868	7-Nitroindazole	78		Nitric Oxide	163.14
869	NS 521 oxalate	75	1-(1-Butyl)-4-(2-oxo-1-benzimidazolinyl)piperidine oxalate	Glutamate	363.42
870	2-(alpha-Naphthoyl)ethyltrimethylammonium iodide	74	alpha-NETA	Cholinergic	369.25
871	6-Nitroso-1,2-benzopyrone	93		Transcription	175.15
872	Nilutamide	93	Anandron	Hormone	317.23
873	NF 023	94	8,8'-[carbonylbis(imino-3,1-phenylene carbonylimino)]bis(1,3,5-naphthalene-trisulfonic acid) hexasodium salt	P2 Receptor	1162.89
874	Nimustine hydrochloride	88	ACNU	DNA	272.70
875	Norcantharidin	76		Phosphorylation	168.15
876	Noscapine hydrchloride	78	Narcotine	Opioid	449.89
877	(+)-Nicotine (+)-di-p-toluoyl tartrate	80	R(+)-3-(1-Methyl2-pyrrolidinyl)pyridinium (+)di-p-toluoyl tartrate	Cholinergic	548.60
878	Naltrindole hydrochloride	81	NTI hydrochloride	Opioid	450.97
879	N-(p-Isothiocyanatophenethyl)spiperone hydrochloride	83	NIPS hydrochloride	Dopamine	593.17
880	NO-711 hydrochloride	81	1-(2-(((Diphenylmethylene)imino)oxy)ethyl)-1,2,5,6-tetrahydro-3-pyridine-carboxylic acid hydrochloride	GABA	386.88
881	Nitrendipine	103	1,4-Dihydro-2,6-dimethyl-4-(3-nitrophenyl)-3,5-pyridinecarboxylic acid ethyl methyl ester	Ca2+ Channel	360.37
882	Nimodipine	102	1,4-Dihydro-2,6-dimethyl-4-(3-nitrophenyl)- 3,5-pyridinecarboxylic acid 2-methoxyethyl 1-methylethyl ester	Ca2+ Channel	418.45
883	Nisoxetine hydrochloride	84	LY-94,939	Adrenoceptor	307.82
884	Nylidrin hydrochloride	86		Adrenoceptor	335.88
885	N6-Cyclopentyl-9-methyladenine	105	N-0840	Adenosine	217.28
886	Naltriben methanesulfonate	102	NTB	Opioid	511.60
887	Naftopidil dihydrochloride	79	KT-611 dihydrochloride	Adrenoceptor	465.42
888	BW 245C	120	(R*,S*)-(+/-)-3-(3-cyclohexyl-3-hydroxypropyl)-2,5-dioxo-4-imidazolineheptanoic acid	Prostanoids	368.48
889	Naloxone benzoylhydrazone	101	[(5alpha)-4,5-Epoxy- 3,14-dihydroxy-17-(2-propenyl)morphinan-6-ylidene]hydrazide benzoic acid	Opioid	445.52
890	NS-1619	86	1,3-Dihydro-1-[2-hydroxy-5-(trifluoromethyl)phenyl]-5-(trifluoromethyl)-2H-benzimidazol-2-one	K+ Channel	362.23
891	Naloxonazine dihydrochloride	86	Bis(5-alpha-4,5-epoxy-3,14,-dihydroxy-17-[2-propenyl]morphinan-6-ylidene)hydrazone	Opioid	723.70

892	NBQX disodium	95	FG9202 disodium	Glutamate	380.25
893	NS 2028	98	4H-8-Bromo-1,2,4-oxadiazolo(3,4-d)benz(b)(1,4)oxazin-1-one	Cyclic Nucleotides	269.06
894	(±)-Octopamine hydrochloride	104	(±)-alpha-(Aminomethyl)-4-hydroxybenzyl alcohol hydrochloride	Adrenoceptor	189.64
895	N-Oleoylethanolamine	105	N-(Hydroxyethyl)oleamide	Sphingolipid	325.54
896	Oxolinic acid	108	W-4565; 5,8-Dihydro-5-ethyl-8-oxo-1,3-dioxolo[4,5-g]quinoline-7-carboxylic acid	Antibiotic	261.24
897	Olomoucine	47	2-[[9-Methyl-6-[(phenylmethyl)amino]-9H-purin-2-yl]amino]-ethanol	Phosphorylation	298.35
898	Oleic Acid	95	Elainic acid	Phosphorylation	282.47
899	Oxymetazoline hydrochloride	84	3-[(4,5-Dihydro- 1H-imidazol-2-yl)methyl]-6-(1,1-dimethylethyl)-2,4-dimethylphenol hydrochloride	Adrenoceptor	296.84
900	Sodium Oxamate	91	Oxalic acid monoamide sodium salt	Biochemistry	111.03
901	Oxybutynin Chloride	83	alpha-Phenylcyclohexaneglycolic acid 4-(diethylamino)-2-butynyl ester hydrochloride	Cholinergic	393.96
902	Oxiracetam	101	4-Hydroxy-2-oxopyrrolidine-N-acetamide	Nootropic	158.16
903	Ouabain	50	Acocantherine	Ion Pump	584.67
904	ODQ	98	1H-[1,2,4]Oxadiazolo[4,3-a]quinoxalin-1-one	Cyclic Nucleotides	187.16
905	Orphenadrine hydrochloride	29	beta-Dimethylaminoethyl 2-methylbenzhydryl ether hydrochloride	Cholinergic	305.85
906	Oligomycin A	82		Antibiotic	791.08
907	Ofloxacin	92	Ofloxacine; DL-8280; HOE-280	Antibiotic	361.38
908	Oxotremorine sesquifumarate salt	88	1-(4-[1-Pyrrolidinyl]-2-butynyl)-2-pyrrolidinone	Cholinergic	760.80
909	Oxatomide	72	1-[3-[4-(Diphenylmethyl)-1-piperazinyl]propyl]-1,3-dihydro-2H-benzimidazol-2-one	Immune System	426.57
910	SB 216763	80	3-(2,4-Dichlorophenyl)-4-(1-methyl-1H-indol-3-yl)-1H-pyrrole-2,5-dione	Phosphorylation	371.23
911	Oxaprozin	106	Daypro	Prostaglandin	293.33
912	Oxotremorine methiodide	99	N,N,N-Trimethyl-4-(2-oxo-1-pyrrolidinyl)-2-butyn-1-ammonium iodide	Cholinergic	322.19
913	(±)-Octoclothepin maleate	3	1-(8-Chloro-10,11-dihydrobenzo[b,f]thiepin-10-yl)-4-methyl-piperazine maleate	Dopamine	460.98
914	Progesterone	77	4-Pregnene-3,20-dione	Hormone	314.47
915	Palmitoylethanolamide	89	Palmidrol	Cannabinoid	299.50
916	Piceatannol	2	(E)-4-[2-(3,5-Dihydroxyphenyl)ethenyl]1,2-benzenediol	Phosphorylation	244.25
917	Pentamidine isethionate	87	4'4-[1,5-Pentanediylbis(oxy)]bis-benzenecarboximidamide isethionate	Glutamate	592.69
918	cis-(±)-8-OH-PBZI hydrobromide	88	cis-(±)-1,2,3a,4,5,9b-hexhydro-1H-benz[e]indole hydrobromide	Dopamine	312.25
919	Parthenolide	74		Serotonin	248.32
920	Pindolol	96		Adrenoceptor	248.33
921	O-Phospho-L-serine	102	L-Phosphoserine	Glutamate	185.07
922	(±)-Propranolol hydrochloride	102	(±)-1-(Isopropylamino)-3-(1-naphthyloxy)-2-propanol hydrochloride	Adrenoceptor	295.81
923	SKF-525A hydrochloride	88	Proadifen hydrochloride	Multi-Drug Resistance	389.97
924	Picrotoxin	98		GABA	602.60
925	4-Phenyl-3-furoxancarbonitrile	93	Furoxan	Nitric Oxide	187.16
926	Pentoxifylline	119	3,7-Dihydro-3,7-dimethyl-1-(5-oxohexyl)-1H-purine-2,6-dione	Cyclic Nucleotides	278.31
927	Pimozide	61		Dopamine	461.56
928	L-Glutamic acid, N-phthaloyl-	106		Glutamate	277.24
929	Pancuronium bromide	96	1,1'-([2beta,3alpha,5alpha,16beta,17beta]-3,17-Bis[acetyloxy]androstane-2,16-diyl)bis(1-methylpiperidinium) dibromide	Cholinergic	732.69
930	3-alpha,21-Dihydroxy-5-alpha-pregnan-20-one	96	5-alpha-THDOC	GABA	334.50
931	Pirfenidone	102	5-Methyl-1-phenyl-2-(1H)-pyridone	Immune System	185.23
932	1,3-Dimethyl-8-phenylxanthine	107	8-Phenyltheophylline	Adenosine	256.27
933	PRE-084	109		Opioid	353.89
934	PPNDS tetrasodium	106	Pyridoxal-5'-phosphate-6-(2'-naphthylazo-6'-nitro-4',8'-disulfonate) tetrasodium	P2 Receptor	694.37
935	PD 404,182	101	6H-6-Imino-(2,3,4,5-tetrahydropyrimido)[1,2-c]-[1,3]benzothiazine	Biochemistry	217.29
936	Papaverine hydrochloride	92		Cyclic Nucleotides	375.86
937	Pentolinium di[L(+)-tartrate]	94	1,1'-Pentamethylenebis(1-methylpyrrolidinium hydrogen tartrate)	Cholinergic	538.60
938	1-Phenyl-3-(2-thiazolyl)-2-thiourea	84		Dopamine	235.33
939	Thiolactomycin	96	[R-(E)]-4-hydroxy-3,5-dimethyl-5-(2-methyl-1,3-butadienyl)-2(5H)-thiophenone	Antibiotic	210.30
940	Cisplatin	88	cis-Diammineplatinum(II) dichloride	DNA	300.06
941	Podophyllotoxin	84		Cytoskeleton and ECM	414.42
942	Purvalanol A	7	NG-60	Phosphorylation	388.90
943	Palmitoyl-DL-Carnitine chloride	59		Phosphorylation	436.08
944	R(-)-N6-(2-Phenylisopropyl)adenosine	112	R(-)-PIA	Adenosine	385.43
945	Valproic acid sodium	85	2-Propylpentanoic acid sodium	Anticonvulsant	166.20
946	Promethazine hydrochloride	5		Histamine	320.89
947	Praziquantel	81	2-(Cyclohexylcarbonyl)-1,2,3,6,7-11b-hexahydro-4H-pyrazinoe(2,1a)isoquinolin-4-one	Antibiotic	312.42
948	Propafenone hydrochloride	74	1-(2-[2-Hydroxy-3-(propylamino)propoxy]phenyl)-3-phenyl-1propanone	K+ Channel	377.92
949	5alpha-Pregnan-3alpha-ol-11,20-dione	89	Alfaxalone	GABA	332.49
950	Pempidine tartrate	100	Pirilene	Cholinergic	305.37
951	Piracetam	103	2-Oxo-1-pyrrolidineacetamide	Glutamate	142.16
952	Phosphomycin disodium	104	Fosfomycin; Phosphonomycin; MK-955	Antibiotic	182.02
953	Pyrilamine maleate	59	Mepyramine maleate	Histamine	401.47
954	Piroxicam	83	4-Hydroxy-2-methyl-3-(pyrid-2-yl-carbamoyl)-2H-1,2-benzothiazine 1,1-dioxide	Prostaglandin	331.35
955	3-n-Propylxanthine	93	Enprofylline	Adenosine	194.19
956	Phenylephrine hydrochloride	93		Adrenoceptor	203.67
957	Perphenazine	5		Dopamine	403.98
958	Pentylenetetrazole	94	Metrazole	Neurotransmission	138.17
959	(+)-Pilocarpine hydrochloride	104		Cholinergic	244.72
960	Pilocarpine nitrate	96		Cholinergic	271.28

961	Promazine hydrochloride	12	10-(3-[Dimethylamino]propyl)phenothiazine hydrochloride	Dopamine	320.89
962	Phenelzine sulfate	93		Neurotransmission	234.28
963	Pheniramine maleate	91		Histamine	356.43
964	Phosphonoacetic acid	84		DNA	140.03
965	(-)-Perillic acid	87	4-Isopropenyl-1-cyclohexene-1-carboxylic acid	G protein	166.22
966	Pyrazinecarboxamide	79	Pyrazinoic acid amide; pyrazinamide; PZA	Antibiotic	123.12
967	Primidone	94		Anticonvulsant	218.26
968	(±)-threo-1-Phenyl-2-decanoylamino-3-morpholino-1-propanol hydrochloride	85	PDMP hydrochloride	Sphingolipid	427.03
969	Pirenzepine dihydrochloride	114	5,11-Dihydro-11- [(4-methyl-1-piperazinyl)acetyl]-6H-pyrido[2,3-b][1,4]benzodiazepin-6-one dihydrochloride	Cholinergic	424.33
970	Putrescine dihydrochloride	88	Putrescine dihydrochloride	Glutamate	161.08
971	Phentolamine mesylate	42	Regitin mesylate	Adrenoceptor	377.47
972	Propionylpromazine hydrochloride	15		Dopamine	376.95
973	Prazosin hydrochloride	81		Adrenoceptor	419.87
974	Phloretin	99	3-(4-Hydroxyphenyl)-1-(2,4,6-trihydroxyphenyl)-1-propanone	Ca2+ Channel	274.28
975	Pargyline hydrochloride	95	N-Methyl-N-2-propynylbenzylamine hydrochloride	Neurotransmission	195.69
976	Phorbol 12-myristate 13-acetate	12	PMA	Phosphorylation	616.84
977	1,3-PBIT dihydrobromide	117	Phenylene-1,3-bis(ethane-2-isothiourea) dihydrobromide	Nitric Oxide	444.26
978	Protoporphyrin IX disodium	86	Kammerer's porphyrin	Cyclic Nucleotides	562.67
979	1,4-PBIT dihydrobromide	120	1,4-Phenylene-bis(1,2-ethanediyl)bis-isothiourea	Nitric Oxide	444.26
980	Phenylbutazone	96		Prostaglandin	308.38
981	Picotamide	84	4-Methoxy-N,N'-bis(3-pyridinylmethyl)-1,3-benzenedicarboxamide	Thromboxane	376.42
982	Tranylcypromine hydrochloride	73	(±)-trans-2-Phenylcyclopropylamine hydrochloride	Neurotransmission	169.66
983	(S)-Propranolol hydrochloride	90	(S)-1-Isopropylamino-3-(1-naphthyloxy)-2-propanol hydrochloride	Adrenoceptor	295.81
984	Ammonium pyrrolidinedithiocarbamate	85	APDC	Nitric Oxide	164.29
985	(±)-cis-Piperidine-2,3-dicarboxylic acid	108		Glutamate	173.17
986	Protriptyline hydrochloride	18		Adrenoceptor	299.85
987	Pergolide methanesulfonate	53	8-[(Methylthio)methyl]-6-propylergoline methanesulfonate	Dopamine	410.60
988	6(5H)-Phenanthridinone	90		Transcription	195.22
989	5alpha-Pregnan-3alpha-ol-20-one	73	Allopregnan-3alpha-ol-20-one	GABA	318.50
990	Propantheline bromide	71	(2-Hydroxyethyl)diisopropylmethylammonium bromide xanthene-9-carboxylate bromide	Cholinergic	448.40
991	Piperidine-4-sulphonic acid	105	P4S	GABA	165.21
992	Prochlorperazine dimaleate	17		Dopamine	606.10
993	Piribedil maleate	120	2-(4-[1,3-Benzodioxol-5-ylmethyl]-1-piperazinyl)pyrimidine	Dopamine	414.42
994	Paromomycin sulfate	96		Antibiotic	713.72
995	1,10-Phenanthroline monohydrate	95	o-Phenanthroline monohydrate	Biochemistry	180.21
996	Procainamide hydrochloride	100		Na+ Channel	271.79
997	Prilocaine hydrochloride	95	N-(2-Methylphenyl)-2-(propylamino)propanamide hydrochloride	Na+ Channel	256.78
998	Propentofylline	87	HWA 285	Adenosine	306.37
999	(S)-(-)-propafenone hydrochloride	77	(S)-1-(2-[2-Hydroxy-3-(propylamino)propoxy]phenyl)-3-phenyl-1-propanone hydrochloride	Adrenoceptor	377.92
1000	Pyridostigmine bromide	106		Cholinergic	261.12
1001	Procaine hydrochloride	96	Novocaine hydrochloride	Na+ Channel	272.78
1002	2-Phenylaminoadenosine	106	CV-1808	Adenosine	358.36
1003	R(+)-3PPP hydrochloride	86	R(+)-3-(3-Hydroxyphenyl)-N-propylpiperidine hydrochloride	Dopamine	255.79
1004	S(-)-3PPP hydrochloride	89	Preclamol hydrochloride	Dopamine	255.79
1005	(±)-PPHT hydrochloride	73	(±)-2-(N-Phenylethyl)-N-propyl)amino-5-hydroxytetralin hydrochloride	Dopamine	345.92
1006	3-Phenylpropargylamine hydrochloride	91		Dopamine	167.64
1007	N6-2-Phenylethyladenosine	104		Adenosine	371.40
1008	N6-Phenyladenosine	99		Adenosine	343.34
1009	Phaclofen	71	3-Amino-2-(4-chlorophenyl)propylphosphonic acid	GABA	249.64
1010	(±)-Pindobind	65	N8-Bromoacetyl-N1-3'-(4-indolyloxy)-2'-hydroxy-propyl-[Z]-1,8-diamino-p-methane	Adrenoceptors	480.45
1011	1-Phenylbiguanide	81	N-Phenyl-imidocarbonimidic diamide	Serotonin	177.21
1012	SKF 94836	72	N-Cyano-N'-methyl-N''-[4-(1,4,5,6-tetrahydro-4-methyl-6-oxo-3-pyridazinyl)phenyl]guanidine	Calcium Signaling	284.32
1013	Pirenperone	10	R-47,465	Serotonin	393.47
1014	IC 261	78	1,3-Dihydro-3-[(2,4,6-trimethoxyphenyl)methylene]-2H-indol-2-one	Phosphorylation	311.34
1015	S(-)-Pindolol	87		Serotonin	248.33
1016	Pinacidil	89	(±)-N-Cyano-N'-4-pyridinyl-N''-(1,2,2-trimethylpropyl)-guanidine	K+ Channel	245.33
1017	Pregnenolone sulfate sodium	107	5-Pregnen-3beta-ol-20-one sulfate sodium	GABA	418.53
1018	PPADS	103	Pyridoxal-phosphate-6-azophenyl-2',4'-disulphonic acid tetrasodium	P2 Receptor	599.31
1019	S(+)-PD 128,907 hydrochloride	89	S(+)-(4aR,10bR)-3,4,4a,10b- Tetrahydro-4-propyl-2H,5H-[1]benzopyrano-[4,3-b]-1,4-oxazin-9-ol hydrochloride	Dopamine	285.77
1020	Phenamil methanesulfonate	46	3,5-Diamino-6-chloro-N-[imino(phenylamino)methyl]-pyrazinecarboxamide methansulfonate	Na+ Channel	401.83
1021	Phenylbenzene-omega-phosphono-alpha-amino acid	95	PMBA	Glycine	335.30
1022	Phthalamoyl-L-glutamic acid trisodium	97	PhGA	Glutamate	361.20
1023	PD 98,059	94	2-(2-Amino-3-methoxyphenyl)-4H-1-benzopyran-4-one	Phosphorylation	267.29
1024	(±)-PD 128,907 hydrochloride	95	PD 125,530	Dopamine	285.77
1025	PD 168,077 maleate	91	N-[[4-(2-Cyanophenyl)-1-piperazinyl]methyl]-3-methyl-benzamide maleate	Dopamine	450.50
1026	SU 6656	65	2,3-Dihydro-N,N-dimethyl-2-oxo-3-[(4,5,6,7-tetrahydro-1H-indol-2-yl)methylene]-1H-indole-5-sulfonamide	Phosphorylation	371.46
1027	Quinolinic acid	83	Pyridine-2,3-dicarboxylic acid	Glutamate	167.12
1028	Quercetin dihydrate	34	3,3',4',5,7-Pentahydroxyflavone dihydrate	Cyclic Nucleotides	302.24
1029	Quinidine sulfate	69		Na+ Channel	746.93

1030	Quipazine dimaleate	71	2-(1-Piperazinyl)quinoline dimaleate	Serotonin	445.43
1031	Quinine sulfate	79		K+ Channel	746.93
1032	(+)-Quisqualic acid	80	L(+)-alpha-Amino-3,5-dioxo-1,2,4-oxadiazolidine-2-propanoic acid	Glutamate	189.13
1033	Quinacrine dihydrochloride	94		Neurotransmission	472.89
1034	Quazinone	108	Ro 13-6438	Cyclic Nucleotides	235.67
1035	(-)-Quinpirole hydrochloride	97	LY-171,555	Dopamine	255.79
1036	Quipazine, N-methyl-, dimaleate	74	2-[1-(4-Methyl)-piperazinyl]quinoline dimaleate	Serotonin	459.46
1037	Quipazine, 6-nitro-, maleate	92	6-Nitro-2-(1-piperzinyl)-quinoline maleate	Serotonin	374.36
1038	Quinelorane dihydrochloride	99	LY-163,502	Dopamine	319.28
1039	(±)-Quinpirole dihydrochloride	98	LY-141,865	Dopamine	292.25
1040	Cortexolone	99	11-Deoxycortisol	Hormone	346.47
1041	Ritodrine hydrochloride	119	N-(p-Hydroxyphenethyl)-4-hydroxynorephedrine hydrochloride	Adrenoceptor	323.82
1042	Raloxifene hydrochloride	103	LY 139481	Hormone	510.06
1043	Retinoic acid	106	Vitamin A acid	Apoptosis	300.44
1044	Ruthenium red	61	Ammoniated ruthenium oxychloride	Ion Pump	786.36
1045	13-cis-retinoic acid	72	Isotretinoin	Transcription	300.44
1046	Rutaecarpine	78	Rutecarpine	K+ Channel	287.32
1047	Ropinirole hydrochloride	103	SKF 101468; 4-[2-(dipropylamino)ethyl]-1,3-dihydro-2H-indol-2-one hydrochloride	Dopamine	296.84
1048	Resveratrol	11	5-[(1E)-2-(4-Hydroxyphenyl)ethenyl]-1,3-benzenediol	Prostaglandin	228.25
1049	REV 5901	103	alpha-Pentyl-3-[2-quinolinylmethoxy]benzyl alcohol	Leukotriene	335.45
1050	Rottlerin	35	Mallotoxin	Phosphorylation	516.55
1051	Ranolazine dihydrochloride	100	N-(2,6-Dimethylphenyl)-4-[2-hydroxy-3-(2-methoxyphenoxy)□propyl]-1-piperazineacetamide dihydrochloride; (±) -4-[2-hydroxy-3-(o-methoxyphenoxy)propy	Lipid	500.47
1052	Rolipram	118	ZK 62711	Cyclic Nucleotides	275.35
1053	Ro 25-6981 hydrochloride	70	(R-[R*,S*])-alpha-(4-Hydroxyphenyl)-beta-methyl-4-(phenylmethyl)-1-peperidinepropanol) hydrochloride	Glutamate	375.94
1054	Phosphoramidon disodium	104	N-(alpha-Rhamnopyranosyloxyhydroxyphosphinyl)-Leu-Trp disodium	Biochemistry	587.48
1055	Roscovitine	28	(R)-2-(1-Ethyl-2-hydroxyethylamino)-6-benzylamino-9-isopropylpurine	Phosphorylation	354.46
1056	Rotenone	41		Cell Stress	394.43
1057	Ro 8-4304	115	(4-[3-(4-[4-fluorophenyl]-3,6-dihydro-2H-pyridin-1-yl)-2-hydroxypropoxy]benzamide	Glutamate	406.89
1058	RX 821002 hydrochloride	121	2-[2-(2-Methoxy-1,4-benzodioanyl)]-imidazoline hydrochloride	Adrenoceptor	270.72
1059	Ribavirin	119	1-beta-D-Ribofuranosyl-1,2,4-triazole-3-carboxamide	Cell Cycle	244.21
1060	Ranitidine hydrochloride	106	Zantac	Histamine	350.87
1061	Ritanserin	18	6-[2-[4-bis(4-Fluorophenyl)methylene]-1-piperidinyl]-□ethyl]-7-methyl-5H-thiazolo[3,2-a]pyrimidin-5-one	Serotonin	477.58
1062	Rauwolscine hydrochloride	87	alpha-Yohimbine hydrochloride	Adrenoceptor	390.91
1063	Ro 16-6491 hydrochloride	107	N-(2-Aminoethyl)-4-chlorobenzamide hydrochloride	Neurotransmission	235.11
1064	Ro 41-1049 hydrochloride	98	N-(2-Aminoethyl)-5-(3-fluorophenyl)-4-thiazolecarboxamide hydrochloride	Neurotransmission	301.77
1065	Ro 41-0960	88	2'-Fluoro-3,4-dihydroxy-5-nitrobenzophenone	Neurotransmission	277.21
1066	Reactive Blue 2	93	Basilen blue E-3G	P2 Receptor	840.11
1067	Riluzole	109	2-Amino-6-(trifluoromethoxy)-benzothiazole	Glutamate	234.20
1068	Risperidone	17	R 62 766	Dopamine	410.50
1069	S(+)-Raclopride L-tartrate	76		Dopamine	497.33
1070	Sobuzoxane	96	4,4'-(1,2-Ethanediyl)bis(1-isobutoxycarbonyloxymethyl-2,6-piperazinedione)	Gene Regulation	514.54
1071	Rilmenidine hemifumarate	114	N-(Dicyclopropylmethyl)-4,5-dihydro-2-oxazolamine;□Oxaminozoline	Imidazoline	180.25
1072	R(-)-Denopamine	107	(-)-alpha-(3,4-dimethoxyphenethylaminomethyl)-4-hydroxybenzylalcohol	Adrenoceptor	317.39
1073	Ro 04-6790 dihydrochloride	135	4-Amino-N-[2,6-bis(methylamino)-4-pyrimidinyl]-benzenesulfonamide dihydrochloride	Serotonin	381.29
1074	(±)-Sotalol hydrochloride	125	N-(4-[1-Hydroxy-2-(isopropylamino)ethyl]phenyl)methanesulfonamide hydrochloride	Adrenoceptor	308.83
1075	SB-366791	105	Vanilloid receptor-1 antagonist	Vanilloid	287.75
1076	Sodium nitroprusside dihydrate	118	Sodium nitroferricyanide	Nitric Oxide	261.92
1077	(±)-Synephrine	88	4-hydroxy-alpha-(methylaminomethyl)benzyl alcohol	Adrenoceptor	167.21
1078	Sulfaphenazole	112	4-Amino-N-(1-phenyl-1H-pyrazol-5-yl)-benzenesulfonamide	Multi-Drug Resistance	314.37
1079	Seglitide	102	MK 678	Somatostatin	808.99
1080	Sulindac sulfone	122	(Z)-5-Fluoro-2-methyl-1-[p-(methylsulfonyl)benzylidene]indene-3-acetic acid	Prostaglandin	372.42
1081	Cortexolone maleate	83	N-(4-[4-(2-methoxyphenyl)-piperazin-1-yl]-butyl-5-(dimethylamino)-napthalene-1-sulfonamide maleate	Dopamine	612.75
1082	SKF 86466	37	6-Chloro-2,3,4,5-tetrahydro-3-methyl-1H-3-benzazepine hydrochloride	Adrenoceptor	232.15
1083	SR 57227A	100	4-amino-1-(6-chloro-2-pyridyl)-piperidine hydrochloride	Serotonin	248.16
1084	(-)-Scopolamine hydrobromide	107	Hyoscine hydrobromide	Cholinergic	384.27
1085	SC-560	92	5-(4-Chlorophenyl)-1-(4-methoxyphenyl)-3-trifluoromethyl pyrazole	Prostaglandin	352.75
1086	Semicarbazide hydrochloride	107	Hydrazine carboxamide hydrochloride	Neurotransmission	111.53
1087	(-)-Scopolamine methyl nitrate	113	Hyoscine methyl nitrate	Cholinergic	380.40
1088	DL-Stearoylcarnitine chloride	101		Phosphorylation	464.13
1089	Spermidine trihydrochloride	104	N-(3-Aminopropyl)-1,4-butanediamine trihydrochloride	Glutamate	254.63
1090	SNC80	94	(+)-4-[(alphaR)-alpha-((2S,5R)-4-Allyl-2,5-dimethyl-1-piperazinyl)-3-methoxybenzyl]-N,N-diethylbenzamide	Opioid	449.64
1091	SKF 83959 hydrobromide	50	6-chloro-7,8-dihydroxy-3-methyl-1-(3-methylphenyl)-2,3,4,5-tetrahydro-1H-3-benzazepine hydrobromide	Dopamine	398.73
1092	Spermine tetrahydrochloride	88	N,N'-bis(3-Aminopropyl)-1,4-butanediamine tetrahydrochloride	Glutamate	348.19
1093	SKF 75670 hydrobromide	39	7,8-dihydroxy-3-methyl-1-phenyl-2,3,4,5-tetrahydro-1H-3-benzazepine hydrobromide	Dopamine	350.26
1094	SC 19220	99	2-acetylhydrazide 10(11H)-carboxylic acid	Prostaglandin	331.76
1095	SKF 89626	89	4-(3,4-dihydroxyphenyl)-4,5,6,7-tetrahydrothieno[2,3-c]pyridine	Dopamine	328.23
1096	SKF 83565 hydrobromide	57	6-chloro-1-(3-chlorophenyl)-7,8-dihydroxy-3-methyl-2,3,4,5-tetrahydro-1H-3-benzazepine hydrobromide	Dopamine	419.15
1097	SB 204070 hydrochloride	122	1-Butyl-4-piperidinylmethyl-8-amino-7-chloro-2,3-dihydro-1,4-benzodioxin-5-carboxylate hydrochloride	Serotonin	419.35
1098	N-Oleoyldopamine	122	OLDA	Neurotransmission	417.64

1099	Spironolactone	84		Hormone	416.58
1100	SCH-202676 hydrobromide	102	N-(2,3-diphenyl-1,2,4-thiadiazol-5-(2H)-ylidene)methanamine hydrobromide	G protein	348.27
1101	D-Serine	100	R(-)-2-Amino-3-hydroxypropionic acid	Glutamate	105.09
1102	Albuterol hemisulfate	106	Salbutamol hemisulfate	Adrenoceptor	576.71
1103	Sanguinarine chloride	75	13-Methyl-[1,3]benzodioxolo[5,6-c]-1,3-dioxolo[4,5-i] phenanthridinium chloride	Ion Pump	367.79
1104	N-Succinyl-L-proline	111		Neurotransmission	215.21
1105	Sphingosine	106	4-Sphingenine	Phosphorylation	299.50
1106	SB 269970 hydrochloride	98	[R]-3-[2-(2-[4-Methyl-piperidin-1-yl]ethyl)pyrrolidine-1-sulfonyl]phenol hydrochloride	Serotonin	388.96
1107	Spiperone hydrochloride	29	R 5147 hydrochloride; Spiroperidol hydrochloride	Dopamine	431.94
1108	SR 2640	91	2-[[3-(2-Quinolinylmethoxy)phenyl]amino]-benzoic acid; QMPB	Leukotriene	370.41
1109	(-)-Sulpiride	87	(-)-5-Aminosulfonyl-N-[(1-ethyl-2-pyrrolidinyl)methyl]-2-methoxybenzamide	Dopamine	341.43
1110	SKF 96365	68	1-(beta-[3-(4-Methoxyphenyl)propoxy]-4-methoxyphenethyl)-1H-imidazole hydrochloride	Ca2+ Channel	402.93
1111	(-)-Scopolamine,n-Butyl-, bromide	103	Butylscopolamine bromide	Cholinergic	440.38
1112	SB 205384	81	4-Amino-7-hydroxy-2-methyl-5,6,7,8-tetrahydrobenzo[b]thieno[2,3-b]pyridine-3-carboxylic acid but-2-ynyl ester	GABA	330.41
1113	(±)-Sulpiride	114	(±)-5-(Aminosulfonyl)-N-[(1-ethyl-2-pyrrolidinyl)methyl]-2-methoxybenzamide	Dopamine	341.43
1114	CV-3988	101	(+/-)-(3-(N-Octadecylcarbamoyloxy)-2-methoxy)propyl-2-thiazolioethyl phosphate	Cytokines & Growth Facto	592.78
1115	Sulindac	107	(Z)-5-Fluoro-2-methyl-1-[[4-(methyl-sulfinyl)phenyl]methylene]-1H-indene-3-acetic acid	Prostaglandin	356.42
1116	Succinylcholine chloride	94		Cholinergic	361.31
1117	Salbutamol	107	Albuterol	Adrenoceptor	239.32
1118	Salmeterol	104	(±) 4-Hydroxy-a1-[[[6-(4-phenylbutoxy)hexyl]amino]m-ethyl]-1,3-benzenedimethanol; GR 33343X	Adrenoceptor	415.58
1119	SU 5416	111	1,3-Dihydro-3-[(3,5-dimethyl-1H-pyrrol-2-yl) methylene]-2H-indol-2-one	Phosphorylation	238.29
1120	(-)-Scopolamine methyl bromide	114	Hyoscine methyl bromide	Cholinergic	398.30
1121	SU 4312	51	3-(4-Dimethylaminobenzylidenyl)-2-indolinone	Phosphorylation	264.33
1122	SR 59230A oxalate	75	3-(2-ethylphenoxy)-1[(1S)-1,2,3,4-tertahydronaphth-1-ylamino]-(2S)-2-propanol oxalate	Adrenoceptor	415.49
1123	BRL 52537 hydrochloride	97	(+/-)-1-(3,4-Dichlorophenyl)acetyl-2-(1-pyrrolidinyl)methylpiperidine hydrochloride	Neurotransmission	391.77
1124	SKF 89976A hydrochloride	95	1-(4,4-Diphenyl-3-butenyl)-3-piperidinecarboxylic acid hydrochloride	GABA	371.91
1125	SIB 1757	46	6-Methyl-2-(phenylazo)-3-pyridinol	Glutamate	213.24
1126	SIB 1893	83	(E)-2-Methyl-6-[2-phenylethenyl]pyridine	Glutamate	195.27
1127	1-(1-Naphthyl)piperazine hydrochloride	79		Serotonin	248.76
1128	Ketanserin tartrate	53	R 41468	Serotonin	545.53
1129	1-(2-Methoxyphenyl)piperazine hydrochloride	64	2-MPP hydrochloride	Serotonin	228.72
1130	PAPP	66	LY-165,163; p-Aminophenethyl-m-trifluoromethylphenyl piperazine	Serotonin	349.40
1131	Spiroxatrine	90	R 5188	Serotonin	379.46
1132	SR-95531	101	2-(3-Carboxypropyl)-3-amino-6-(4-methoxyphenyl)pyridazinium bromide	GABA	368.23
1133	(±)-6-Chloro-PB hydrobromide	99	(±)-SKF-81297 hydrobromide	Dopamine	370.68
1134	SKF 91488 dihydrochloride	89	4-(N,N-Dimethylamino)butylisothiourea dihydrochloride	Histamine	248.22
1135	Suramin hexasodium	112		P2 Receptor	1429.19
1136	SQ 22536	120	9-(Tetrahydro-2-furanyl)-9H-purin-6-amine	Cyclic Nucleotides	205.22
1137	Sepiapterin	110	S(-)-2-Amino-7,8-dihydro-6-(2-hydroxy-1-oxopropyl)-4(1H)-pteridione	Nitric Oxide	237.22
1138	R(-)-SCH-12679 maleate	36	R(-)-1-Phenyl-2,3,4,5-tetrahydro-1H-7,8-dimethoxy-3-benzazepine maleate	Dopamine	413.47
1139	(±)-SKF 38393, N-allyl-, hydrobromide	70	(±)-7,8-Dihydroxy-3-allyl-1-phenyl-2,3,4,5-tetrahydro-1H-3-benzazepine hydrobromide	Dopamine	376.30
1140	SDZ-205,557 hydrochloride	96	4-Amino-5-chloro-2-methoxy-benzoic acid 2-(diethylamino)ethyl ester hydrochloride	Serotonin	337.25
1141	SB 206553 hydrochloride	73	N-3-Pyridinyl- 3,5-dihydro-5-methyl-benzo[1,2-b:4,5-b']dipyrrole-1(2H)-carboxamide hydrochloride	Serotonin	328.80
1142	SB 224289 hydrochloride	95	1'-Methyl-5-([2'-methyl-4'-(5-methyl-1,2,4-oxadiazol-3-yl)biphenyl-4-yl] carbonyl)-2,3,6,7-tetrahydro-spiro(furo[2,3-f]indole-3,4'-piperidine)	Serotonin	557.10
1143	L-Tryptophan	114	S(-)-1-alpha-Aminoindole-3-propionic acid	Serotonin	204.23
1144	Tranilast	73	SB-252218	Leukotriene	327.34
1145	Tiapride hydrochloride	96	N-(2-[Diethylamino]ethyl)-5-(methylsulfonyl)-o-anisamide hydrochloride	Dopamine	364.89
1146	Taurine	101	2-Aminoethanesulfonic acid	Glycine	125.15
1147	Thiothixene hydrochloride	14	SKF-5019 hydrochloride	Dopamine	480.10
1148	Tolbutamide	112		Hormone	270.35
1149	Tetraethylthiuram disulfide	89	Disulfiram	Biochemistry	296.54
1150	TCPOBOP	91	1,4-Bis-[2-(3,5-dichloropyridyloxy)]benzene	Transcription	402.07
1151	Tetraisopropyl pyrophosphoramide	111	iso-OMPA	Biochemistry	342.36
1152	Tetramisole hydrochloride	118	(±)-2,3,5,6-Tetrahydro-6-phenylimidazo[2,1-b]thiazole hydrochloride	Phosphorylation	240.76
1153	Trihexyphenidyl hydrochloride	92		Cholinergic	337.94
1154	Theophylline	98	1,3-Dimethylxanthine	Adenosine	179.18
1155	(E)-4-amino-2-butenoic acid	126	TACA	GABA	101.11
1156	Tetradecylthioacetic acid	101	TTA	Transcription	288.50
1157	Trequinsin hydrochloride	133	HL 725	Cyclic Nucleotides	441.96
1158	Tyrphostin AG 879	33	alpha-cyano-(3,5-di-t-butyl-4-hydroxy)thiocinnamide	Phosphorylation	316.47
1159	Tetraethylammonium chloride	108		Cholinergic	165.71
1160	Tolazamide	107		Hormone	311.41
1161	Terbutaline hemisulfate	119	2-t-Butylamino-1-(3,5-dihydroxyphenyl)ethanol	Adrenoceptor	548.66
1162	4-Hydroxyphenethylamine hydrochloride	121	Tyramine hydrochloride	Dopamine	173.64
1163	Triflupromazine hydrochloride	12		Dopamine	388.89
1164	Trimipramine maleate	13		Serotonin	410.52
1165	Tyrphostin AG 490	96	2-propenamide	Phosphorylation	294.31
1166	TTNPB	98	Arotinoid acid	Transcription	348.49
1167	L-765,314	112	(2S)-4-(4-Amino-6,7-dimethoxy-2-quinazolinyl)-2-[[(1,1-Dimethylethyl)amino]carbonyl]-1-piperazinecarboxylic acid, phenylmethyl ester	Adrenoceptor	522.61

1168	Triamterene	115		Na+ Channel	253.27
1169	Tyrphostin AG 1478	88	N-(3-Chlorophenyl)-6,7-dimethoxy-4-quinazolinamine	Phosphorylation	315.76
1170	Tetrahydrozoline hydrochloride	102		Adrenoceptor	236.75
1171	Tyrphostin AG 494	83	N-Phenyl-3,4-dihydroxybenzylidenecyanoacetamide	Phosphorylation	280.29
1172	N-p-Tosyl-L-phenylalanine chloromethyl ketone	83	TPCK	Biochemistry	351.85
1173	(6R)-5,6,7,8-Tetrahydro-L-biopterin hydrochloride	109		Neurotransmission	314.17
1174	Tyrphostin AG 527	81	Tyrphostin B44	Phosphorylation	308.34
1175	Theobromine	110	3,7-Dimethylxanthine	Adenosine	180.17
1176	(±)-Taxifolin	102	Dihydroquercetin	Cell Stress	304.26
1177	Tyrphostin AG 528	78	N-(3',4'-Dihydroxybenzylidenecyanoacetyl)-indoline	Phosphorylation	308.34
1178	Terazosin hydrochloride	107	1-(4-Amino-6,7-dimethoxy-2-quinazolinyl)-4-[(tetrahydro-2-furanyl)carbonyl]piperazine hydrochloride	Adrenoceptor	423.90
1179	Tyrphostin AG 537	112	Bis-Tyrphostin	Phosphorylation	448.44
1180	Tyrphostin AG 555	77	Tyrphostin B46	Phosphorylation	322.37
1181	Tyrphostin AG 698	82	Tyrphostin B52	Phosphorylation	308.34
1182	Tyrphostin AG 808	101	2-Cyano-3-(3',4'-dihydroxyphenyl)-1-(3''-indolyl)-3-oxo-1-propene	Phosphorylation	304.31
1183	Thio-NADP sodium	84	Thionicotinamide adenine dinucleotide phosphate sodium	Intracellular Calcium	781.46
1184	Tyrphostin AG 835	65	Tyrphostin B50	Phosphorylation	308.34
1185	(±)-alpha-Lipoic Acid	111	(±)-1,2-Dithiolane-3-pentanoic acid	Cell Stress	206.33
1186	DL-Thiorphan	107	DL-3-Mercapto-2-benzylpropanoylglycine	Neurotransmission	253.32
1187	Tulobuterol hydrochloride	104		Adrenoceptor	264.20
1188	Trazodone hydrochloride	97	2-[3-[4-(3-Chlorophenyl)-1-piperazinyl]propyl]- 1,2,4-triazolo[4,3-a]pyridin-3(2H)-one hydrochloride	Serotonin	408.33
1189	Tyrphostin AG 34	74	Tyrphostin A24	Phosphorylation	216.20
1190	Triamcinolone	108	Fluoxyprednisolone	Hormone	394.44
1191	S(-)-Timolol maleate	88	(S)-1-[(1,1-Dimethylethyl) amino]-3-[[4-(4-morpholinyl)-1,2,5-thiadiazol-3-yl]oxy]-2-propanol maleate	Adrenoceptor	432.50
1192	N,N,N-trimethyl-1-(4-trans-stilbenoxy)-2-propylammonium iodide	79	F3	Cholinergic	297.44
1193	Triprolidine hydrochloride	78	(E)-2-[3-(1-Pyrrolidinyl)-1-p-tolylpropenyl]pyridine hydrochloride	Histamine	314.86
1194	Tyrphostin AG 112	118	3-Amino-2,4-dicyano-5-(4'-hydroxyphenyl)-penta-2,4-dienonitrile	Phosphorylation	236.23
1195	Tyrphostin 1	81	(4-Methoxybenzylidene)malononitrile	Phosphorylation	184.20
1196	Tyrphostin 23	79	3,4-(Dihydroxybenzylidene)malononitrile	Phosphorylation	186.17
1197	TFPI hydrochloride	96	S-Ethyl N-(4-[Trifluoromethyl]phenyl)isothiourea	Nitric Oxide	284.73
1198	Na-p-Tosyl-L-lysine chloromethyl ketone hydrochloride	100	TLCK hydrochloride	Cyclic Nucleotides	369.31
1199	Tyrphostin 25	113	(4,5-Trihydroxybenzylidene)malononitrile	Phosphorylation	202.17
1200	1-[2-(Trifluoromethyl)phenyl]imidazole	86	TRIM	Nitric Oxide	212.18
1201	Taxol	84	Paclitaxel	Cytoskeleton and ECM	853.93
1202	Tetracaine hydrochloride	78		Na+ Channel	300.83
1203	Tyrphostin 47	61	RG 50864	Phosphorylation	220.25
1204	Tyrphostin 51	122	2-Amino-1,1,3-tricyano-4-(3',4',5'-trihydroxyphenyl)butadiene	Phosphorylation	268.23
1205	T-1032	60	Methyl (2-(4-aminophenyl)-1,2-dihydro-1-oxo-7-(2-pyridinylmethoxy)-4-(3,4,5-trimethoxyphenyl)-3-isoquinoline carboxylate sulfate	Cyclic Nucleotides	665.68
1206	I-OMe-Tyrphostin AG 538	96	alpha-Cyano-(3-methoxy-4-hydroxy-5-iodocinnamoyl)-(3',4'-dihydroxyphenyl)ketone	Phosphorylation	437.19
1207	Tyrphostin AG 538	75	(alphaE)-alpha-[(3,4-Dihydroxyphenyl)methylene]-3,4-dihydroxy-beta-oxo-benzenepropanenitrile	Phosphorylation	297.27
1208	Trimethoprim	92		Antibiotic	290.32
1209	Tomoxetine	79	(R)-N-methyl-gamma-(2-methylphenoxy)-benzenepropanamine	Adrenoceptor	255.36
1210	T-0156	56	2-(2-Methylpyridin-4-yl)methyl-4-(3,4,5-trimethoxyphenyl)-8-(pyrimidin-2-yl)methoxy-1,2-dihydro-1-oxo-2,7-naphthyridine-3-carboxylic acid methyl ester hyd	Cyclic Nucleotides	620.07
1211	3-Tropanyl-3,5-dichlorobenzoate	97	MDL-72222	Serotonin	314.21
1212	Trifluoperazine dihydrochloride	19		Dopamine	480.43
1213	D-609 potassium	87	Carbonodithioic acid, O-(octahydro-4,7-methano-1H-inden-5-yl) ester potassium	Lipid	266.47
1214	Thioridazine hydrochloride	14		Dopamine	407.04
1215	Thapsigargin	103		Intracellular Calcium	650.77
1216	Tyrphostin AG 126	100	(3-Hydroxy-4-nitrobenzylidene)malononitrile	Phosphorylation	215.17
1217	Tamoxifen citrate	87	(Z)-2-[4-(1,2-Diphenyl-1-butenyl)phenoxy]-N,N-dimethyl-ethanamine cirtrate (1:1)	Phosphorylation	563.65
1218	Terfenadine	41	alpha-(4-[1,1-Dimethylethyl]phenyl)-4-[hydroxydiphenylmethyl]-1-piperidinebutanol	Histamine	471.69
1219	Tropicamide	89	Ro 1-7683	Cholinergic	284.36
1220	THIP hydrochloride	95	Gaboxadol hydrochloride	GABA	176.60
1221	Trifluperidol hydrochloride	68	R 2498 hydrochloride; Triperidol hydrochloride	Dopamine	445.89
1222	3-Tropanyl-indole-3-carboxylate hydrochloride	89	ICS-205,930; Navoban; Tropisetron	Serotonin	320.82
1223	Tracazolate	113	4-(Butylamino)-1-ethyl-6-methyl-1H-pyrazolo [3,4-b]pyridine-5-carboxylic acid ethyl ester	GABA	304.40
1224	3-Tropanylindole-3-carboxylate methiodide	91		Serotonin	426.30
1225	Telenzepine dihydrochloride	77	4,9-Dihydro-3-methyl-4-[(4-methyl-1- piperazinyl)acetyl]-10H-thieno[3,4-b][1,5]benzodiazepin-10-one dihydrochloride	Cholinergic	443.40
1226	Thioperamide maleate	90	MR 12842	Histamine	408.52
1227	(±)-Thalidomide	107	(±)-2-(2,6-Dioxo-3-piperidinyl)-1H-isoindole-1,3(2H)-dione	Cytoskeleton and ECM	258.24
1228	R(+)-Terguride	73	R(+)-N,N-Diethyl-N'[(8alpha)-6-methylergolin-8-yl]urea	Dopamine	340.47
1229	Thiocitrulline	106	N5-(Aminothioxomethyl)-L-ornithine	Nitric Oxide	191.25
1230	Tyrphostin A9	25	[[3,5-bis(1,1-Dimethylethyl)-4-hydroxyphenyl]methylene]-propanedinitrile	Phosphorylation	282.39
1231	TPMPA	112	(1,2,5,6-Tetrahydropyridine-4-yl)methylphosphinic acid	GABA	161.14
1232	U-75302	107	6-[6-(3-Hydroxy-1E,5Z-undecadienyl)-2-pyridinyl]-1,5-hexanediol	Leukotriene	361.53
1233	Uridine 5'-diphosphate sodium	78	UDP	P2 Receptor	448.13
1234	U-74389G maleate	88	21-(4-[2,6-di-1-Pyrrolidinyl-4-pyrimidinyl]-1-piperazinyl)pregna-1,4,9[11]-triene-3,20-dione (Z)-2-butenedioate maleate	Cell Stress	726.92
1235	U-83836 dihydrochloride	103	(-)-2-([4-(2,6-Di-1-pyrrolidinyl-4-pyrimidinyl)-1-piperazinyl)methyl]-3,4-dihydro-2,5,7,8-tetramethyl-2H-1-benzopyran-6-ol dihydrochloride	Cell Stress	593.65
1236	U-73122	80	1-[6-[[(17beta)-3-Methoxyestra-1,3,5(10)-trien-17-yl]amino]hexyl]-1H-pyrrole-2,5-dione	Lipid	464.65

1237	SKF 95282 dimaleate	96	N-[3-[3-(1-Piperidinylmethyl)phenoxy]propyl]-2-benzothiazolamine dimaleate; Zolantidine	Histamine	613.69
1238	4-Imidazoleacrylic acid	74	Urocanic acid	Histamine	138.13
1239	Urapidil hydrochloride	94	6[[3-[4-(o-Methoxyphenyl)-1-piperazinyl]propyl]amino]-1,3-dimethyluracil hydrochloride	Adrenoceptor	423.95
1240	Urapidil, 5-Methyl-	91	5-Methyl-6[[3-[4-(o-Methoxyphenyl)-1-piperazinyl]propyl]amino]-1,3-dimethyluracil	Adrenoceptor	401.51
1241	U-69593	98	(+)-(5alpha,7alpha,8beta)-N-Methyl-N-[7-(1-pyrrolidinyl)-1-oxaspiro[4.5]dec-8-yl]-benzeneacetamide	Opioid	356.51
1242	UK 14,304	87	5-Bromo-N-(4,5-dihydro-1H-imidazol-2-yl)-6-quinoxalinamine	Adrenoceptor	292.14
1243	U-62066	90	Spiradoline mesylate	Opioid	521.51
1244	S(-)-UH-301 hydrochloride	90	S(-)-5-Fluoro-8-hydroxy-DPAT hydrochloride	Serotonin	301.84
1245	R(+)-UH-301 hydrochloride	97	R(+)-5-Fluoro-8-hydroxy-DPAT hydrochloride	Serotonin	301.84
1246	(+)-trans-(1R,2R)-U-50488 hydrochloride	116	trans-(1R,2R)-3,4-Dichloro-N-methyl-N-[2-(1-pyrrolidinyl)cyclohexyl]-benzeneacetamide hydrochloride	Opioid	405.80
1247	(-)-trans-(1S,2S)-U-50488 hydrochloride	105	trans-(1S,2S)-3,4-Dichloro-N-methyl-N-(2-[1-pyrollidinyl]cyclohexyl)benzeneacetamide hydrochloride	Opioid	405.80
1248	U-101958 maleate	105	1-Benzyl-4-aminomethyl-N-[(3'-isopropoxy)-2'-pyridinyl]piperidine maleate	Dopamine	455.56
1249	U-99194A maleate	97	5,6-Dimethoxy-2-(di-n-propylamino)indan maleate	Dopamine	393.48
1250	U0126	108	1,4-Diamino-2,3-dicyano-1,4-bis(o-aminophenylmercapto)butadiene	Phosphorylation	380.50
1251	Vinblastine sulfate salt	118	VLB	Cytoskeleton and ECM	909.07
1252	(±)-Verapamil hydrochloride	117		Ca2+ Channel	491.08
1253	VUF 5574	111	N-(2-methoxyphenyl)-N'-[2-(3-pyridinyl)-4-quinazolinyl]-urea	Adenosine	371.40
1254	Vinpocetine	148	Eburnamenine-14-carboxylic acid ethyl ester; (3alpha, 16alpha)-Eburnamenine-14-carboxylic acid ethyl ester	Cyclic Nucleotides	350.46
1255	Vancomycin hydrochloride from Streptomyces orientalis	117	Cancocin hydrochloride	Antibiotic	1485.75
1256	(±)-gamma-Vinyl GABA	108	Vigabatrin	GABA	129.16
1257	Vincristine sulfate	98	VCR	Cytoskeleton and ECM	923.06
1258	N-Vanillylnonanamide	90	N-(4-Hydroxy-3-methoxybenzyl)nonanamide	Vanilloid	293.41
1259	(±)-Vesamicol hydrochloride	97	AH-5183 hydrochloride	Cholinergic	295.86
1260	WHI-P131	90	4-(4'-hydroxylphenyl)-amino-6,7-dimethoxyquinazoline	Phosphorylation	333.78
1261	Wortmannin from Penicillium funiculosum	42		Phosphorylation	428.44
1262	1400W dihydrochloride	96		Nitric Oxide	250.17
1263	WB 64	83	N,N'-Tetramethyl-bis[(1,8-naphthylimid-9-yl)propyl-N,N'-hexane-1,6-diyl-bis-ammonium bromide	Cholinergic	808.66
1264	(R)-(+)-WIN 55,212-2 mesylate	58	(R)-(+)-[2,3-Dihydro-5-methyl-3[(morpholinyl)methyl]pyrrolo[1,2,3-de]-1,4-benzoxazinyl]-(1-naphthalenyl)methanone mesylate	Cannabinoid	522.63
1265	WIN 62,577	59	17-beta-Hydroxy-17-alpha-ethynyl-delta-4-androstano(3,2-b)pyrimido(1,2-a)benzimidazole	Tachykinin	438.58
1266	S(-)-Willardiine	99	S(-)-alpha-Amino-3,4-dihydro-2,4-dioxo-1(2H)-pyrimidinepropanoic acid	Glutamate	199.17
1267	WAY-100635 maleate	101	N-[2-[4-(2- Methoxyphenyl)-1-piperazinyl]ethyl]-N-2-pyridinyl-cyclohexanecarboxamide maleate	Serotonin	538.65
1268	S-5-Iodowillardiine	130	(S)-alpha-Amino-3,4-dihydro-5-iodo-2,4-dioxo-1(2H)-pyrimidinepropanoic acid	Glutamate	325.06
1269	Xylazine hydrochloride	134	N-(2,6-Dimethylphenyl)-5,6-dihydro-4H-1,3-thiazin-2-amine hydrochloride	Adrenoceptor	256.80
1270	Xamoterol hemifumarate	112	ICI 118587; (+/-)-N-[2-[[Hydroxy-3-(4-hydroxy)propyl]amino]ethyl-4-morpholinecarboxamide hemifumarate	Adrenoceptor	794.86
1271	Xylometazoline hydrochloride	85	2-(4-tert-Butyl-2,6-dimethylbenzyl)-2-imidazoline hydrochloride	Adrenoceptor	280.84
1272	Xanthine amine congener	90	8-[4-[[[[(2-Aminoethyl)amino]carbonyl]methyl]oxy]phenyl]-1,3-dipropylxanthine	Adenosine	428.50
1273	Yohimbine hydrochloride	85	17-Hydroxyyohimban-16-carboxylic acid methyl ester hydrochloride	Adrenoceptor	390.91
1274	YS-035 hydrochloride	93	N-[2-(3,4-Dimethoxyphenyl)ethyl]-3,4-dimethoxy-N-methyl-benzeneethanamine hydrochloride	Ca2+ Channel	395.93
1275	YC-1	136	3-(5'-Hydroxymethyl-2'-furyl)-1-benzyl indazole	Cyclic Nucleotides	304.35
1276	Zaprinast	115	1,4-Dihydro-5-(2-propoxyphenyl)-7H-1,2,3-triazolo[4,5-d]pyrimidin-7-one	Cyclic Nucleotides	271.28
1277	Zonisamide sodium	130	1,2-Benzisoxazole-3-methanesulfonamide	Anticonvulsant	234.21
1278	Zardaverine	125	6-(4-Difluoromethoxy-3-methoxyphenyl)-3(2H)-pyridazinone	Cyclic Nucleotides	268.22
1279	Zopiclone	99	Imovane	Benzodiazepine	388.82
1280	Zimelidine dihydrochloride	80	(Z)-3-(4-Bromophenyl)-N,N-dimethyl-3-(3-pyridinyl)-2-propen-1-amine dihydrochloride	Serotonin	390.15

NOTE: Shaded entries represent compounds identified as "hits" in the antagonist screen (see table 2) where a hit represents a compound effect equal to or less than the SCH23390 effect ±3 st. deviations (i.e., compound effect <20); †Percent receptor signaling in presence of test compound as measured by relative lux units relative to the SCH23390 control; ND, not determined - well did not contain sufficient test compound for assay
